# Influence of Limonene, Ethyl Lactate, and Diethyl Adipate on the Functional Properties of Chitosan-Based Films Loaded with *Fumaria officinalis* L. Extract and Propolis

**DOI:** 10.3390/antiox15101265

**Published:** 2026-10-01

**Authors:** Rabiea Ashowen Ahmoda, Milena Milošević, Tihomir Kovačević, Sanja Savić, Ivona Janković-Častvan, Boban Anđelković, Tamara Erceg, Aleksandra A. Jovanović, Aleksandar Marinković

**Affiliations:** 1 Faculty of Technology and Metallurgy, University of Belgrade, Karnegijeva 4, 11000 Belgrade, Serbia; 20214039@estudent.tmf.bg.ac.rs (R.A.A.); sseslija@tmf.bg.ac.rs (S.S.); icastvan@tmf.bg.ac.rs (I.J.-Č.); marinko@tmf.bg.ac.rs (A.M.); 2 Institute of Chemistry, Technology and Metallurgy—National Institute of the Republic of Serbia, University of Belgrade, Njegoševa 12, 11000 Belgrade, Serbia; milena.milosevic@ihtm.bg.ac.rs; 3 Military Technical Institute, Ratka Resanovića 1, 11030 Belgrade, Serbia; tkovacevic@tmf.bg.ac.rs; 4 Faculty of Chemistry, University of Belgrade, Studentski trg 12–16, 11000 Belgrade, Serbia; aboban@chem.bg.ac.rs; 5 Faculty of Technology Novi Sad, University of Novi Sad, Bulevar Cara Lazara 1, 21102 Novi Sad, Serbia; tamara.erceg@uns.ac.rs; 6 Institute for the Application of Nuclear Energy INEP, University of Belgrade, Banatska 31b, 11080 Belgrade, Serbia

**Keywords:** antioxidant potential, bio-based film, chitosan, *Fumaria officinalis* L., functional additives, plasticizers, polyphenols, propolis

## Abstract

The development of chitosan (CS)-based films incorporating antioxidant compounds offers a promising approach for the design of functional biobased materials. CS films containing propolis and *Fumaria officinalis* extract (FOE) were prepared with triglyceryl citrate (TGC) and modified with limonene, ethyl lactate (EL), or diethyl adipate (DEA); their physicochemical, release, and antioxidant properties were evaluated. EL- and DEA-containing films exhibited greater structural homogeneity than limonene-based; higher FOE concentrations increased surface heterogeneity and porosity. Dynamic mechanical analysis showed that the viscoelastic response depended on the additive, with CSEL retaining the highest moduli and CSL-FOE exhibiting the most pronounced softening after FOE/propolis incorporation (*G*′~10^5^ Pa at 100–110 °C; *G*″~10^4^ Pa at 120 °C). EL-based films retained the highest tensile resistance and the most balanced strength-deformability profile (stress at break: ~53.58 N/mm^2^; strain at break: ~14.05%); increasing FOE content reduced stress at break. Film wettability and water uptake were governed by additive and FOE concentration. The release study demonstrated sustained polyphenol delivery, with EL- and DEA-based systems showing approximately 50% release after 24 h. Antioxidant assays confirmed a time- and concentration-dependent increase in activity. The antioxidant response profiles differed among the films, with EL films showing an earlier increase in antioxidant activity, limonene films exhibiting a more gradual response, and DEA films displaying an intermediate response pattern.

## 1. Introduction

Additives, such as plasticizers, are a significant group of non-volatile low-molecular-weight compounds, widely used in the polymer industry to improve the flexibility and workability of polymer materials by lowering the second-order transition temperature, i.e., the glass transition temperature (*T_g_*) [1]. Plasticizers reduce stress during deformation, hardness, density, and viscosity, and increase polymer-chain mobility, fracture resistance, and dielectric constant. In addition, plasticizers can affect other polymer properties, including degree of crystallinity, optical transparency, combustion behavior, and resistance to biodegradation, as well as other physical properties [2]. As the need to replace conventional synthetic polymers and additives with natural and biodegradable materials grows, the need for sustainable, environmentally friendly alternatives to conventional plasticizers also increases. In this context, film-forming compounds and additives, obtained from renewable sources and often biodegradable, are attracting increasing attention due to their reduced toxicity, improved biocompatibility, and less negative environmental impact, while maintaining or improving the functional properties of polymer materials.

As fossil resources are finite, a transition toward renewable raw materials, including agricultural and municipal waste streams, together with advanced recycling strategies, has become increasingly important [3,4,5,6,7]. Therefore, chitosan (CS), a naturally derived biopolymer obtained from the deacetylation of chitin, plays a significant and increasingly important role [3,5,8,9]. Owing to its biodegradability, biocompatibility, inherent antimicrobial activity, and versatile chemical structure, CS has become one of the most extensively investigated polysaccharides [8,10,11,12,13]. Despite these advantages, CS-based materials have not yet achieved widespread large-scale applications, primarily because their mechanical and processing properties remain inferior to those of conventional petroleum-based polymers. The incorporation of suitable additives, particularly plasticizers, represents an effective strategy to tailor the physicochemical properties of CS films and enhance their flexibility, toughness, and processability. However, given that a key motivation for developing CS-based materials is reduced environmental impact and waste generation, the selected additives should also align with sustainability principles, including low toxicity, biodegradability, and, where possible, renewable sourcing. Previous studies have examined the effects of a limited number of biodegradable additives on CS films [14,15,16,17].

Ester-based plasticizers exhibit lower volatility and reduced hydrophilicity, resulting in improved compatibility with the CS matrix and enhanced resistance to plasticizer migration. These properties translate into better retention of mechanical properties during storage, improved elongation at break, and reduced moisture uptake. Consequently, ester plasticizers represent a promising, more durable alternative for mechanically stable, moisture-resistant CS films. Researchers have also investigated citrate-based plasticizers as bio-based, non-toxic additives for biopolymer films, where they effectively enhance flexibility by increasing polymer chain mobility and reducing intermolecular interactions [18]. The multiple hydroxyl groups and ester functionalities may promote good compatibility with polysaccharide matrices, such as CS and starch, and contribute to improved mechanical performance and film uniformity. Triglyceryl citrate (TGC) belongs to the class of citrate-based bio-plasticizers and can be used in biopolymer film systems because of its biodegradability, low toxicity, and ability to enhance flexibility and chain mobility in polysaccharide matrices [7,19,20]. Nevertheless, due to its relatively high molecular weight compared to low-molecular-weight plasticizers, such as glycerol, its plasticizing efficiency can be moderate, often requiring higher loading to achieve comparable flexibility.

Ethyl lactate (EL) can be investigated as an ester-based plasticizer for CS films due to its low toxicity, biodegradability, and good compatibility with polysaccharide matrices. It enhances polymer chain mobility, thereby improving the flexibility and reducing the brittleness of CS-based films, while also contributing to a more uniform dispersion of incorporated bioactive compounds. Namely, EL has been reported as a solvent and processing aid in biopolymer film formation, contributing to improved film homogeneity and mechanical performance in cellulose-based systems [21]. However, compared to more hydrophilic plasticizers such as glycerol, EL may exhibit a more moderate plasticization effect and can be associated with higher volatility, which may limit long-term stability in some applications. Diethyl adipate (DEA) is widely recognized as an alternative ester-based plasticizer used in polymeric and biopolymer films. It enhances flexibility by increasing polymer chain mobility and reducing intermolecular interactions. Its relatively low toxicity and good compatibility with polar polymer matrices make it a promising alternative to conventional phthalate plasticizers, particularly in biodegradable and food-contact materials. On the other hand, its plasticizing efficiency can be moderate compared to smaller-molecular-weight plasticizers such as glycerol, and its performance depends strongly on polymer composition and loading level [22,23].

Limonene is widely used as a natural additive in biopolymer films due to its strong antimicrobial and antioxidant activity. It can enhance the active functionality of materials and improve preservation. Namely, limonene is not considered a conventional plasticizer, but rather a matrix modifier incorporated into bio-based films, with only limited or indirect effects on the plasticization behavior of the polymer matrix. Nevertheless, its high volatility and hydrophobicity can lead to rapid loss from the polymer matrix and phase separation, which may reduce long-term stability and the film’s mechanical performance [24,25].

Considering these aspects, the present study investigates the influence of ester plasticizers, namely TGC, EL, and DEA, as well as the matrix modifier limonene (selected for their distinct molecular structures and low toxicity), on the mechanical, chemical, morphological, and antioxidant properties of CS films incorporating bioactives with antioxidant effects from propolis and *Fumaria officinalis*.

*Fumaria officinalis* L. (Papaveraceae), commonly known as common fumitory, is an annual herb traditionally used in ethnomedicine and increasingly investigated as a source of biologically active compounds. Phytochemical studies show that *F. officinalis* is particularly rich in isoquinoline alkaloids, mainly protopine-type, along with flavonoids and phenolic acids [26,27]. These metabolites are associated with pronounced antioxidant, anti-inflammatory, hepatoprotective, and antimicrobial activities, making fumitory a promising candidate for pharmaceutical, dermatological, and functional food applications [27,28]. Our previous LC-MS characterization confirmed a complex alkaloid and phenolic profile dominated by protopine derivatives, fumariline, and fumarophycine, as well as rutin, quercetin dihexoside, quercetin pentoside hexoside, methylquercetin dihexoside, and caffeoylmalic acid [29], supporting the suitability of fumitory extract as a multifunctional bioactive ingredient in polymer-based delivery systems.

Propolis, a resinous material collected by honeybees (*Apis mellifera*), is another well-established natural source of bioactive compounds with documented therapeutic potential. Although its composition varies by botanical and geographical origin, it is typically dominated by flavonoids, phenolic acids, and other aromatic compounds that collectively contribute to its strong antioxidant, anti-inflammatory, antimicrobial, and wound-healing properties [30,31,32]. Key constituents such as caffeic acid phenethyl ester, chrysin, galangin, and pinocembrin have been shown to modulate oxidative stress and immune responses, justifying the widespread incorporation of propolis into pharmaceutical, nutraceutical, and dermatological formulations [33,34].

Natural bioactives, such as polyphenols, are often limited in practical applications due to their low water solubility, poor absorption, potential toxicity, and consequently reduced bioavailability. Therefore, the development of various encapsulation strategies to overcome these limitations is necessary [35,36,37,38].

The selection of *F. officinalis* and propolis in this study was based on their distinct phytochemical profiles and reported biological activities, which motivated their incorporation into the same film matrix. However, despite the increasing use of natural bioactives and alternative plasticizing/modifying agents in CS-based films, the interactions among the additive, CS matrix, and incorporated bioactives remain insufficiently understood. Namely, it remains unclear how the chemical nature of the additive affects the structural organization, mechanical and water-related properties, release behavior, and preservation of antioxidant activity of CS films containing multiple bioactive compounds. This knowledge gap is particularly relevant for systems combining propolis and *F. officinalis* extract, for which the effects of ester-based additives and limonene on the balance between film performance and bioactive functionality have not been systematically compared.

Therefore, the present study investigates the influence of limonene, EL, and DEA on TGC-plasticized CS films incorporating propolis and *F. officinalis* bioactives. The hypotheses included: (i) the chemical nature of the additive would significantly affect the structural, mechanical, and water-related properties of the films; (ii) additive selection would modulate the release of polyphenolic compounds from the CS matrix; and (iii) the additive-matrix interactions would influence the retention and expression of the antioxidant activity of the incorporated bioactives. The formulation strategy was therefore designed to elucidate the relationships between additive type, film properties, bioactive release, and antioxidant functionality. Because antioxidant activity in polymeric delivery systems is closely related to the release and availability of incorporated phenolic compounds, chemical antioxidant assays can provide useful functional information regarding the antioxidant capacity of the released bioactive constituents. In the present study, DPPH, ABTS, and CUPRAC assays were therefore used as complementary chemical indicators of antioxidant capacity rather than as direct evidence of biological antioxidant mechanisms.

## 2. Materials and Methods

### 2.1. Materials

Citric acid, lactic acid, *p*-toluene sulfonic acid, ABTS (2,2′-azino-bis (3-ethylbenzothiazoline-6-sulphonic acid)), potassium persulfate, vitamin C (ascorbic acid), DPPH (2,2-diphenyl-1-picrylhydrazyl), d-limonene (≥95%), gallic acid, CuCl_2_·2H_2_O, ammonium acetate, neocuproine, Trolox, phosphate-buffered saline (PBS), Folin–Ciocalteu reagent, dimethyl sulfoxide (DMSO), DMSO-d6 (99.8%), and toluene were obtained from Sigma-Aldrich (St. Louis, MO, USA). Glucopon^®^ 600 CS UP solution (50% in H_2_O paste) was supplied by BASF SE (Ludwigshafen, Germany). Ethanol was purchased from Centrohem (Stara Pazova, Serbia). Glycerol, sodium hydrogen carbonate, sodium sulphate, methanol, sulphuric acid, adipic acid, sodium carbonate, S-(−)-ethyl lactate, CS (molecular weight 310–375 kDa, degree of deacetylation >75%), and sodium hydroxide were from Merck (Darmstadt, Germany). Plant material (fumitory herba, i.e., aerial part of the plant) was purchased from the pharmacy of the Institute for Medicinal Plant Research “Dr Josif Pančić”, Belgrade, Serbia. Propolis was purchased from Beekeeping Cooperative “Maja”, Belgrade, Serbia.

### 2.2. Synthesis of Plasticizers

#### 2.2.1. Synthesis of Triglyceryl Citrate

A 250 mL round-bottom flask equipped with a condenser, mechanical stirrer, thermometer, and Dean-Stark apparatus was charged with 5 g (0.024 mol) of citric acid, 7.50 g (0.081 mol) of glycerol, and 0.37 g (0.0019 mol) of *p*-toluene sulfonic acid as a catalyst (3 wt% relative to the total mass of reactants). Toluene was added to the Dean-Stark apparatus to collect the water formed during the reaction. The reaction mixture was heated to 160 °C, and the water produced was continuously removed until no water was collected, consistent with the reaction stoichiometry. After cooling, an aqueous solution of sodium hydrogen carbonate (0.18 g of salt and 10 mL of water, the same amount as the catalyst) was added, followed by methanol and anhydrous sodium sulphate. This step removed the catalyst from the mixture, and the anhydrous sodium sulphate was used to trap any remaining water. The purified contents of the flask were poured into a Petri dish and transferred to a rotary evaporator to remove residual methanol and water. Once the product reached sufficient viscosity, it was transferred to a glass container and dried under vacuum to remove remaining solvents and toluene. The results of ^1^H and ^13^C nuclear magnetic resonance (NMR) results (Figure S1) confirm the success of the synthesis of TGC.

#### 2.2.2. Synthesis of Diethyl Adipate

The synthesis of DEA was carried out according to a modified esterification procedure reported by Brewster and Ciotti [39]. Adipic acid (50 g), absolute ethanol (47.30 g, molar ratio 1:3), and 3 mL of concentrated sulfuric acid (as a catalyst) were placed into a round-bottom flask immersed in an oil bath. A Soxhlet extractor equipped with a thimble filled with anhydrous sodium sulphate was attached to the flask to capture the water formed during the reaction. The reaction mixture was stirred and heated at 120 °C for 20 h. After completion, the excess ethanol was distilled off, and the reaction mixture was transferred to a separatory funnel for purification. The contents were first washed with water, and the lower aqueous layer was discarded. A sodium hydrogen carbonate solution was then added; the mixture was shaken thoroughly, and the lower layer was again removed. Finally, the organic layer was washed once more with water and separated. The obtained organic phase was transferred into an Erlenmeyer flask containing anhydrous sodium sulphate to remove residual moisture. Once the sodium sulphate solidified, indicating complete water absorption, the liquid phase was decanted into another flask, yielding the purified DEA. The results of ^1^H and ^13^C NMR (Figure S2) confirm the success of the synthesis of DEA.

### 2.3. Extract Preparation

Dried fumitory herba was ground to obtain a fine powder (particle size of plant material <0.3 mm) and to achieve the highest extraction yield. The previously established protocol was used to prepare *Fumaria officinalis* extract (FOE) [29]. Specifically, 9 g of plant powder was mixed with 270 mL of 50% ethanol (*v*/*v*) (i.e., solid-to-solvent ratio of 1:30) and exposed to ultrasound in an ultrasound bath (DU-32, ARGO LAB, Carpi, Italy). The extraction process was performed in an Erlenmeyer flask covered with aluminum foil to prevent ethanol evaporation and light exposure for 15 min. During the extraction process, the flask was cooled with ice to prevent overheating of the sample. After that, the extract was filtered through fine filter paper, and the extraction medium was evaporated using a rotary evaporator (Heidolph Instruments, Schwabach, Germany) at 55 °C and 50 mbar for 30 min. The dried FOE was kept at 4 °C until further exploitation.

### 2.4. Propolis Purification

To obtain purified propolis, 10 g of propolis sample was cooled to −20 °C, ground using a mortar and pestle, and then extracted with 100 mL of 70% ethanol at 25 °C for 24 h in the dark. The samples were then cooled to 4 °C for 24 h and filtered through a precooled funnel. The filtrate was then evaporated to dryness. The dried sample was stored at 4 °C in the dark until analysis.

### 2.5. Determination of Total Polyphenol Content

The total polyphenol content (TPC) in purified propolis and FOE was determined using the Folin–Ciocalteu method [29]. Briefly, the sample was mixed with Folin–Ciocalteu reagent (diluted 1:2 with distilled water) and incubated for 5 min. Subsequently, Na_2_CO_3_ aqueous solution (10% *w*/*v*) was added to the mixture. The reaction mixture was kept at room temperature in the dark for 120 min to allow color development. Absorbance was measured at 765 nm using a UV-Vis spectrophotometer (Shimadzu UV-1800 UV-Vis spectrophotometer, Shimadzu, Kyoto, Japan). Gallic acid was used as a standard, and results were expressed as mg gallic acid equivalents per g of dried sample (mg GAE/g).

### 2.6. Preparation of Films

Nine types of CS-based films were prepared at varying FOE concentrations (4–8 wt%), while the concentrations of propolis, TGC, CS, lactic acid, Glucopon^®^, limonene, EL, and DEA were held constant. In the present study, limonene was considered a matrix modifier rather than a conventional plasticizer, whereas EL and DEA were considered plasticizing/modifying additives. Depending on the type of additive, the films were classified into three groups: films containing limonene (CSL-FOE (4 wt%), CSL-FOE (6 wt%), and CSL-FOE (8 wt%)), films containing EL (CSEL-FOE (4 wt%), CSEL-FOE (6 wt%), and CSEL-FOE (8 wt%)), and films containing DEA (CSDEA-FOE (4 wt%), CSDEA-FOE (6 wt%), and CSDEA-FOE (8 wt%)). Additionally, three control (plain) films containing only CS, lactic acid, Glucopon^®^, TGC with limonene, EL, or DEA (without FOE and propolis) were prepared. Preliminary experiments also examined films containing 2 wt% FOE; however, they showed only a marginal increase in antioxidant activity compared with the extract-free films. Therefore, these formulations were not included in the detailed comparative analysis presented in the main manuscript.

The film preparation procedure was performed in the following manner: 5 g of CS, 2 g of lactic acid, 1.5 g of the synthesized plasticizer (TGC), and 300 mL of distilled water were added to a 500 mL glass beaker and stirred at 50–55 °C until fully dissolved. TGC was incorporated into the aqueous CS-rich phase as the primary hydrophilic plasticizer. In contrast, limonene, EL, or DEA were introduced separately with propolis as secondary phase modifiers with different polarity and affinity toward the less polar components of the formulation. Glucopon^®^ was used as a nonionic emulsifier and compatibilizing agent to promote the dispersion and interaction of these components within the CS-based film-forming system. Separately, 1.3 g of raw or purified propolis was mixed with 1.5 g of the selected additive (limonene, EL, or DEA) and 0.4 g of Glucopon^®^ in 80 mL of ethanol for 30 min. FOE was added in three different proportions: 0.49 g (4 wt%), 0.75 g (6 wt%), and 1.02 g (8 wt%). The FOE content (4, 6, and 8 wt%) was calculated relative to the total mass of non-volatile film-forming components, including FOE. The mixture was stirred using a magnetic stirrer to enhance propolis dissolution. The solution containing propolis, the selected additive, and Glucopon^®^ was poured gradually into the CS solution under mixing using a mechanical stirrer (500 rpm), and the resulting film-forming opalescent solution, slightly turbid, was stirred at 25 °C for 1 h to attain homogeneity. The final film-forming solution was poured into Petri dishes and subjected to a two-stage drying procedure: 7 days at ambient temperature, followed by 4 h at 40 °C under reduced pressure (2000 Pa). To further distinguish the individual contributions of FOE and propolis to the antioxidant response, two additional CSEL-based control formulations were prepared. One formulation contained the same amount of FOE as the CSEL-FOE (6 wt%) film (0.75 g) but no propolis, whereas the second contained the same amount of purified propolis as the corresponding CSEL-FOE (6 wt%) formulation (1.3 g) but no FOE. These additional films were prepared under the same conditions as the other CSEL formulations and were used specifically for comparative DPPH analysis. Three independent batches of each film formulation were prepared for the study. The formulation design was established by keeping the concentrations of the main film-forming and auxiliary components constant, while varying the FOE concentration as the principal experimental variable. CS, lactic acid, TGC, propolis, Glucopon^®^, and the respective secondary additives (limonene, EL, or DEA) were therefore maintained at constant levels within the corresponding formulation groups, allowing the effect of FOE concentration to be evaluated without introducing additional compositional variables. The selected FOE concentrations (4, 6, and 8 wt%) were based on preliminary formulation trials and were chosen to provide a sufficiently broad concentration range for evaluating the effect of increasing FOE content while maintaining suitable film-forming properties. The formulations discussed in the main comparative study contained purified propolis. Corresponding films containing crude propolis were prepared for the comparative atomic force microscopy (AFM) and antioxidant analyses presented in the Supplementary Materials.

An additional semi-quantitative gravimetric comparison was performed with and without the respective secondary additive. Three independent measurements were performed for each formulation. Based on the difference in the final dry film mass, the apparent residual amounts corresponded to approximately 59% retention for limonene, 79% for EL, and 91% for DEA. These values were considered semi-quantitative, since residual moisture, solvent retention, and formulation-dependent differences in film structure may also contribute to the final film mass, and were therefore not used to recalculate the nominal formulation compositions.

Film designations used throughout the manuscript were defined according to the nominal composition of the initial film-forming formulations before drying. Accordingly, the 4, 6, and 8 wt% FOE labels refer to the nominal FOE contents calculated from the starting formulations and are retained as sample identifiers. Because partial loss of the secondary additives occurred during drying, the composition of the final dried films differs from the nominal formulation composition. The nominal compositions and the estimated final dried-film compositions, calculated from the apparent gravimetric retentions of limonene, EL, and DEA, are provided in Supplementary Tables S1 and S2, respectively.

### 2.7. Characterization Methods

#### 2.7.1. Nuclear Magnetic Resonance Spectroscopy

Structural verification of the synthesized plasticizers (TGC and DEA) was performed by ^1^H and ^13^C NMR spectroscopy using an Agilent/Varian 400-MR spectrometer (Agilent Technologies, Santa Clara, CA, USA), operating at 399.74 MHz for ^1^H and 100.53 MHz for ^13^C, and equipped with a 5 mm PFG ATB broadband probe. Measurements were performed at 298 K using D_2_O, CDCl_3_, or DMSO-d6, depending on the analyzed compound. Chemical shifts are reported as *δ* values in ppm and coupling constants (*J*) in Hz. The spectra were processed and analyzed using MestReNova software (version 14.2.0).

For the characterization of purified propolis and the identification and assignment of its individual components, one-dimensional (^1^H and ^13^C) NMR spectra were combined with two-dimensional NMR experiments, including 2D *J*-resolved, COSY, NOESY, HSQC, and HMBC spectroscopy. Selected assignments were additionally confirmed by comparison with spectra of corresponding reference standards or isolated compounds. These measurements were performed on a Bruker AVANCE III spectrometer (Bruker BioSpin, Rheinstetten, Germany) operating at 500.26 MHz for ^1^H and equipped with a 5 mm broadband observe (BBO) probe with a *z*-axis gradient. DMSO-d6 was used as the solvent. Spectral acquisition and processing were carried out using Bruker TopSpin software, versions 3.2 and 3.5.

For ^1^H NMR measurements, 32,768 data points were acquired using the standard “zg30” pulse program with 128 scans. The spectral width was set to 16 ppm (8012.82 Hz), with a relaxation delay (d1) of 2 s and an acquisition time of 2 s. The transmitter offset was set at 7 ppm, and the residual DMSO signal at 2.50 ppm was used as the chemical-shift reference.

For 2D *J*-resolved measurements, the spectral widths were 8012.82 Hz in the F2 dimension and 40 Hz in the F1 dimension. The relaxation delay was 2 s, while the acquisition times were 0.51 s in F2 and 0.8 s in F1. The spectra were acquired using 16 scans for each of 64 increments in the F1 dimension, with 8192 data points in F2. The transmitter offset was set at 7 ppm in both frequency dimensions. The total acquisition time was approximately 55 min.

#### 2.7.2. Fourier Transform Infrared Analysis

To analyze the chemical functional groups of the initial compounds, synthesized plasticizers, and resulting films, Fourier transform infrared (FTIR) spectroscopy was employed. Spectra were collected using a Nicolet™ iS™ 10 Spectrometer (Thermo Fisher Scientific, Waltham, MA, USA) outfitted with a Smart iTR™ Attenuated Total Reflectance (ATR) accessory. Data were acquired over the spectral range of 4000–500 cm^−1^ at a resolution of 4 cm^−1^, with each measurement of 20 scans. For comparative presentation, the original FTIR transmittance spectra were converted to absorbance, baseline-corrected, and normalized to the maximum absorbance. For peak deconvolution, the original absorbance spectra were analyzed separately in the 1800–1480 cm^−1^ region without prior normalization. A local linear baseline was subtracted, and the resulting spectra were fitted using four Gaussian components with the baseline offset fixed at zero. The same fitting procedure was applied consistently to all CSL, CSEL, and CSDEA films. Peak positions, integrated areas, and relative peak areas were obtained from the fitted components.

#### 2.7.3. Optical Microscopy

The surface morphology and structural characteristics of the CS-based films were analyzed using a Delta Optical Smart 5.0 MP digital microscope (Delta Optical, Nowe Osiny, Poland). To document the film’s micro-features, digital images were captured and processed using HiView software (HIRISE), version 2.3.

#### 2.7.4. Field Emission Scanning Electron Microscopy

The surface morphology of the films was examined using a field emission scanning electron microscope (FESEM), Tescan Mira 3 XMU FEG, equipped with a Schottky emission gun at 20 kV (TESCAN, Brno, Czech Republic). The samples were coated with an Au-Pd alloy to ensure conductivity.

#### 2.7.5. Atomic Force Microscopy

AFM measurements were carried out using a scanning probe microscope SPM-9700HT (Shimadzu, Kyoto, Japan). Imaging was performed in tapping mode, and height, amplitude, and phase images were recorded. Samples were placed on a sample holder and measured with a scan rate of 1 Hz and a resolution of 256 lines per scan direction. The measurements were performed using a silicon cantilever with the following characteristics: frequency of 320 kHz and force constant of 42 N/m. Different points on the sample surface were explored. The recorded images were just flattened, and no additional processing was performed.

#### 2.7.6. Dynamic Mechanical Analysis

The dynamic mechanical analysis (DMA) of the prepared films (rectangular bars with dimensions as follows: length: 54.0 mm, width: 10.0 mm, and thickness: 2.0 mm) was performed by a Modular Compact Rheometer MCR-302 (Anton Paar GmbH, Graz, Austria) equipped with standard fixtures SRF12, a temperature chamber (CTDe620) possessing high temperature stability (±0.1 °C), and automated cooling accessories containing liquid nitrogen. DMA measurements were performed using a temperature range of −30 °C to 145 °C with a heating rate of 5 °C·min^−1^ and a single angular frequency of 6.28 rad·s^−1^. For DMA measurements, separate film specimens were prepared from the same formulations using the same preparation and drying conditions as the other films. The only modification was an increased film thickness of approximately 2.0 mm, required to meet the dimensional requirements of the DMA setup. These specimens were therefore prepared separately from the thinner films used for thickness and tensile-property measurements.

#### 2.7.7. Determination of Thickness and Mechanical Properties

Film thickness was measured using a digital micrometer (Womax, Heidenheim, Germany) with 0.1 μm accuracy. The measurement was carried out at room temperature at three different locations on each film sample.

The tensile mechanical properties of the investigated samples were evaluated at ambient temperature using a Shimadzu Autograph AGS-X servo-hydraulic testing machine equipped with a 1 kN load cell (Shimadzu, Kyoto, Japan). Three measurements were performed for each sample at a testing rate of 2 mm min^−1^. Before testing, all samples were conditioned at 23 ± 2 °C and 50 ± 5% relative humidity for 48 h.

#### 2.7.8. Determination of Water Contact Angle

The wettability of the CS-based film surfaces was assessed using the sessile drop method. Distilled water droplets were gently placed onto the film surfaces at room temperature (25 °C). Contact angles were measured with Ossila Contact Angle software v1.3.0.0 (Ossila Ltd., Sheffield, UK), and the reported values represent the means of three independent measurements per sample to ensure reproducibility.

#### 2.7.9. Determination of a Film’s Swelling Ability

To evaluate the films’ moisture absorption capacity, a critical factor for potential contact applications, their swelling behavior was characterized by measuring mass changes during liquid immersion. The swelling behavior of the films was evaluated by immersion in PBS (pH 7.0) at room temperature. Initially, the dry films were weighed to determine their initial mass (W_0_). Subsequently, the samples were immersed in PBS solution at room temperature for a defined period. After immersion, at predetermined intervals (30, 60, 90, 120, and 150 min), the films were carefully removed from the solution. Excess surface liquid was gently blotted off with filter paper before weighing (W_t_). The swelling capacity was expressed as the swelling ratio (SR), calculated according to the following equation (Equation (1)):

(1) $$\mathrm{SR}=\frac{W_{t}-W_{0}}{W_{0}},$$

where W_0_ represents the initial dry weight of the film, and W_t_ corresponds to the weight of the swollen film after immersion in PBS solution. Independent film specimens were used for each incubation time point; therefore, measurements at different time points did not represent repeated measurements of the same specimen.

### 2.8. Release Kinetic Study

The in vitro release study was performed using a Franz diffusion cell (PermeGear Inc., Hellertown, PA, USA) [40]. Namely, 2 g of biobased film with propolis and FOE was placed on the cellulose acetate membrane (Sartorius Stedim Biotech GmbH, Göttingen, Germany) between the donor and acceptor compartments. The acceptor compartment contained 20 mL of PBS (pH 7) at room temperature. The samples (500 µL) were taken at defined time intervals, and the polyphenol content was determined spectrophotometrically at 320 nm. The concentration of released polyphenols was calculated from a calibration curve prepared using gallic acid as the reference standard. At each sampling time, a 500 µL aliquot was withdrawn from the acceptor compartment, used for spectrophotometric determination, and subsequently returned to the acceptor compartment, thereby maintaining a constant acceptor volume throughout the experiment. The amount of polyphenols released at each sampling time was calculated from the measured GAE concentration and the total acceptor volume. The cumulative amount of released polyphenols was then expressed as a percentage of the total polyphenol content initially present in the corresponding film. The initial total polyphenol content of each film was calculated based on the experimentally determined total phenolic content of the FOE and purified propolis and the respective amounts incorporated into the film formulation. Thus, 100% release corresponds to the total experimentally determined polyphenol content initially present in the respective film. The data obtained are presented as the percentage of the released polyphenols. The zero-order, first-order, and Higuchi models were fitted over the 0–120 min interval, whereas the Korsmeyer-Peppas model was applied to the early release region from 5 to 120 min. The 1440 min point was retained for characterization of the 24 h cumulative release but was not included in the kinetic fitting because of the large sampling interval between 120 and 1440 min. Nonlinear fitting was performed using OriginPro 2018 (OriginLab Corporation, Northampton, MA, USA). Model parameters are reported as fitted values ± standard errors, and the quality of fit was evaluated using the coefficient of determination (*R*^2^). For kinetic analysis, the released fraction *F_t_* was calculated by dividing the percentage of released polyphenols by 100.

### 2.9. Determination of the Antioxidant Potential

#### 2.9.1. DPPH• Radical Scavenging Assay

The antioxidant analysis was performed by measuring the change in absorbance of the DPPH• radical at 517 nm [41]. Film samples (6 mg) were immersed in 40 mL of PBS (pH 7, room temperature). After that, at 5, 10, 15, 30, 45, 60, 90, and 120 min, as well as 24 h, 1 mL aliquots of the film solutions were collected. To determine the activity of the released components, the samples were diluted using PBS. At each sampling time point, 1 mL of the release medium was withdrawn for analysis and immediately replaced with an equal volume of fresh PBS to maintain a constant release-medium volume throughout the experiment. Commercially available DPPH• radical (7 mg) was dissolved in ethanol (250 mL). The reaction mixture consisted of 2.8 mL of DPPH• solution and 200 μL of the film sample solution. A mixture of 200 μL of pure PBS and 2.8 mL of DPPH• solution served as the control, while ascorbic acid was used as the reference standard for antioxidant activity. The absorbance at 517 nm was recorded after incubation in the dark for 30 min at room temperature. All measurements were performed in triplicate, and the percentage of DPPH• radical scavenging was calculated using the following equation (Equation (2)):

(2) $$Inhibition\left(\%\right)=\frac{A_{cDPPH}-A_{sDPPH}}{A_{cDPPH}}\times 100,$$

where A_sDPPH_ and A_cDPPH_ represent the absorbance values of the DPPH• solution with the developed film and the control solution, respectively. The results are expressed as the percentage of DPPH• radical inhibitory potential. For each film formulation, a corresponding blank film containing the same CS matrix and matrix modifier, but without FOE and purified propolis, was prepared and evaluated as a control. The results obtained for the blank films were included in the antioxidant activity analysis to assess the intrinsic contribution of the film matrix and matrix modifier to the measured DPPH radical-scavenging response. In addition, to assess the individual contribution of FOE and propolis to the anti-DPPH activity, additional CSEL films corresponding to the 6 wt% FOE formulation were prepared containing either FOE alone, without propolis, or purified propolis alone, without FOE.

#### 2.9.2. ABTS•^+^ Radical Scavenging Assay

The analysis was performed by measuring the change in absorbance of the ABTS•^+^ radical at 734 nm [42]. The ABTS radical cation (ABTS•^+^) solution was prepared by mixing 7.8 mM ABTS stock solution with 2.45 mM potassium persulfate. The working solution was allowed to react for 12–16 h to generate ABTS•^+^ radicals and then diluted with PBS to achieve an absorbance of 0.700 ± 0.020 at 734 nm. Film samples were prepared as described above for the DPPH assay. The reaction mixture consisted of 2.8 mL of ABTS•^+^ solution and 200 µL of the film samples. As a control, 200 µL of pure PBS was added to 2.8 mL of ABTS•^+^ solution. After 6 min incubation in the dark at room temperature, absorbance was measured at 734 nm using a UV/Vis spectrophotometer. Ascorbic acid was used as a standard. All measurements were performed in triplicate. The inhibition of the ABTS•^+^ radical cation was calculated according to the following equation (Equation (3)):

(3) $$Inhibition\left(\%\right)=\frac{A_{cABTS}-A_{sABTS}}{A_{cABTS}}\times 100,$$

where A_sABTS_ and A_cABTS_ represent the absorbance values of the ABTS•^+^ solution with the developed film and the control solution, respectively. The results are expressed as the percentage of ABTS•^+^ radical inhibition. Corresponding blank films containing the same CS matrix and matrix modifier, but without FOE and purified propolis, were prepared and evaluated as controls. These blank-film controls were used to assess the intrinsic contribution of the film matrix and matrix modifier to the measured ABTS radical scavenging response.

#### 2.9.3. Cupric Ion Reducing Power Assay

The cupric ion reducing antioxidant capacity (CUPRAC) test was performed according to the previously described method [43]. The 1.0 × 10^−2^ M Cu(II) solution was prepared by dissolving 0.08524 g CuCl_2_ × 2H_2_O in water and diluting to 250 mL. An ammonium acetate buffer at pH 7.0, 1.0 M was prepared by dissolving 19.27 g of ammonium acetate in water and diluting to 250 mL. Neocuproine solution (7.5 × 10^−3^ M) was prepared by dissolving 0.078 g of neocuproine in methanol and diluting to 50 mL with methanol. This solution was freshly prepared. Additionally, a 5 × 10^−3^ M Trolox solution was prepared by dissolving 0.00626 g of Trolox in DMSO and diluting it to five concentrations. Each reaction mixture consisted of 0.8 mL of the tested compound solution in PBS, 1 mL of CuCl_2_ × 2H_2_O solution, 1.2 mL of ammonium acetate buffer solution, and 1 mL of neocuproine solution in methanol. After incubation at room temperature for 30 min, the absorbance at 450 nm was recorded on a UV-Vis spectrophotometer. The results were calculated using a standard calibration curve for Trolox (0.01–1.25 mM) and were expressed in millimoles of Trolox equivalents per g of film (mM TE/g film). Corresponding blank films containing the same CS matrix and matrix modifier, but without FOE and purified propolis, were prepared and evaluated as controls. These blank-film controls were used to assess the intrinsic contribution of the film matrix and matrix modifier to the measured ion-reducing potential.

### 2.10. Statistical Analysis

Statistical analysis was performed using analysis of variance (ANOVA), with the statistical model selected according to the experimental design. For factorial experiments, two-way ANOVA was used to evaluate the effects of additive type, FOE concentration, and their interaction on the mechanical properties, water contact angle, and SR of the films. For the factorial analysis, only the formulations containing 4, 6, and 8 wt% FOE were considered, as these formulations contained a constant amount of propolis. Thus, two-way ANOVA was used to assess the effects of additive type, FOE concentration, and their interaction. The blank films, which contained neither FOE nor propolis, were not considered as the 0 wt% FOE level and were analyzed separately. For time-dependent swelling experiments, the formulation factors were analyzed using two-way ANOVA at the corresponding experimental conditions. Where significant differences were detected, Tukey’s post hoc test was applied at a significance level of *p* < 0.05. Quantitative measurements subjected to statistical analysis were performed in triplicate. The release and antioxidant activity data were evaluated descriptively to characterize their time-dependent profiles and trends and were not subjected to statistical analysis.

## 3. Results and Discussion

### 3.1. Nuclear Magnetic Resonance Data

**Figure 1:**
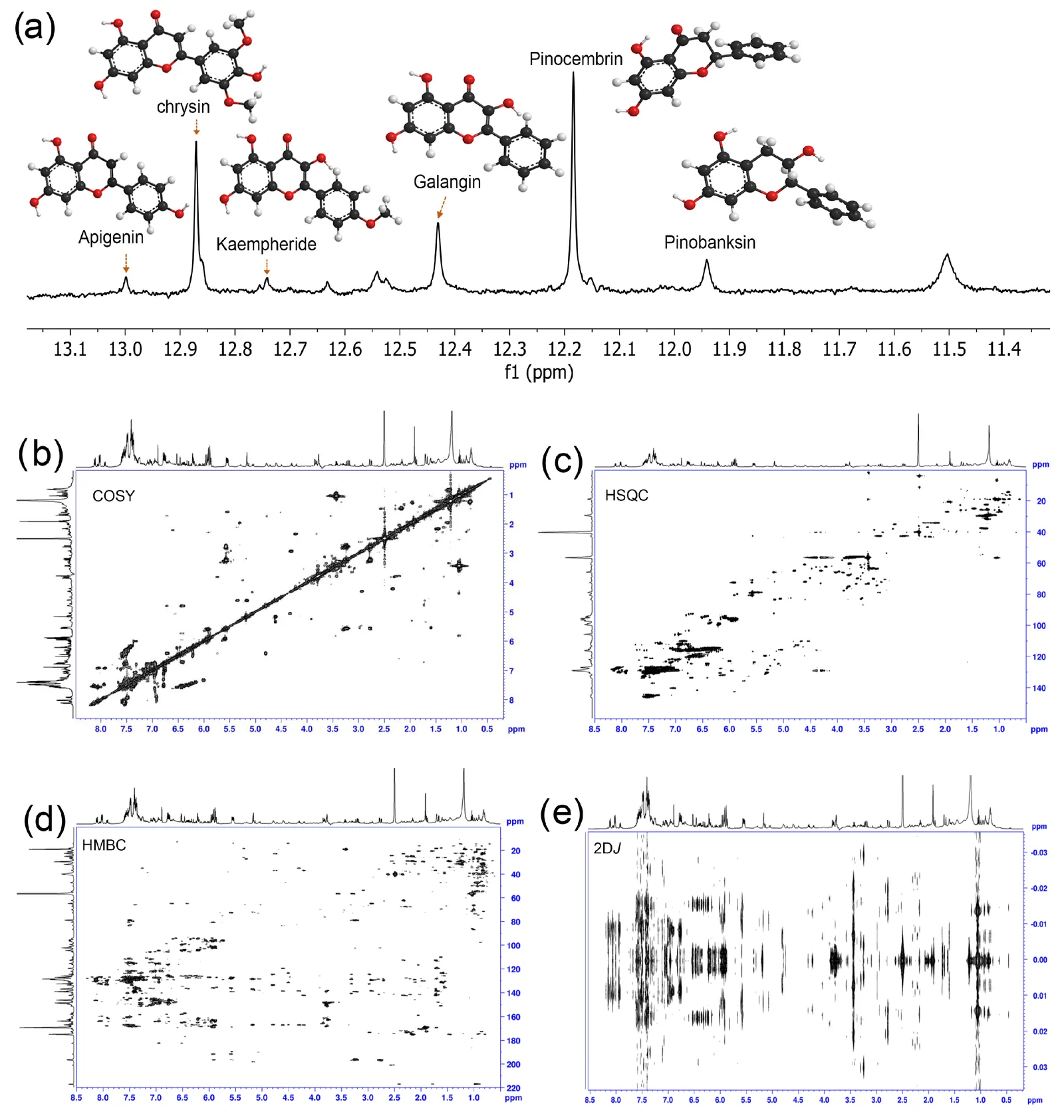
(**a**) ^1^H nuclear magnetic resonance (NMR) spectrum of propolis, (**b**) COSY, (**c**) HSQC, (**d**) HMBC, and (**e**) 2D *J*-resolved spectra.

1D and 2D NMR analyses were used to characterize and tentatively assign the major constituents of propolis. The identification of chrysin, galangin, pinocembrin, and pinobanksin 3-*O*-acetate was confirmed based on in-house reference standards [44]. The ^1^H NMR spectrum (Figure 1a) of the propolis extract showed numerous overlapping aromatic, olefinic, oxygenated aliphatic, and aliphatic resonances. To facilitate signal assignment, COSY, HSQC, HMBC, and *J*-resolved spectra (Figure 1b–e) were analyzed in detail.

The propolis extract compounds were assigned based on the combined 1D and 2D NMR data and are presented in Table 1.

The signals observed between *δ* 11.4 and 13.1 ppm (Figure 1a and Table 1) were attributed to intramolecularly hydrogen-bonded hydroxyl protons of flavonoids. The signals at *δ* 12.96, 12.82, 12.69, 12.36, 12.12, and 11.89 ppm were assigned to apigenin, chrysin, kaempferide, galangin, pinocembrin, and pinobanksin, respectively. An additional resonance at approximately *δ* 11.44 ppm was consistent with pinobanksin 3-*O*-acetate (Table 1). The assignments were further supported by comparison of the aromatic and aliphatic resonances with published NMR data [44]. Signals characterized by large coupling constants (*J*) of approximately 15.9–16.0 Hz indicated *trans*-configured cinnamoyl units and supported the presence of caffeic acid, *p*-coumaric acid, ferulic acid, and their corresponding ester derivatives [44]. Benzyl caffeate was suggested by the benzylic resonance at *δ* 5.19 ppm, whereas caffeic acid phenethyl ester was supported by signals at *δ* 4.31 and 2.94 ppm. The resonances at *δ* 4.80, 6.42, 6.71, and 7.46 ppm were consistent with the cinnamyl moiety of cinnamyl caffeate. The COSY spectrum (Figure 1b) revealed characteristic proton spin systems belonging to flavonoids and phenylpropanoid derivatives. Correlations between the olefinic proton pairs characterized by large coupling constants supported the presence of *trans*-cinnamoyl moieties, while correlations between H-2 and the diastereotopic H-3 protons were consistent with the flavanone skeletons of pinocembrin and naringenin. The HSQC spectrum (Figure 1c) confirmed the presence of aromatic and olefinic methine groups, oxygenated methine and methylene groups, methoxy substituents, and aliphatic methylene groups. Long-range HMBC correlations (Figure 1d) of aromatic and olefinic protons with oxygenated aromatic carbons and ester or flavonoid carbonyl carbons further supported the proposed assignments. The 2D *J*-resolved spectrum (Figure 1e) helped determine signal multiplicities and coupling constants in the strongly overlapping regions of the ^1^H NMR spectrum. Based on the combined spectral evidence, chrysin, galangin, pinocembrin, and pinobanksin 3-*O*-acetate were confirmed by comparison with in reference standards [44], whereas pinobanksin, pinobanksin 3-*O*-methyl ether, kaempferide, apigenin, naringenin, caffeic acid, *p*-coumaric acid, ferulic acid, benzoic acid, benzyl caffeate, caffeic acid phenethyl ester, and cinnamyl caffeate were tentatively identified. The simultaneous presence of chrysin, galangin, pinocembrin, pinobanksin, and pinobanksin 3-*O*-acetate indicated that the investigated sample exhibited a chemical profile characteristic of a system rich in flavonoids. Because of the extensive spectral overlap characteristic of propolis extracts, the compounds were regarded as tentatively assigned of their ^1^H NMR chemical shifts, multiplicities, coupling constants, and comparison with literature data.

### 3.2. Fourier Transform Infrared Spectroscopy Data

**Figure 2:**
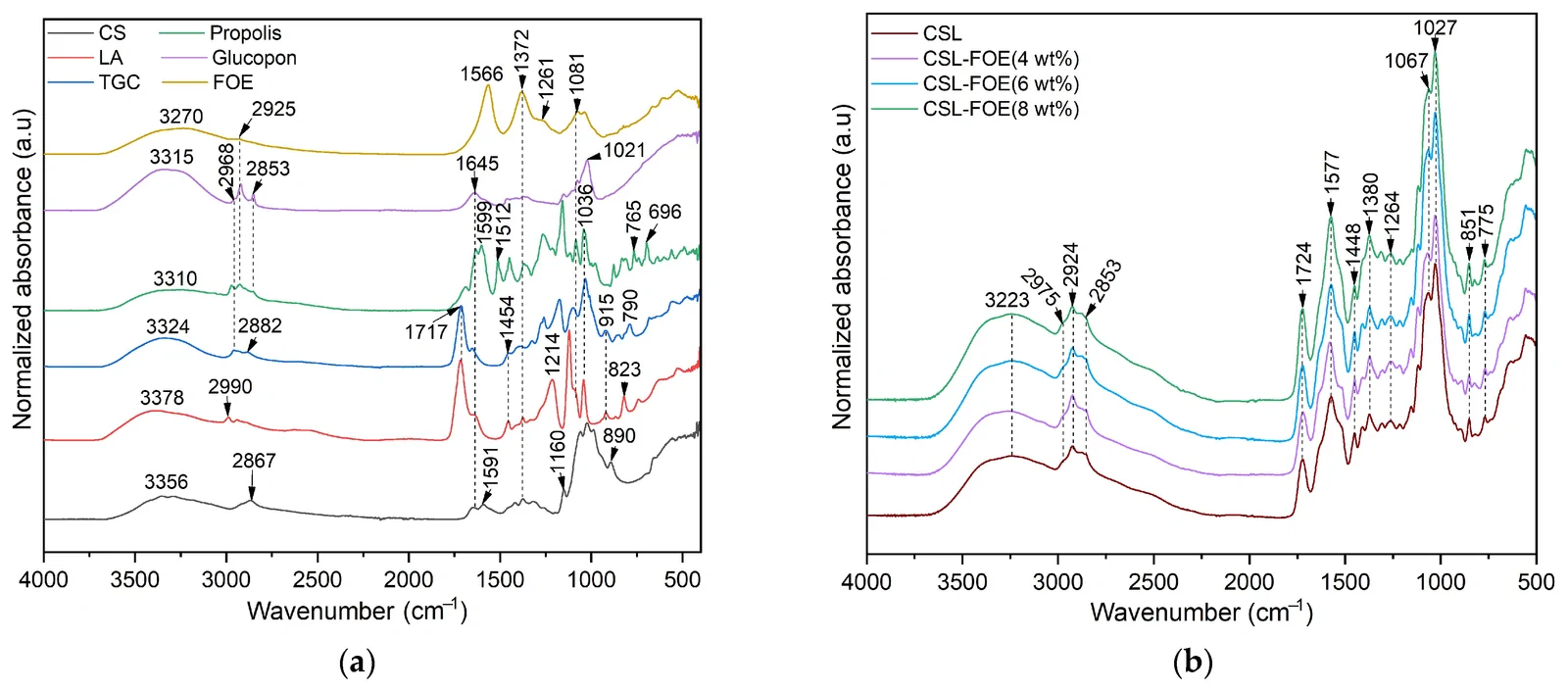

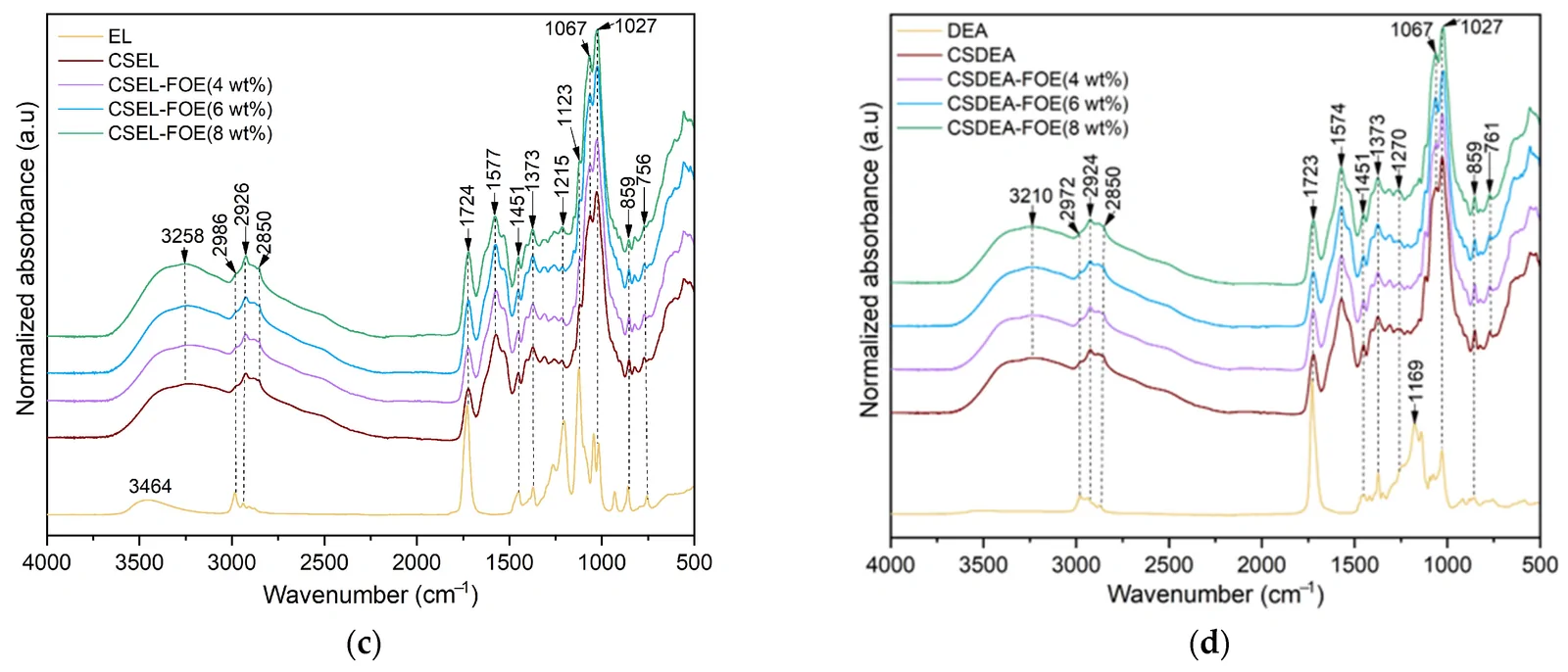
Fourier transform infrared spectra of (**a**) film constituents, (**b**) CSL-FOE (4–8 wt%), (**c**) CSEL-FOE (4–8 wt%), and (**d**) CSDEA-FOE (4–8 wt%) films; CS—chitosan, LA—lactic acid, TGC—triglyceryl citrate, FOE—*Fumaria officinalis* extract, L—limonene, EL—ethyl lactate and DEA—diethyl adipate.

FTIR spectroscopy was used to investigate potential functional group interactions within the film compounds, including CS, TGC, purified propolis, FOE, lactic acid, and the emulsifier Glucopon^®^, as well as limonene, EL, and DEA. The FTIR spectra of these compounds and films are displayed in Figure 2. Additionally, the FTIR spectra of crude propolis, purified propolis, and the obtained residue are displayed in Supplementary Materials (Figure S3).

The FTIR spectrum of CS (Figure 2a) shows absorption bands at 3354 cm^−1^ and 3290 cm^−1^ corresponding to N–H and O–H stretching vibrations, as well as intramolecular hydrogen bonds [45]. The peaks at around 2867 cm^−1^ are attributed to C–H stretching, while the deformations were observed at 1454–1315 cm^−1^. Two bands at 1645 cm^−1^ and 1591 cm^−1^ correspond to C=O stretching vibrations (amide I) and the N–H bending of the primary amine [45]. A set of characteristic saccharide bands, such as C–O–C and C–O stretching, is situated in the range of 1149–920 cm^−1^. Figure 2a also shows that the lactic acid spectrum has a characteristic peak at 1717 cm^−1^, corresponding to the C=O stretching vibration of carboxyl groups. A set of characteristic peaks for lactic acid is observed in the spectral region around 3378 cm^−1^ and 1645 cm^−1^, 2990–2925 cm^−1^, 1454–1374 cm^−1^, and 1214–1034 cm^−1^, which belong to hydroxyl (OH stretching and bending), alkyl (C-H stretching and bending), and ether (C–O–C/C–O stretching), respectively. In the spectrum of the purified propolis extract (Figure 2a and Figure S3), the bands observed at around 3324 cm^−1^, 1690 cm^−1^, 1645 cm^−1^, and 1599–1512 cm^−1^ attributed to the vibrations of O–H and N–H stretching band, C=O stretching band of ester group, C=O stretching vibrations of carboxyl, and C=C stretching of aromatic/unsaturated hydrocarbons compounds indicate the presence of different compounds in propolis like phenolics, alcohols, organic acids, amino acids, terpenes, etc. [46]. C–H stretching vibration of alkyl compounds was observed between 2968 cm^−1^ and 2853 cm^−1^, suggesting the presence of aliphatic hydrocarbons and flavonoid components [46]. Further, peaks in the region 1450–1372 cm^−1^ indicated C-H bending vibration of alkyl groups. The 1261–980 cm^−1^ region signifies C–O–C/C–O stretching in primary alcohols, phenolic esters, and glycosidic bonds. The 870–696 cm^−1^ region would represent out-of-plane C–H bending in aromatic rings in flavonoids or unsaturated hydrocarbons. Additionally, the main functional groups present in crude propolis and in the residue after extraction were analyzed and discussed in detail in the Supplementary Materials (Figure S3). The similarity of the main FTIR bands suggests that the functional-group profile was largely preserved during purification, whereas differences in the residue spectrum are consistent with the removal of less polar components, particularly waxy and lipid fractions (Supplementary Materials, Figure S3) [47]. The spectrum of the residue after extraction shows a different profile. The bands in the range 3500–790 cm^−1^ (Figure S3) may be related to aromatic structures, carbonyl and aliphatic compounds, most likely esters from waxes and lipid components. This result indicates that the purification process selectively enriched the extract with functional groups characteristic of phenolic compounds, while the residue after extraction was enriched in less polar components, particularly waxes and lipid structures. The FTIR spectra of FOE (Figure 2a) had a broad band at 3213 cm^−1^ assigned to O–H and N–H stretching vibrations of hydroxyl, phenolic, carboxyl, and amino groups from compounds in the extract [29]. Vibration peaks at 2925 cm^−1^ and 1372 cm^−1^ are assigned to C–H stretching vibrations of aliphatic methylene and methyl groups, showing stretching and bending vibrations in the range 2923–2853 cm^−1^ and 1389–1310 cm^−1^, respectively. The low intensity peaks at 1707 cm^−1^ and 1585 cm^−1^ are assigned to stretching vibrations of the carboxyl group in phenolic acids related structure and to overlapping aromatic C=C stretching vibrations with possible contributions from nitrogen-containing constituents of the complex extract [29]. The wavenumber region at 1261–915 cm^−1^ and 840–613 cm^−1^ is characteristic of the absorption of asymmetric and symmetric vibrations of C–O, C–O–C, and C–O–H groups and C–H out-of-plane deformational vibrations of aromatic and vinyl parts in the structures of active compounds in the extract, respectively [29]. In conclusion, the FTIR profile of the extract exhibited characteristic bands of polyphenolic compounds, including broad O–H stretching vibrations (~3300 cm^−1^), aromatic C=C vibrations (~1600 cm^−1^), and C–O/C–O–C stretching vibrations in the fingerprint region (~1260–1000 cm^−1^), similarly reported for plant extracts rich in flavonoids and tannins [29,48]. FTIR analysis of the used emulsifier Glucopon^®^ (Figure 2a) shows characteristic peaks of the glucoside ring, for example, around 3315 cm^−1^, 2968–2853 cm^−1^, and 1645 cm^−1^, corresponding to vibrations of hydroxyl groups, C–H stretching from –CH_2_, and O–H bending, respectively. Further, polysaccharide bands C–O and C–O–C appear at 1157–915 cm^−1^.

The FTIR spectra of the films showed similar overall profiles, with formulation-dependent differences in band position and shape. Also, the spectra of all films (Figure 2b–d) show characteristic bands attributable to the principal functional groups of the film components. The observed spectral features may reflect changes in hydrogen-bonding and other intermolecular associations among the film components. Accordingly, the spectra of CSL-FOE (4–8 wt%) films (Figure 2b) presented high-intensity bands associated with C=O, N–H bending (amide II), C–H stretching and bending vibrations, C–O/C–O–C stretching vibrations, and out-of-plane C–H bending vibrations at 1724 cm^−1^, 1581 cm^−1^, 2975–2853 cm^−1^, 1448–1315 cm^−1^, 1264–1027 cm^−1^, and 851–580 cm^−1^, respectively. The FTIR spectra of the CSEL-FOE and CSDEA-FOE films displayed in Figure 2c and Figure 2d, respectively, are similar to the patterns of the CSL-FOE films. For all film series, increasing FOE concentration from 4 to 8 wt% preserved the overall spectral profile, while subtle changes in band position and shape were observed. Similar FTIR features were previously reported for polysaccharide-based systems and plant-metabolites-loaded formulations [48]. Namely, broad bands around 3300 cm^−1^ were attributed to O–H stretching vibrations, while bands in the regions 2920–2850 cm^−1^, 1700–1600 cm^−1^, and 1260–1000 cm^−1^ were associated with C–H, C=O/C=C, and C–O/C–O–C vibrations, respectively, supporting the presence of polyphenolic and polysaccharide structures and suggesting possible intermolecular associations within the matrix [48].

To provide a quantitative assessment of the overlapping bands, the 1800–1480 cm^−1^ region was additionally subjected to peak deconvolution using four Gaussian components (Figure S4 and Table S3). Across the CSL series, comparison of the blank CSL film with the FOE/propolis-containing formulations showed a shift of the carbonyl-related component from 1725 cm^−1^ in CSL to 1721 cm^−1^ in CSL-FOE (8 wt%), while the components at 1638, 1576, and 1520 cm^−1^ shifted to 1640, 1579, and 1523 cm^−1^, respectively. Within the FOE/propolis-containing formulations, progressive but relatively small spectral changes were observed with increasing FOE loading. These changes were accompanied by a moderate redistribution of the relative peak areas, particularly a decrease in the dominant 1576–1579 cm^−1^ component from 57.42 to 54.36%. In the CSEL series, comparison of the blank CSEL film with the FOE/propolis-containing formulations showed that the bands at 1724 and 1634 cm^−1^ remained essentially unchanged, whereas the lower-wavenumber components shifted from 1573 to 1576 cm^−1^ and from 1520 to 1525 cm^−1^. This series exhibited the most pronounced redistribution of the deconvoluted band areas, with the relative contribution of the 1573–1576 cm^−1^ component decreasing from 51.63 to 35.44%, together with increases in the 1724 and 1634 cm^−1^ components. In contrast, comparison of the blank CSDEA film with the corresponding FOE/propolis-containing formulations revealed only minor changes in both peak positions and relative areas, with the resolved bands remaining within 1724, 1632–1633, 1572–1574, and 1523–1524 cm^−1^. The deconvolution revealed additive-dependent changes in the local spectral environment, most pronounced in the CSEL films, moderate in the CSL films, and comparatively limited in the CSDEA series. These spectral changes are consistent with changes in intermolecular associations within the multicomponent CS matrix; however, because of the extensive overlap of the contributing vibrational modes, they are considered supportive rather than definitive evidence of specific intermolecular interactions.

### 3.3. Optical Microscopy Characterization of Developed Films

**Figure 3:**
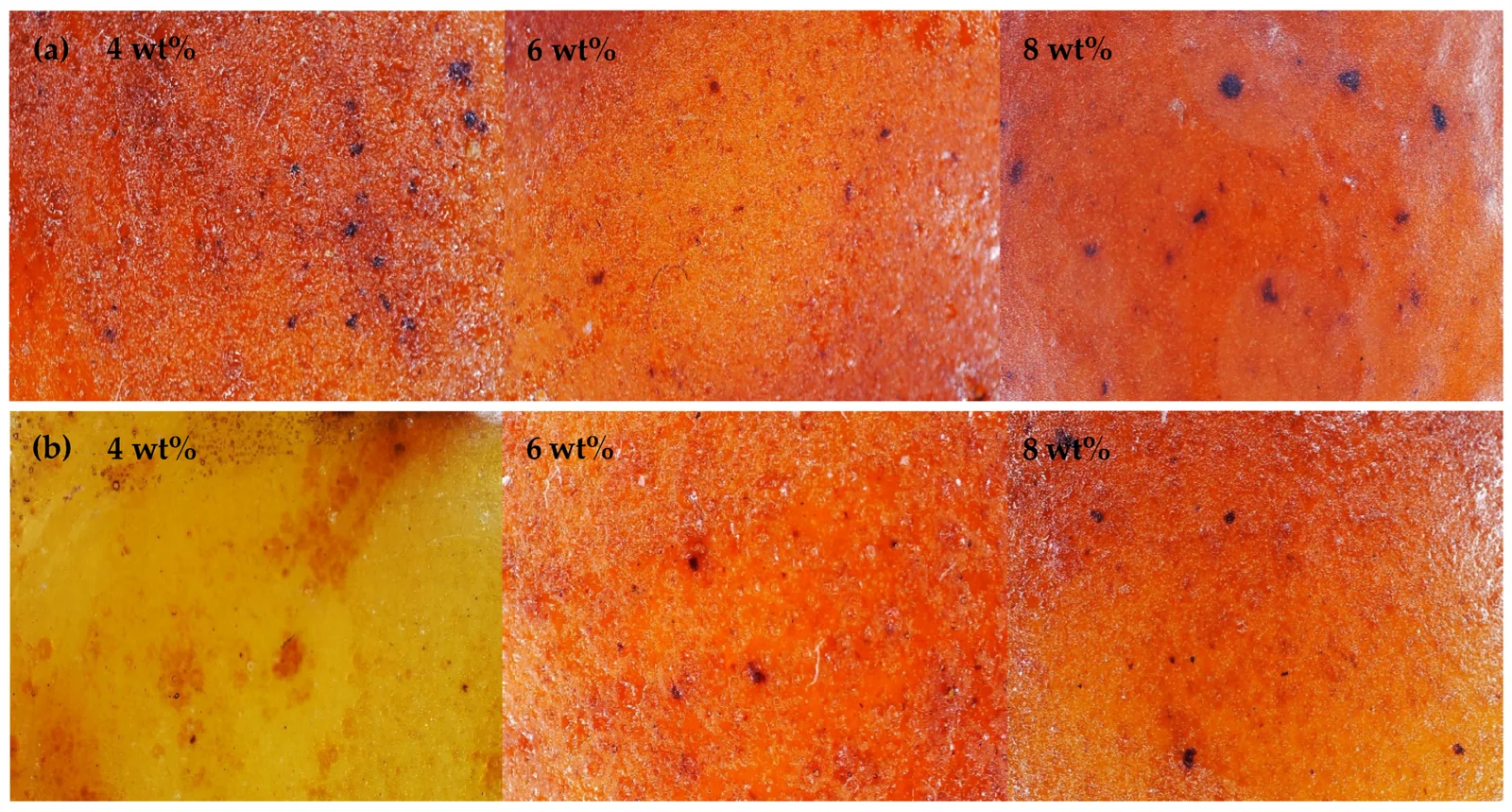

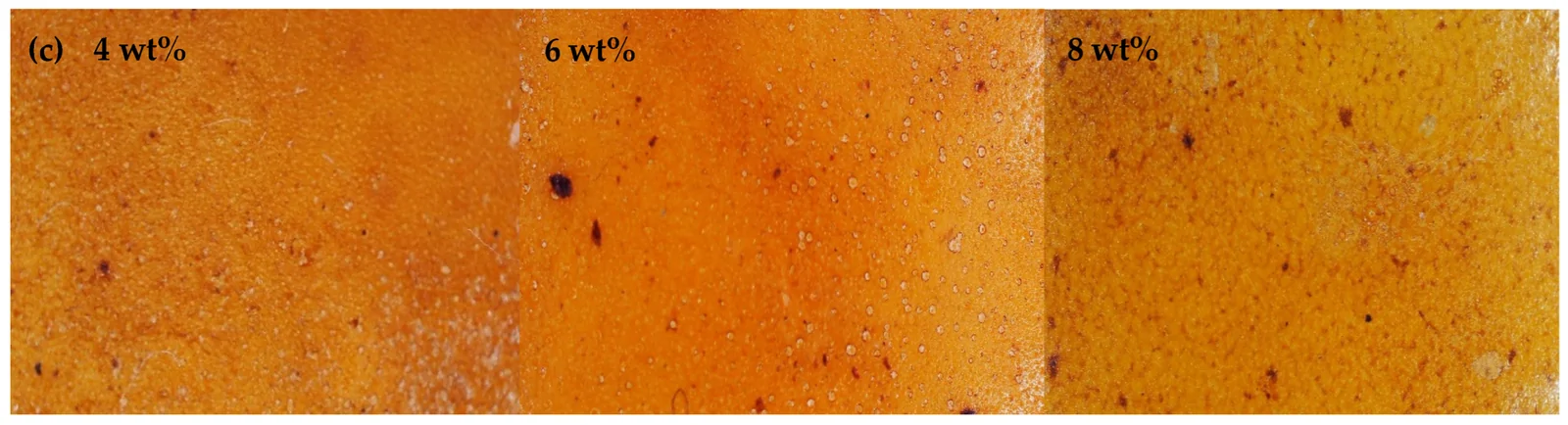
Optical micrographs of the surface of developed biobased films with *Fumaria officinalis* extract (FOE) and propolis: (**a**) CSL-FOE (4–8 wt%), (**b**) CSEL-FOE (4–8 wt%), and (**c**) CSDEA-FOE (4–8 wt%); CS—chitosan, L—limonene, EL—ethyl lactate and DEA—diethyl adipate.

Optical microscopy was performed on the developed films containing FOE and propolis to evaluate their surface morphology and microstructural features, which are important for understanding the material homogeneity and potential film performance (Figure 3). Optical micrographs of the cross-section of developed biobased films with FOEand propolis are presented in the Supplementary Materials (Figure S5). The optical microscopy images of the plain film are presented in the Supplementary Materials as well (Figure S6).

Optical microscopy of the CS-based film incorporating propolis and FOE reveals a relatively heterogeneous microstructure characterized by regions of varying optical density. The film surface appears predominantly continuous, indicating successful film formation; however, darker and lighter domains are consistent with compositional heterogeneity and may indicate partial phase separation between the CS matrix and the incorporated bioactive compounds. Localized aggregates and irregularly distributed domains are observed, which may be attributed to the presence and interaction of propolis and plant-derived polyphenols within the polymer network, as previously reported for bioactives in polymers [49,50,51,52]. These features may be associated with intermolecular interactions and differences in compatibility between hydrophilic CS and more hydrophobic bioactive constituents [49,50,51,53]. In addition, small bubble-like or void structures are observed, which may originate from solvent evaporation during film casting or from entrapped air.

Optical micrographs show changes in surface morphology depending on additive type and extract concentration. Limonene-containing films exhibit dark inclusions and a granular, heterogeneous surface even at low extract concentrations, with these features being consistent with possible microphase separation (Figure 3a). With increasing extract content, aggregation and surface irregularities persist or intensify, consistent with reported phase separation and pore formation in plant bioactives-loaded CS systems [25,54,55]. These observations are consistent with previous reports showing increased heterogeneity, porosity, and aggregation in CS systems containing essential oils or limonene-based components [25,54,55]. EL-based films show a more homogeneous and optically uniform surface than the limonene system, particularly at 4 wt%, with only a slight increase in defects at higher concentrations (Figure 3b), consistent with better dispersion and greater morphological uniformity. The improved optical homogeneity observed in EL-containing films may be associated with differences in polarity and intermolecular interactions between EL and the hydrophilic CS matrix [6,56]. However, these factors represent possible explanations for the observed morphology rather than mechanisms directly demonstrated by optical microscopy. DEA systems exhibit intermediate surface morphology, with a gradual increase in visible microdefects and surface heterogeneity at higher extract loadings, while remaining more morphologically uniform than the corresponding limonene-containing films (Figure 3c). The comparatively improved morphology observed for DEA-containing films may be associated with differences in polarity and plasticizing behavior, which could contribute to the observed morphological uniformity. Although reports specifically focused on DEA-plasticized CS films remain limited, similar morphology-stabilization effects have been reported for compatible plasticizers in CS-based systems [6]. Hence, increasing extract concentration was associated with reduced surface homogeneity, although the magnitude of this change depended on the matrix modifier. Among the investigated surfaces, EL-containing films appeared the most optically uniform, DEA-containing films showed an intermediate morphology, and limonene-containing films displayed the greatest surface heterogeneity.

Cross-sectional images showed clear differences among the formulations (Figure S5). CSL-FOE films remained relatively compact, with a slight increase in heterogeneity at higher FOE concentrations. CSEL-FOE exhibited greater internal non-uniformity, layering, and porosity, particularly at 8 wt% FOE, whereas CSDEA-FOE showed the most compact and homogeneous cross-section with fewer visible defects. Thus, the trends observed at the film surface and in the cross-section were not identical: EL-containing films showed the most uniform surface morphology, whereas DEA-containing films exhibited the most compact internal structure. Surface and cross-sectional morphologies showed related but not identical trends. EL produced the most uniform surface, whereas DEA exhibited the most compact internal structure. Limonene-containing films showed the greatest overall heterogeneity. These differences may reflect the distinct physicochemical behavior of the additives during film formation and solvent evaporation [25,50,55,57,58,59]; however, the proposed effects of polarity, compatibility, and phase separation cannot be directly established from microscopy alone. Although the surface morphology of DEA-based films is not as uniform as in EL systems, the cross-sectional micrographs indicate enhanced bulk compatibility and reduced phase discontinuities. The pronounced heterogeneity observed in limonene-containing films is consistent with the lower morphological uniformity of these formulations. The hydrophobic character of limonene and its compatibility with the hydrophilic CS matrix may contribute to this behavior [25,50,55]; however, microphase separation and aggregation should be considered possible mechanisms rather than direct conclusions from the microscopy observations.

The blank films also showed additive-dependent differences in both surface and cross-sectional morphology (Figure S6). EL produced the most homogeneous and compact structure, while limonene-containing films showed greater surface heterogeneity, dark inclusions, and a less compact cross-section. DEA displayed an intermediate morphology with moderate surface uniformity and relatively compact internal structure. These findings indicate that the additive type influences the baseline morphology of the CS matrix and may affect its subsequent response to FOE and propolis incorporation.

### 3.4. Field Emission Scanning Electron Microscopy of Developed Films

**Figure 4:**
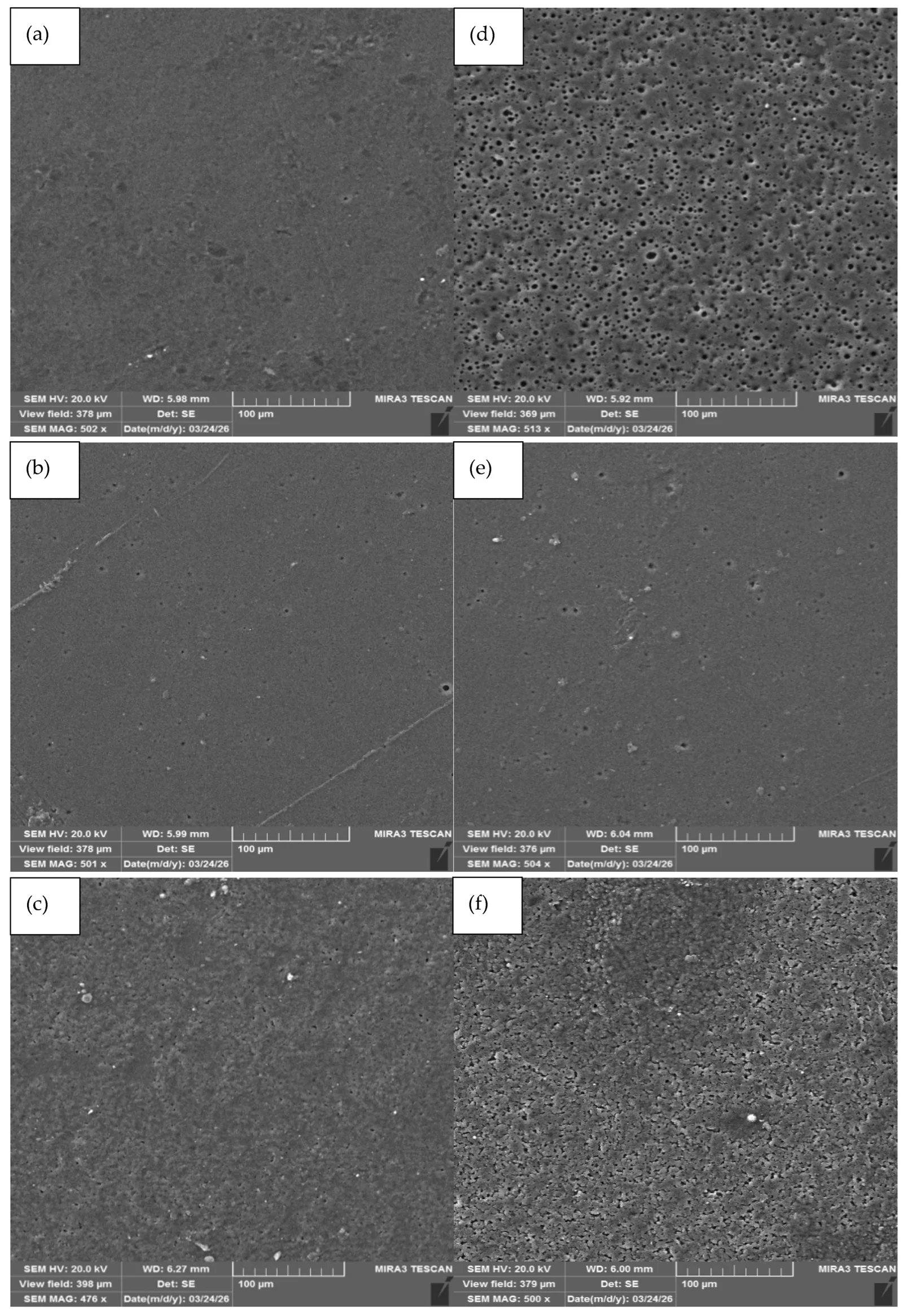
Images of field emission scanning electron microscopy analysis of surface of developed biobased films with *Fumaria officinalis* extract (FOE) and propolis: (**a**) CSL-FOE (4 wt%), (**b**) CSEL-FOE (4 wt%), (**c**) CSDEA-FOE (4 wt%), (**d**) CSL-FOE (6 wt%), (**e**) CSEL-FOE (6 wt%), and (**f**) CSDEA-FOE (6 wt%); CS—chitosan, L—limonene, EL—ethyl lactate and DEA—diethyl adipate, 500× magnification.

**Figure 5:**
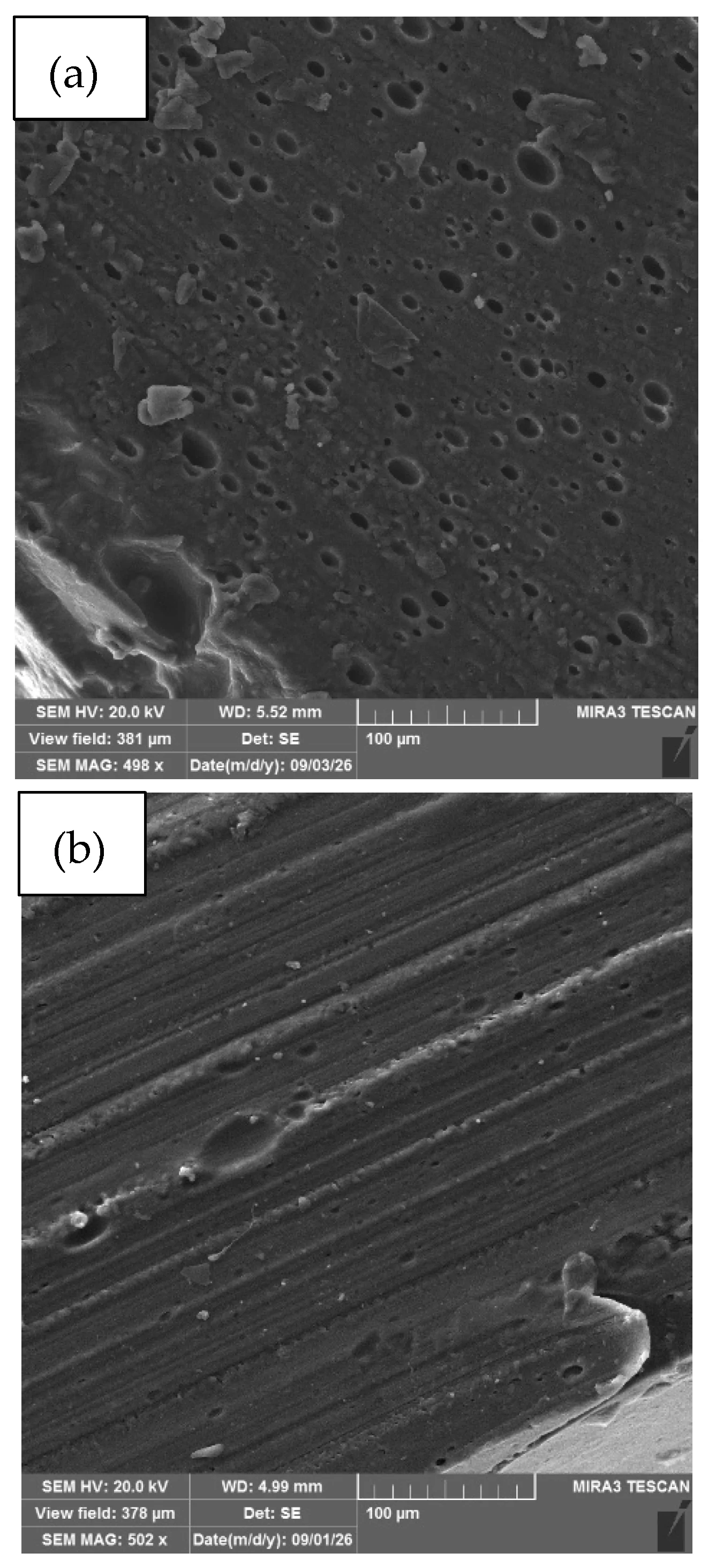

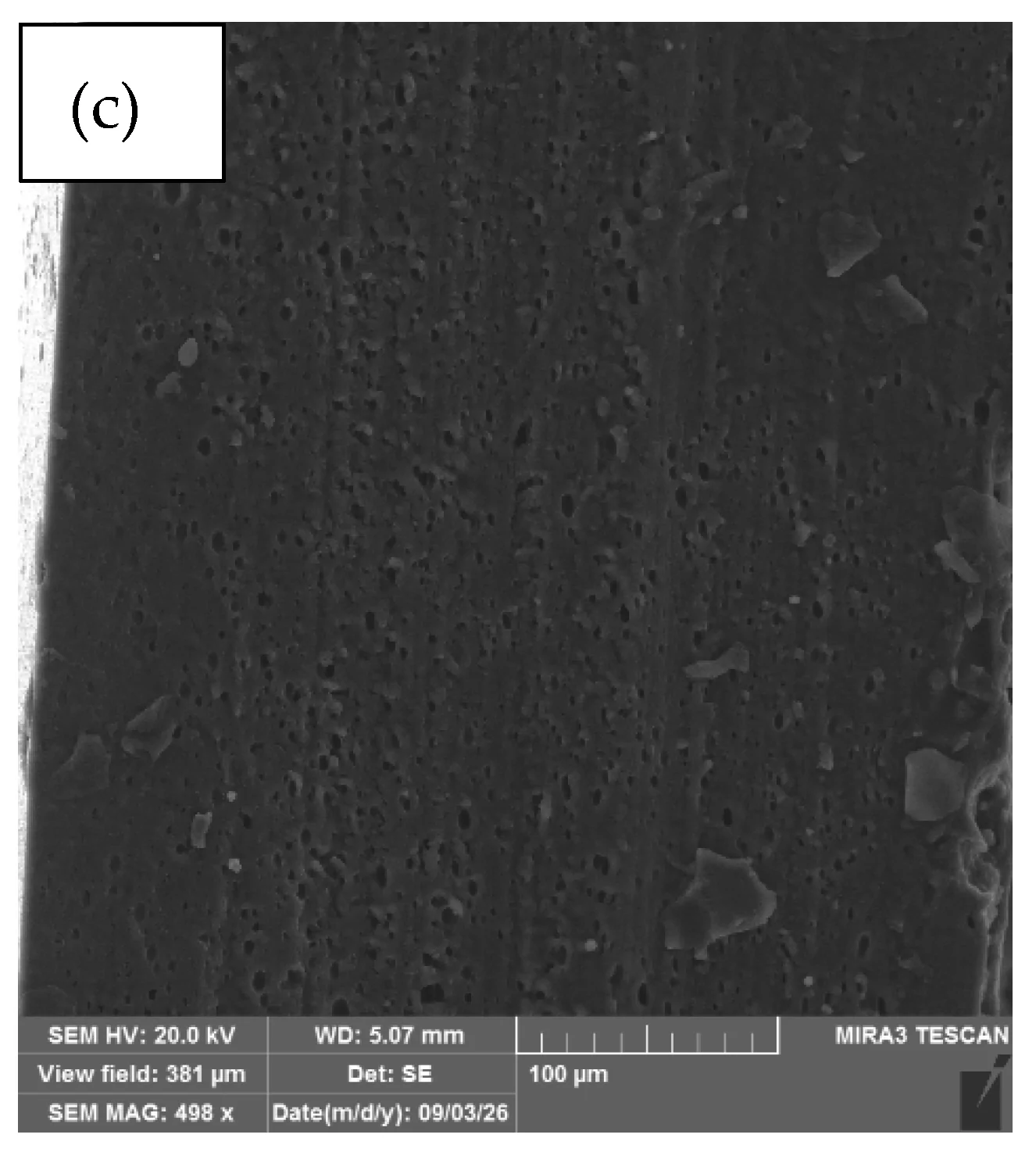
Images of field emission scanning electron microscopy analysis of cross-section of developed biobased films with *Fumaria officinalis* extract (FOE) and propolis: (**a**) CSL-FOE (6 wt%), (**b**) CSEL-FOE (6 wt%), and (**c**) CSDEA-FOE (6 wt%); CS—chitosan, L—limonene, EL—ethyl lactate and DEA—diethyl adipate, 500× magnification.

FESEM images (Figure 4a–f) illustrate the surface morphology of CS-based films incorporated with FOE (at 4 or 6 wt%) and purified propolis, obtained using different additives (limonene, EL, and DEA) at 500× magnification. The cross-sectional morphology of the developed CS-based films containing FOE and propolis was examined by FESEM, and the corresponding images are presented in Figure 5. Additional FESEM images of plain films at 200× magnification (surface) and 500× magnification (cross-section), and extract/propolis-loaded films at 1000× magnification are provided in Supplementary Materials (Figures S7 and S8, respectively).

At a lower FOE concentration (4 wt%), notable differences in surface structure were observed depending on the additive used. The CSL-FOE (4 wt%) film (Figure 4a) exhibited a heterogeneous and irregular surface with visible aggregates and dispersed particles, showing pronounced surface heterogeneity, with visible aggregates and dispersed particles, consistent with non-uniform distribution of the formulation components. This morphology is consistent with non-uniform distribution of bioactive-rich domains within the polymer matrix and may indicate localized phase separation. In contrast, the CSEL-FOE (4 wt%) film (Figure 4b) exhibited a more uniform and compact structure, consistent with greater morphological uniformity. Similarly, the CSDEA-FOE (4 wt%) film (Figure 4c) has a relatively smooth, continuous surface with fewer irregularities than the limonene-based system, showing a relatively smooth and continuous surface with fewer irregularities than the limonene-based system, indicating greater morphological uniformity. Increasing the FOE content to 6 wt% resulted in more pronounced morphological changes. The CSL-FOE (6 wt%) film (Figure 4d) displayed a highly porous structure with numerous microvoids and a non-uniform surface, displayed a highly porous structure with numerous microvoids and a non-uniform surface, indicating increased porosity and morphological heterogeneity at this FOE concentration. Possible contributors to the observed porosity include evaporation of volatile components and aggregation of extract constituents during film formation. On the other hand, the CSEL-FOE (6 wt%) film (Figure 4e) maintained a relatively homogeneous, compact morphology with minimal defects, showing that the relatively homogeneous and compact morphology was retained at the higher FOE concentration. The CSDEA-FOE (6 wt%) film (Figure 4f) also exhibited a fairly smooth surface with only occasional micropores and no evident phase separation, consistent with relatively good structural integrity and low defect density.

FESEM micrographs revealed distinct differences in the cross-sectional morphology of the developed CS-based films depending on the formulation component (Figure 5), indicating that the incorporation of FOE and the type of additive system influenced the internal structure of the CS-based matrix. The CSL-FOE film (Figure 5a) exhibited a relatively heterogeneous and clearly porous morphology, with numerous approximately rounded cavities distributed throughout the matrix, suggesting the formation of voids during film drying and solvent evaporation. In contrast, the CSEL-FOE film (Figure 5b) displayed a more compact and oriented structure, characterized by pronounced longitudinal grooves and fewer clearly defined pores, which may indicate differences in polymer chain organization and phase structure associated with ethyl lactate. The CSDEA-FOE film (Figure 5c) appeared comparatively homogeneous and compact, with a fine and relatively uniform distribution of small pores throughout the cross-section. These morphological differences suggest that limonene, EL, and DEA can differently affect the arrangement and interactions within the CS matrix, as well as the evaporation/drying process and, consequently, the resulting film microstructure. Namely, the observed pores, voids, and discontinuities may be associated with differences in component compatibility, solvent removal, and possible local phase separation during film formation. The SEM observations confirm that the composition of the formulation plays an important role in determining the internal morphology of the FOE-containing biobased films. However, such mechanisms should be considered cautiously and interpreted in conjunction with the physicochemical and mechanical properties of the films.

As can be concluded, FESEM analysis confirms that the type of additive plays a crucial role in determining film morphology. Namely, EL- and DEA-containing films exhibited greater structural uniformity than the limonene-containing formulations, particularly at higher FOE concentrations. Additionally, increasing extract content enhances porosity and heterogeneity, particularly in less-compatible systems. The observed differences in surface morphology may be related to the intrinsic physicochemical properties of the additives and their interactions with CS, propolis, and FOE. Due to abundant –OH and –NH_2_ groups, CS is highly polar and forms extensive hydrogen bonding, making additive compatibility strongly dependent on polarity and hydrogen-bonding ability [6,7]. The poor morphology of CSL systems, particularly at 6 wt% FOE, is linked to limonene, a nonpolar, hydrophobic terpene with minimal hydrogen-bonding capacity. Its hydrophobic character and limited hydrogen-bonding capacity may contribute to less favorable interactions with the hydrophilic CS matrix, which could be associated with the greater heterogeneity, aggregation, and microvoid formation observed in the limonene-containing films. Its volatility further contributes to pore development during solvent evaporation, while the presence of FOE and propolis enhances multiphase incompatibility [60]. In contrast, EL and DEA exhibit better compatibility. The relatively uniform morphology observed for EL-containing films may be associated with differences in polarity and intermolecular interactions between EL and the CS-rich matrix. These interactions could contribute to improved dispersion and matrix cohesion [7,21,61], although they were not directly assessed by FESEM. DEA-containing films exhibited relatively smooth and continuous morphologies with limited visible porosity, even at higher FOE content. This behavior may be associated with the physicochemical characteristics and plasticizing behavior of DEA [22,23]. Nevertheless, changes in polymer chain mobility or intermolecular interactions were not directly assessed in the present FESEM analysis. Similar behavior has been reported in polymer films plasticized with DEA, where increased flexibility and improved structural properties were observed [22]. The FESEM images show that surface morphology strongly depends on additive type and FOE content. EL- and DEA-containing films exhibit more uniform surfaces than the corresponding limonene-containing formulations, particularly at higher FOE loading. These differences may be associated with differences in polarity and physicochemical compatibility between the ester-based additives and the CS-rich matrix, whereas the hydrophobic and volatile nature of limonene may contribute to the greater surface heterogeneity and pore formation observed in these formulations [6,7,23,60,61]. Increasing FOE content further modifies the film surface, with the most pronounced increase in porosity observed in the limonene-containing formulation. Thus, the observed differences in film morphology are associated with additive type and FOE loading, while differences in polarity and intermolecular interactions may represent contributing factors.

### 3.5. Atomic Force Microscopy of Developed Films

AFM analysis was used to evaluate the surface nano-topography of CS-based films containing FOE and propolis before and after propolis purification. The data are presented in Figure S9 (Supplementary Materials). The film with non-purified propolis exhibited a relatively continuous but moderately heterogeneous surface. For the scanned area of 1.00 × 1.00 μm, the roughness parameters were Ra = 5.535 nm and Rq = 7.018 nm, while the maximum height difference was Rz = 40.653 nm. These values show a moderately heterogeneous surface with local protrusions and depressions. Such features may be associated with differences in the distribution of propolis-derived components within the CS matrix. Similar changes in surface morphology have been reported after incorporation of propolis into CS-based films, where the bioactive additive altered the homogeneity and roughness of the polymer surface [62,63]. After propolis purification, the AFM image recorded over a 500 × 500 nm area showed a developed nano-topography with a total height range of 105.83 nm. The projected area was 0.250000 μm^2^, whereas the real surface area was 0.269259 μm^2^, indicating nanoscale surface relief. Because the two samples were recorded over different scan areas, their roughness and height parameters should not be quantitatively compared directly. Nevertheless, the AFM images indicate a qualitative change in surface organization after propolis purification, which may be associated with changes in the composition and distribution of propolis-derived components following the purification. These observations are consistent with the optical microscopy results showing surface heterogeneity associated with the incorporation of FOE and propolis [62,63]. AFM images of the other samples are not presented due to technical issues during scanning, specifically local laser-induced heating that caused plasticizer/additive migration and compromised the reliability of the recorded data. Thus, these AFM observations are qualitatively consistent with the optical microscopy results, which showed differences in surface heterogeneity among the formulations. Comparable morphological changes, including altered surface uniformity after the propolis or other active compounds’ addition, have also been observed in CS films [62,64]. Qualitatively, the purified-propolis film exhibited a more developed and spatially organized nanoscale topography, although this observation should be interpreted cautiously given the limited AFM dataset. Therefore, the AFM results provide qualitative evidence that propolis purification may be associated with changes in the nanoscale surface organization of CS-based films.

### 3.6. Dynamic Mechanical Analysis of Developed Films

**Figure 6:**
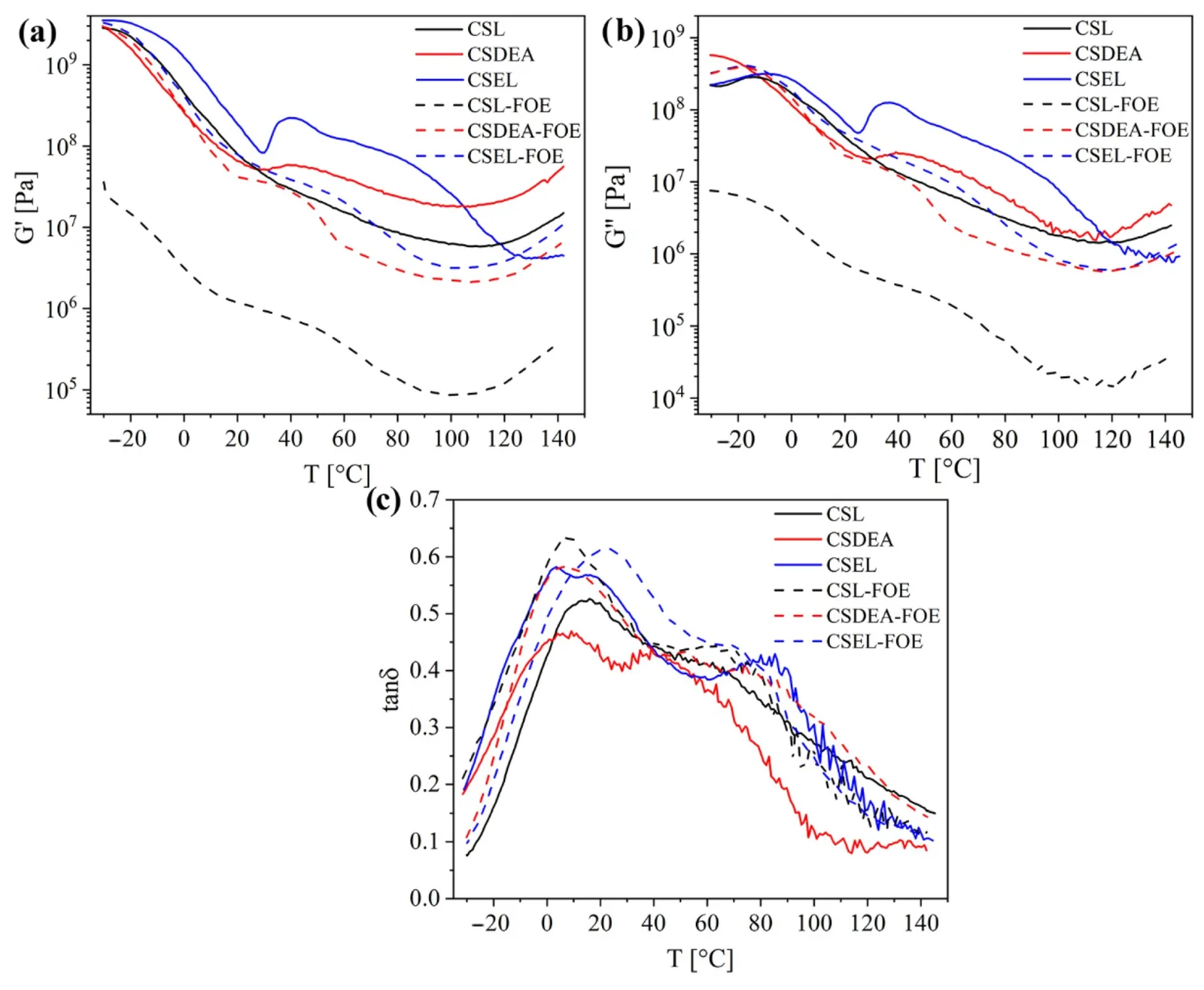
Temperature dependence of (**a**) storage modulus, (**b**) loss modulus, and (**c**) damping factor for chitosan-based polymer blends; CS—chitosan, L—limonene, EL—ethyl lactate, DEA—diethyl adipate and FOE—*Fumaria officinalis* extract. FOE-containing curves correspond to films containing 6 wt% FOE and propolis.

DMA was used to evaluate the temperature-dependent viscoelastic response of the CS-based films by monitoring the storage modulus (*G*′), loss modulus (*G*″), and damping factor (*tanδ*) from −30 to +145 °C (Figure 6). Control samples and films containing propolis and 6 wt% FOE were analyzed, with the latter selected as a representative formulation because 6 wt% FOE provided a suitable compromise between enhanced antioxidant functionality and retention of mechanical integrity, whereas the higher 8 wt% loading produced a more pronounced reduction in tensile strength. All samples showed a decrease in *G*′ and *G*″ with increasing temperature, reflecting thermally induced softening and increased polymer-chain mobility [65,66]. However, the magnitude of this response differed among the additive systems.

The CSL blend exhibits high *G*′ up to about 30 °C, after which it declines at higher temperatures (Figure 6a). Its relatively high *G*′ at low temperatures indicates that limonene maintains a comparatively rigid network, likely because of its limited plasticizing efficiency. *G*″ follows a broadly similar temperature dependence, also decreasing markedly with increasing temperature. As temperature increases from approximately −25 °C to 30 °C, both moduli decrease by nearly two orders of magnitude, reflecting gradual softening of the CS matrix and increased segmental mobility of the CS chains. Between about 30 °C and 120 °C, the decrease in *G*′ and *G*″ becomes more gradual, suggesting that the polymer network retains part of its elastic and viscous resistance over this temperature interval. The slight increase in both moduli above approximately 120 °C may be associated with dehydration-induced stiffening and/or thermally induced rearrangement of the polymer network. The high volatility and hydrophobicity of limonene may additionally contribute to the observed behavior [24]. Incorporation of FOE and propolis produces a pronounced decrease in both moduli. The CSL-FOE sample displays the lowest moduli throughout the investigated temperature range, reaching values close to 10^5^ Pa at around 100–110 °C and 10^4^ Pa at around 120 °C for *G*′ and *G*″, respectively (Figure 6a,b). Incorporation of FOE and propolis markedly reduced both moduli, with CSL-FOE showing the lowest values throughout the tested range, indicating the strongest reduction in viscoelastic resistance among the formulations. The slight increase in both moduli above approximately 120 °C is similar to that observed for the other samples and is likely associated with thermal dehydration and temporary network rearrangement.

The CSDEA blend retains relatively high *G*′ and *G*″ values over most of the investigated temperature range. CSDEA retained relatively high *G*′ and *G*″ values over most of the temperatures investigated. Incorporation of FOE and propolis reduced both moduli, although the decrease was less pronounced than in CSL-FOE. The approximately parallel curves indicate that FOE incorporation did not substantially alter the temperature dependence of the viscoelastic response.

The CSEL blend exhibits the highest *G*′ and *G*″ values over a substantial part of the temperature range investigated, indicating the greatest resistance to elastic and viscous deformation among the control formulations. An interesting feature is observed at about 30 °C, where the module initially decreases strongly and reaches a minimum, followed by a pronounced increase around 35–45 °C. This behavior differs from simple monotonic thermal softening and may indicate temperature-induced structural rearrangement or relaxation/reorganization of the polymer-plasticizer network. After this maximum, the module decreases progressively. Therefore, the EL-containing system appears to have a particularly temperature-sensitive microstructure. The reduction in the moduli after incorporation of FOE and propolis is considerably smaller for CSEL than for CSL, indicating that the EL-containing network better preserves its viscoelastic response. The *G*″ of CSEL-FOE remains comparatively close to that of the corresponding CSEL sample over most of the temperature range, suggesting that incorporation of the bioactive components causes only a moderate modification of the viscous response. The relatively small change in *G*″ suggests that incorporation of FOE and propolis had a moderate effect on the viscous component of the response.

Comparison of *G*′ and *G*″ shows that incorporation of FOE and propolis produced the greatest reduction in viscoelastic resistance in the limonene-containing system, while CSEL retained the highest moduli and CSDEA showed an intermediate response. These results indicate that the additive type strongly influences the extent to which the CS-based network is affected by bioactive incorporation.

The *tanδ* curves of all CS blends exhibit a broad low-temperature maximum in the region between approximately 0 °C and 40 °C, followed by a gradual decrease as temperature increases (Figure 6c). The broad nature of the relaxation region indicates that the process occurs over a wide temperature range rather than through a sharp transition, which is typical of heterogeneous, physically crosslinked biopolymer systems. Some samples also exhibit a secondary relaxation feature extending toward approximately 90–110 °C. The relaxation behavior observed in the region between approximately 30 °C and 50 °C may partially originate from CS and can be associated with β-relaxation involving local lateral motions of polymer segments and the influence of water evaporation from the polymer film [67,68]. A higher-temperature relaxation feature around 103 °C has also been associated with the presence of acetamide groups anchored at the C-2 position in the CS backbone (Figure 6c) [69]. This feature has also been related to the glass transition of hydrated CS [70].

The CSL blend shows intermediate damping behavior, with a maximum *tanδ* of approximately 0.50–0.52 in the temperature interval between 10 °C and 15 °C. In contrast, the CSL-FOE sample shows the highest *tanδ* values among the investigated formulations, reaching a maximum of approximately 0.63 between 5 °C and 10 °C. This high damping factor indicates that incorporation of FOE and propolis promotes greater molecular mobility and enhances the viscous contribution relative to the elastic response. This increase in *tanδ* is consistent with a greater relative contribution of viscous dissipation after incorporation of FOE and propolis. The CSDEA sample exhibits the lowest relative damping over much of the investigated temperature range, indicating a more predominantly elastic response. After reaching a broad maximum of approximately 0.45, *tanδ* decreases continuously above approximately 40 °C and reaches values below 0.15 at temperatures above 100 °C. CSDEA-FOE exhibits higher *tanδ* values than CSDEA over most of the investigated range, with a broad low-temperature maximum, indicating an increased relative contribution of viscous dissipation after incorporation of FOE and propolis.

CSEL displays a broad *tanδ* maximum of approximately 0.57–0.58 at around 10–15 °C, followed by a broad relaxation region extending toward higher temperatures and a noticeable secondary shoulder near 100–110 °C. This behavior is consistent with a complex temperature-dependent relaxation response of the CSEL system. CSEL-FOE exhibits a slightly higher *tanδ* maximum of approximately 0.60, shifted toward higher temperatures, approximately 20–30 °C. This shift and broadening suggest modification of the relaxation behavior after incorporation of FOE and propolis.

The position and magnitude of these maxima depend strongly on the additive type. Among the FOE/propolis-free samples, CSEL exhibits the highest damping maximum, whereas CSDEA shows the lowest relative damping. Incorporation of FOE and propolis increases the maximum *tanδ* values of all formulations, with the effect being most pronounced for CSL-FOE, which simultaneously exhibits the lowest storage modulus. The greater reduction in *G*′ relative to *G*″ results in an increased *G*″/*G*′ ratio and a more dissipative viscoelastic response. Although *G*″ also decreases after incorporation of FOE and propolis, *G*′ is reduced to a greater extent, resulting in an increased *G*″/*G*′ ratio. The *tanδ* results show that incorporation of FOE and propolis shifts the films toward a relatively more dissipative viscoelastic response, with the magnitude of this effect strongly dependent on the additive type.

### 3.7. Thickness and Tensile Strength of Developed Films

The film thickness ranged from 0.270 to 0.376 mm (Table 2). Within each film series, a modest increase in thickness was observed with increasing FOE content: from 0.323 to 0.352 mm for CSDEA films, from 0.270 to 0.284 mm for CSEL films, and from 0.330 to 0.376 mm for CSL films. These quantitative thickness values are reported because film geometry may contribute to the interpretation of mechanical, swelling, release, and diffusion-related behavior. Although film thickness increased moderately with increasing FOE content, the concomitant decrease in stress at break should not be attributed directly to thickness, since tensile stress was calculated using the measured specimen cross-sectional area. Rather, the increase in thickness appears to accompany formulation-dependent changes in matrix organization and compactness. The results of the two-way ANOVA for the effects of additive type and FOE concentration on thickness, break stress, and break strain are presented in Tables S4, S5, and S6, respectively. Additive type showed a statistically significant effect on thickness, while FOE concentration and the interaction between the two factors did not significantly affect the mentioned parameter (Table S4). Both additive type and FOE concentration significantly affected break stress and break strain, and a significant interaction between the two factors was observed for break stress (*p* < 0.0001) (Tables S5 and S6).

In the control films without FOE and propolis, CSEL shows the highest stress at break (~53.58 N/mm^2^), indicating the highest resistance to tensile failure among the control formulations. CSDEA exhibits a somewhat lower value (~46.12 N/mm^2^), whereas CSL shows the lowest initial tensile resistance (~34.19 N/mm^2^). Thus, the stress-at-break values follow the order CSEL > CSDEA > CSL. Previous studies have similarly shown that essential oils and plasticizer type can substantially influence the mechanical properties of CS-based films [53,54,60,71]. Additionally, several studies reported that the type of plasticizer significantly changes the mechanical properties of CS-based films [61,72].

Within the propolis-containing formulations, increasing FOE concentration causes a progressive decrease in stress at break. For CSEL-FOE, stress at break decreases from ~44.42 N/mm^2^ at 4 wt% FOE to ~22.80 N/mm^2^ at 8 wt% FOE. A similar trend is observed for CSDEA-FOE, decreasing from ~41.93 to ~14.25 N/mm^2^. The most pronounced loss of tensile resistance at higher FOE contents is observed for CSL-FOE, with stress at break decreasing to ~14.78 N/mm^2^ at 6 wt% and ~10.98 N/mm^2^ at 8 wt% FOE. These results demonstrate that increasing FOE concentration represents a major formulation trade-off: although higher extract loading increases the amount of incorporated bioactive material, it substantially compromises the tensile resistance of the films. The decrease may be associated with changes in intermolecular interactions and increased structural heterogeneity at greater FOE contents [54,60,73], although these mechanisms cannot be directly confirmed from tensile data alone. In particular, the more porous and heterogeneous cross-section of CSL-FOE (6 wt%) is consistent with its markedly lower stress at break, whereas the more continuous CSEL-FOE and especially the more compact CSDEA-FOE cross-sections correspond to substantially higher tensile strength. It is also possible that phase separation and the formation of a heterogeneous structure occur, further contributing to material weakening [71,73].

CSEL-FOE retains the highest tensile resistance at higher FOE concentrations, despite the overall decrease observed in all formulations. At 6 wt% FOE, CSEL-FOE and CSDEA-FOE exhibit statistically comparable stress-at-break values, as indicated by the same Tukey grouping. At 8 wt% FOE, CSEL-FOE retains a substantially higher stress at break than the corresponding CSDEA-FOE and CSL-FOE formulations. Thus, EL appears to provide a comparatively favorable matrix environment for retaining tensile resistance after incorporation of the bioactive components. Nevertheless, this advantage should be considered together with the pronounced decrease in stress at break observed with increasing FOE concentration. Namely, the CSEL-FOE system decreases from ~44.42 N/mm^2^ at 4 wt% FOE to ~22.80 N/mm^2^ at 8 wt% FOE. Therefore, EL cannot be considered universally optimal across all FOE concentrations; rather, its performance reflects a balance between bioactive loading and mechanical integrity. The comparatively higher tensile resistance of CSEL-FOE at elevated FOE concentrations may be associated with differences in component interactions and morphological organization, although these proposed mechanisms cannot be directly established from tensile measurements alone.

Plain films show different strain-at-break values, with the highest value recorded for CSEL (~14.05%), followed by CSL (~10.64%), while CSDEA exhibits the lowest value (~8.10%). In contrast to stress at break, strain at break shows a non-monotonic response to FOE concentration. For CSL-FOE, strain at break increases to ~14.81% at 4 wt% FOE, decreases to ~12.06% at 6 wt%, and increases to ~14.52% again at 8 wt%. An oscillatory trend is also observed for CSEL-FOE, with strain at break decreasing from ~11.77% at 4 wt% FOE to a minimum of ~9.48% at 6 wt%, followed by a slight recovery to ~10.37% at 8 wt%.

A similar non-monotonic response is observed for CSDEA-FOE. The strain-at-break response appears to depend on both matrix composition and FOE concentration. While some CSL and CSDEA formulations show increased deformability after bioactive incorporation, CSEL exhibits a reduction in strain at break. These differences may reflect competing effects of molecular mobility and structural heterogeneity [73]. Similar changes in elongation have been reported for CS films containing propolis [50]. Namely, a reduction in elongation associated with polyphenol-induced structural ordering has also been reported for CS-based films [74]; however, the tensile data alone cannot confirm such a mechanism for the present films. The CSEL-containing formulations therefore show a comparatively favorable mechanical response among the investigated systems, particularly in terms of retaining tensile resistance at higher FOE concentrations. Nevertheless, the substantial reduction in stress at break with increasing FOE content demonstrates that this advantage is accompanied by a clear strength-bioactive-loading trade-off. The CSEL formulation should therefore be regarded as a comparatively favorable compromise under the tested conditions rather than as an unequivocally optimal formulation. Thus, the favorable mechanical behavior of CSEL is reflected not only in its relatively high resistance to tensile failure but also in its ability to maintain a moderate level of deformability after incorporation of FOE and propolis.

The tensile-strength values obtained in the present study are within the range reported for mechanically robust CS films. However, direct comparison should consider differences in CS molecular characteristics, formulation, and conditioning conditions. Magallanes-Vallejo et al. [75] reported a tensile strength of 49.60 ± 1.55 MPa for a high-molecular-weight CS film (310 kDa, DDA 75%), while Alves et al. [76] obtained 32.06 MPa for commercial CS with Mw of 208 kDa and DDA of 77%. Stefanowska et al. [77] reported 46.95 MPa for an unmodified CS film prepared in acetic acid; however, incorporation of ethanolic propolis extract reduced the tensile strength to 15.52 MPa, highlighting the challenge of maintaining mechanical integrity after incorporation of bioactive components into CS films. This challenge is also emphasized in the recent review by Liu et al. [78].

A different strategy for improving CS-film strength was demonstrated by Xu et al. [79], who increased tensile strength from 37.6–60.5 MPa before neutralization to approximately 112–119 MPa after alkaline neutralization. However, this increase in strength was accompanied by reduced elongation and required additional post-processing steps. In contrast, the objective of the present formulation was not to maximize tensile strength alone, but to achieve a balanced mechanical response considering both tensile resistance and deformability, as reflected by stress at break and strain at break, while incorporating propolis and FOE through a comparatively simple film-forming procedure. The present approach achieved a balanced mechanical response without additional post-neutralization or controlled-humidity conditioning.

Although the incorporation of FOE and propolis may adversely affect the mechanical integrity of the films, particularly at higher FOE loadings, their antioxidant activity and potential bioactive functionality provide an important functional benefit, indicating that their incorporation should be considered as a balance between enhanced bioactivity and preservation of adequate mechanical performance.

### 3.8. Water Contact Angle Data of Developed Films

Water contact angle measurements were performed to evaluate the surface wettability of CS-based films containing FOE and propolis. Since the contact angle reflects the hydrophilic or hydrophobic nature of the film surface, these measurements provide important information about the influence of incorporated bioactive components and additives on the film surface properties. The values obtained are presented in Table 3. The two-way ANOVA revealed that additive type, FOE concentration, and their interaction significantly affected the water contact angle (*p* < 0.05) (Table S7).

The water contact angle values of the developed biobased films indicate differences in surface wettability depending on the film matrix type and FOE concentration. However, all measured contact angles remained below 90°. In the case of CSL-FOE films, a gradual increase in the contact angle was observed with increasing FOE concentration, from ~56.4° for the film containing 4 wt% to ~60.3° for the film containing 8 wt%, indicating that the extract addition contributes to an increase in surface hydrophobicity. A similar trend was observed for CSEL-FOE films, although the change was not strictly monotonic, with contact angles of ~60.7°, ~59.5°, and ~62.5° for 4, 6, and 8 wt% FOE, respectively. This trend may reflect changes in surface composition and the relative exposure of hydrophilic and hydrophobic groups. This trend is consistent with a previous report [53]. The most pronounced hydrophobicity was observed in CSDEA-FOE films with 8 wt% FOE (~69.1°), indicating a significantly modified surface structure and reduced interaction with water. At lower FOE concentrations, the contact angles remained lower (~58.7° and ~60.2°), suggesting a concentration-dependent modification of surface wettability. The increase in contact angle with FOE concentration in CSDEA films further indicates a concentration-dependent change in surface wettability. The results indicate that both matrix composition and FOE concentration influence the surface wettability of the films. The hydrophilic-hydrophobic balance may be relevant for potential dermal applications, as it can influence moisture retention and skin adhesion.

### 3.9. Swelling Capacity of Developed Films

The swelling behavior of the developed CS-based films containing FOE and propolis was investigated in PBS solution to evaluate their water absorption capacity over time, and the obtained results are presented in Table 4. Results of two-way ANOVA for the effect of additive type and FOE concentration on SR at different incubation times are presented in Table S8. The results indicate that the composition of biobased films, as well as the concentration of the added extract, significantly affects the swelling behavior in PBS buffer (pH 7.0). The films exhibited SRs ranging from ~3.88 to ~9.61, indicating a high-water uptake capacity of the polymer matrix. High swelling values have already been reported for hydrophilic polysaccharide-based systems such as CS/pullulan matrices [80,81].

Most formulations show an initial increase or relatively stable swelling during the first 30–60 min, followed by stabilization or a slight decrease. In the CSL-FOE system, 4 wt% FOE showed moderate and relatively stable swelling, whereas 6 wt% resulted in a less stable profile followed by a decrease. However, CSL-FOE (6 wt%) and CSDEA-FOE (6 wt%) disintegrated during prolonged immersion, indicating that aqueous stability also depends strongly on the type of matrix modifier. In contrast, films with 8 wt% FOE showed consistently lower swelling, indicating reduced water uptake at higher extract loading. A similar trend is observed in the CSEL-FOE system, where films with 4 and 6 wt% show a higher degree of swelling than the 8 wt% formulation, while CSEL-FOE (6 wt%) remains comparatively stable throughout the period investigated. The highest swelling values were observed for CSDEA-FOE films, particularly at 4 wt%, where initial values exceeded 9. Increasing FOE content to 8 wt% reduced swelling, indicating lower water uptake at higher loading. The results show that swelling behavior depended on both the matrix modification and FOE concentration, with 8 wt% FOE showing the lowest water uptake.

### 3.10. In Vitro Released Polyphenols

**Figure 7:**
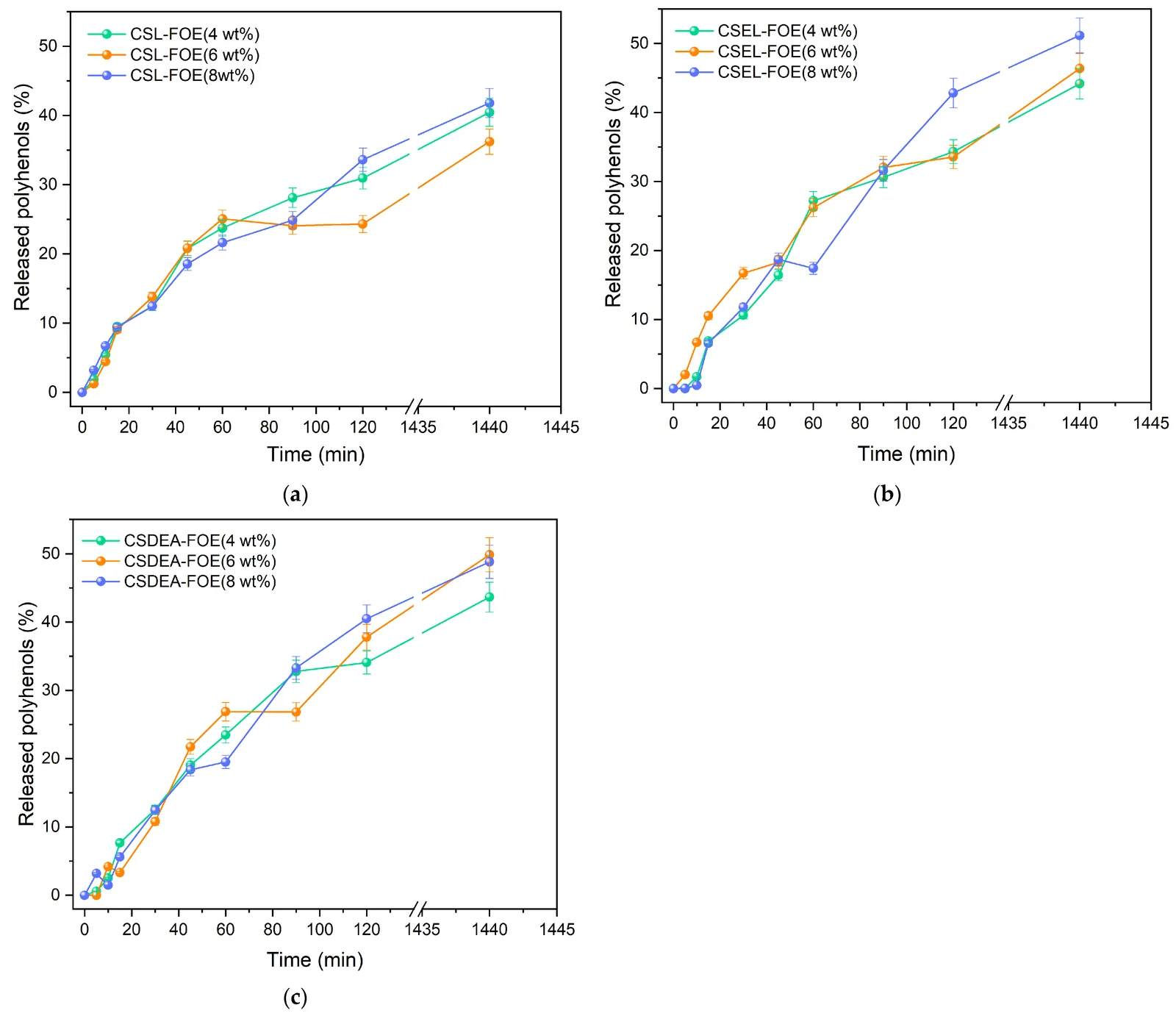
Kinetics of polyphenol release from developed biobased films with *Fumaria officinalis* extract (FOE) and propolis: (**a**) CSL-FOE (4–8 wt%), (**b**) CSEL-FOE (4–8 wt%), and (**c**) CSDEA-FOE (4–8 wt%); CS—chitosan, L—limonene, EL—ethyl lactate and DEA—diethyl adipate; percentage release *versus* time in a Franz diffusion cell (phosphate-buffered saline, pH 7, ambient temperature); the break in the time axis between 120 and 1440 min is used for visualization purposes to improve the readability of the long-term release profile.

FOE contained 117.9 ± 5.4 mg GAE/g dry weight, whereas the total polyphenols of purified propolis were 211.5 ± 6.0 mg GAE/g propolis. These experimentally determined values were used to calculate the initial total polyphenol content of the corresponding films and, consequently, the percentage of released polyphenols. The polyphenol release profiles in the developed biobased films are shown graphically in Figure 7 as percentage release *versus* time in a Franz diffusion cell in PBS medium (pH 7).

Figure 7a illustrates the release kinetics of polyphenols from CSL films incorporating different concentrations of FOE (4–8 wt%) and propolis, at ambient temperature. All formulations exhibit a time-dependent increase in polyphenol release, characterized by an initial slow-release phase (0–15 min), followed by a more pronounced release between 30 and 90 min and continued release up to 1440 min. This profile may reflect the progressive hydration of the film matrix and the increasing availability of polyphenols for release; however, the present release data alone do not allow the specific contribution of molecular diffusion, matrix relaxation, or swelling to be distinguished [82,83,84,85]. However, because the shape of the release curve alone does not allow the relative contributions of molecular diffusion, matrix relaxation, and swelling to be distinguished, kinetic modeling was additionally performed as described below. At early time points (≤15 min), differences among formulations are minimal. From 30 min onward, the 6 and 8 wt% formulations showed slightly higher release values than the 4 wt% formulation [86]. Between 45 and 90 min, CSL-FOE (6 wt%) demonstrates a marginally higher measured release value than the other samples, suggesting that differences in matrix organization and compound availability may contribute to the observed release behavior. At longer times (120–1440 min), the measured cumulative release values were approximately 42%, 40%, and 36% for CSL-FOE 8, 4, and 6 wt%, respectively. This behavior may be related to the greater amount of polyphenols incorporated at 8 wt%, while differences in matrix organization and polymer-polyphenol interactions may also contribute to the observed release profiles [86]. The cumulative release did not vary monotonically with FOE loading, although the 8 wt% formulation showed the highest numerical 24 h release within the CSL series. These differences may reflect formulation-dependent variations in matrix organization and polyphenol availability; however, the specific underlying mechanisms were not directly established. Continued release over the 24 h evaluation period indicates a prolonged release profile under the experimental conditions.

As shown in Figure 7b, the release kinetics of polyphenols from CSEL films with various concentrations of FOE and propolis were monitored under the same Franz diffusion cell conditions. Similar to the CSL systems, all CSEL-FOE formulations exhibit a gradual, time-dependent increase in polyphenol release, with an initial lag phase (0–15 min), followed by an accelerated release between 30 and 120 min, and a sustained release up to 1440 min. However, compared to the limonene-containing films, a higher cumulative release values were measured, suggesting that the presence of EL may influence the physicochemical organization and hydration of the film matrix and consequently affect polyphenol availability for release [87]. At early time points, release remained low for all formulations. From 15 to 45 min, CSEL-FOE (6 wt%) shows slightly higher measured release values than the other formulations, suggesting differences in the availability of polyphenols for release, potentially influenced by polymer–solvent interactions and the hydration state of the matrix [85]. Between 60 and 120 min, this increase may indicate that higher FOE loading, in combination with EL, could modify the organization and hydration of the polymer matrix and consequently influence polyphenol release. At the final point (1440 min), the measured cumulative release values were approximately 51%, 46%, and 44% for CSEL-FOE 8, 6, and 4 wt%, respectively. In the CSEL films, the measured cumulative release values increased numerically with increasing FOE concentration. This trend may reflect differences in the interactions between polyphenols and the CS matrix in the presence of EL, although the strength of these interactions was not directly determined in the present study.

The polyphenol release from CSDEA-FOE films containing 4, 6, and 8 wt% of FOE and propolis, evaluated in a Franz diffusion cell in PBS at ambient temperature, showed that the samples exhibit a time-dependent increase in polyphenol concentration in the acceptor compartment (Figure 7c). The formulations showed sustained release, with profiles differing according to FOE loading. Release was minimal during the first 15 min and increased gradually after 15–30 min. Further, from 30 to 90 min, a more pronounced release phase occurs. The CSDEA-FOE (6 wt%) sample shows a relatively larger numerical increase during this interval compared with the 4 and 8 wt% films. In contrast, the 8 wt% formulation showed a delayed but sharper increase after 60 min, possibly reflecting differences in the organization and retention of extract components within the matrix [86]. At longer times (120–1440 min), all formulations showed progressively higher cumulative release. At the end of the 24 h evaluation period, the measured cumulative release values were approximately 50% for CSDEA-FOE (6 wt%), 48–49% for CSDEA-FOE (8 wt%), and 43–44% for CSDEA-FOE (4 wt%). At higher loading (8 wt%), partial saturation or stronger interactions within the polymer network may slightly limit polyphenol release despite the higher initial content [86]. Across the three matrix modifications, the measured cumulative release values differed among the film systems. Under the present experimental conditions, the CSEL formulations reached cumulative release values of up to approximately 51%, whereas the CSL and CSDEA formulations reached values of approximately 42% and 50%, respectively.

The zero-order and first-order models were applied according to established release-kinetic approaches [87], the Higuchi model according to Higuchi [88], and the Korsmeyer-Peppas model according to Korsmeyer et al. [89] and Ritger and Peppas [90]. Thus, to provide a more rigorous assessment of the polyphenol-release behavior, the experimental data were fitted to zero-order, first-order, Higuchi, and Korsmeyer-Peppas models (Equations (S1)–(S4), Supplementary Material, Table S9), and the obtained kinetic parameters are presented in Table 5.

The quality of fit differed among models, allowing discrimination between kinetic descriptions of the release profiles. For most formulations, the first-order model provided the best or one of the best descriptions of the 0–120 min release profiles, with *R*^2^ values ranging from 0.957 to 0.991. In the CSL series, the corresponding *R*^2^ values were 0.991, 0.967, and 0.978 for the 4, 6, and 8 wt% FOE films, respectively, whereas the zero-order fits ranged from 0.673 to 0.897 and the Higuchi fits from 0.895 to 0.964. The lower $R^{2}$ values obtained for some zero-order and Higuchi fits indicate that these models did not adequately describe all formulations over the investigated interval. Similar behavior was observed for most CSEL and CSDEA formulations, although the 8 wt% CSEL and CSDEA films showed nearly comparable zero-order and first-order fits, indicating an approximately linear release profile over the investigated early time interval.

The Korsmeyer-Peppas analysis of the 5–120 min region yielded apparent release exponents (*n*) of 0.527–0.644 for the CSL series, 0.616–1.020 for the CSEL series, and 0.766–0.926 for the CSDEA series. For most formulations, 0.5 < *n* < 1, which is consistent with anomalous (non-Fickian) transport and suggests a combined contribution of molecular diffusion and relaxation/hydration of the polymer matrix [90,91]. CSEL-FOE 8% exhibited an n value close to unity (*n* = 1.020), suggesting a particularly strong contribution of matrix-relaxation-controlled transport [90,91]. Thus, kinetic analysis does not support describing the release process as purely diffusion-controlled. Instead, the results indicate formulation-dependent transport involving both diffusion and matrix-relaxation effects, whose relative contributions vary with additive type and FOE loading.

The release behavior of polyphenols from CS-based films can be influenced by the interactions between CS and the incorporated bioactive components, as well as by the organization and physicochemical properties of the polymer matrix. CS can establish hydrogen bonding and electrostatic interactions with other components, which can affect their retention within the matrix and consequently their subsequent release [92,93,94]. Literature data have shown that the concentration and presence of CS can influence encapsulation efficiency, stability, particle characteristics, and release behavior in CS-based delivery systems [92]. In addition, CS-component interactions can modify the structural organization, hydration, and flexibility of complex systems, with the resulting properties depending on factors such as pH and component ratio [93]. CS can also contribute to the formation and stability of complex colloidal structures, demonstrating that interactions between CS and other components can substantially affect the physicochemical organization of the resulting matrix [94]. In the present study, differences in polyphenol release among CSL, CSEL, and CSDEA films may therefore be related to the combined effects of FOE loading, interactions between CS and extract/propolis constituents, and differences in matrix hydration and organization. Hence, since matrix swelling, structural changes, and molecular interactions were not directly characterized during the release experiments, these factors should be considered as possible contributors rather than experimentally confirmed mechanisms.

The results presented above demonstrate that both the type of additive and the FOE concentration influence the release behavior. The enhanced release observed in CSEL and CSDEA systems suggests that EL and DEA may modify the physicochemical organization and hydration of the matrix, thereby influencing polyphenol availability for release.

### 3.11. Antioxidant Capacity of Developed Films

**Figure 8:**
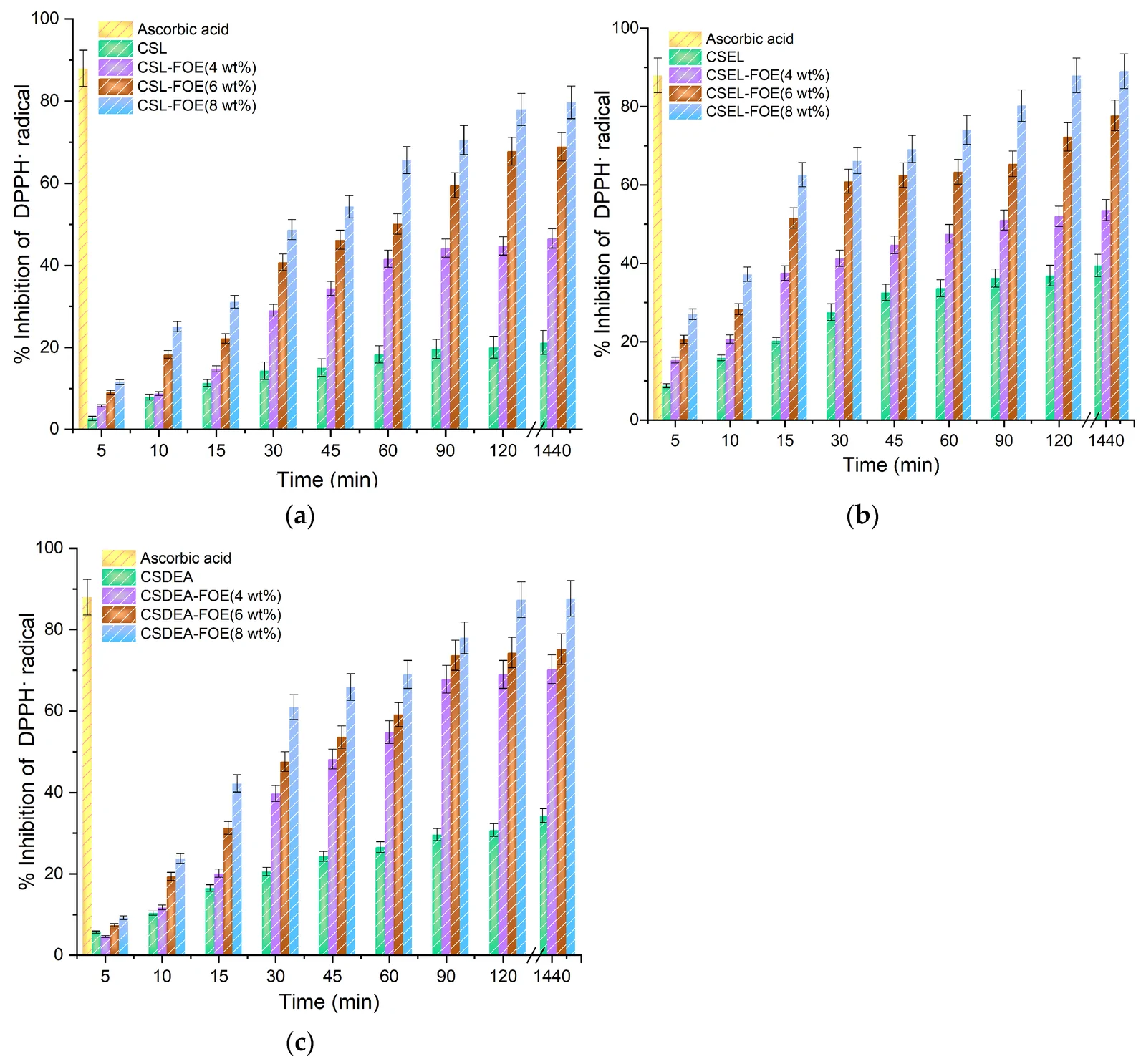
Anti-DPPH potential of developed biobased films with *Fumaria officinalis* extract (FOE) and purified propolis: (**a**) CSL and CSL-FOE (4–8 wt%), (**b**) CSEL and CSEL-FOE (4–8 wt%), and (**c**) CSDEA and CSDEA-FOE (4–8 wt%); CS—chitosan, L—limonene, EL—ethyl lactate and DEA—diethyl adipate; inhibition percentage *versus* time (phosphate-buffered saline, pH 7, ambient temperature); the break in the time axis between 120 and 1440 min is used for visualization purposes to improve the readability.

**Figure 9:**
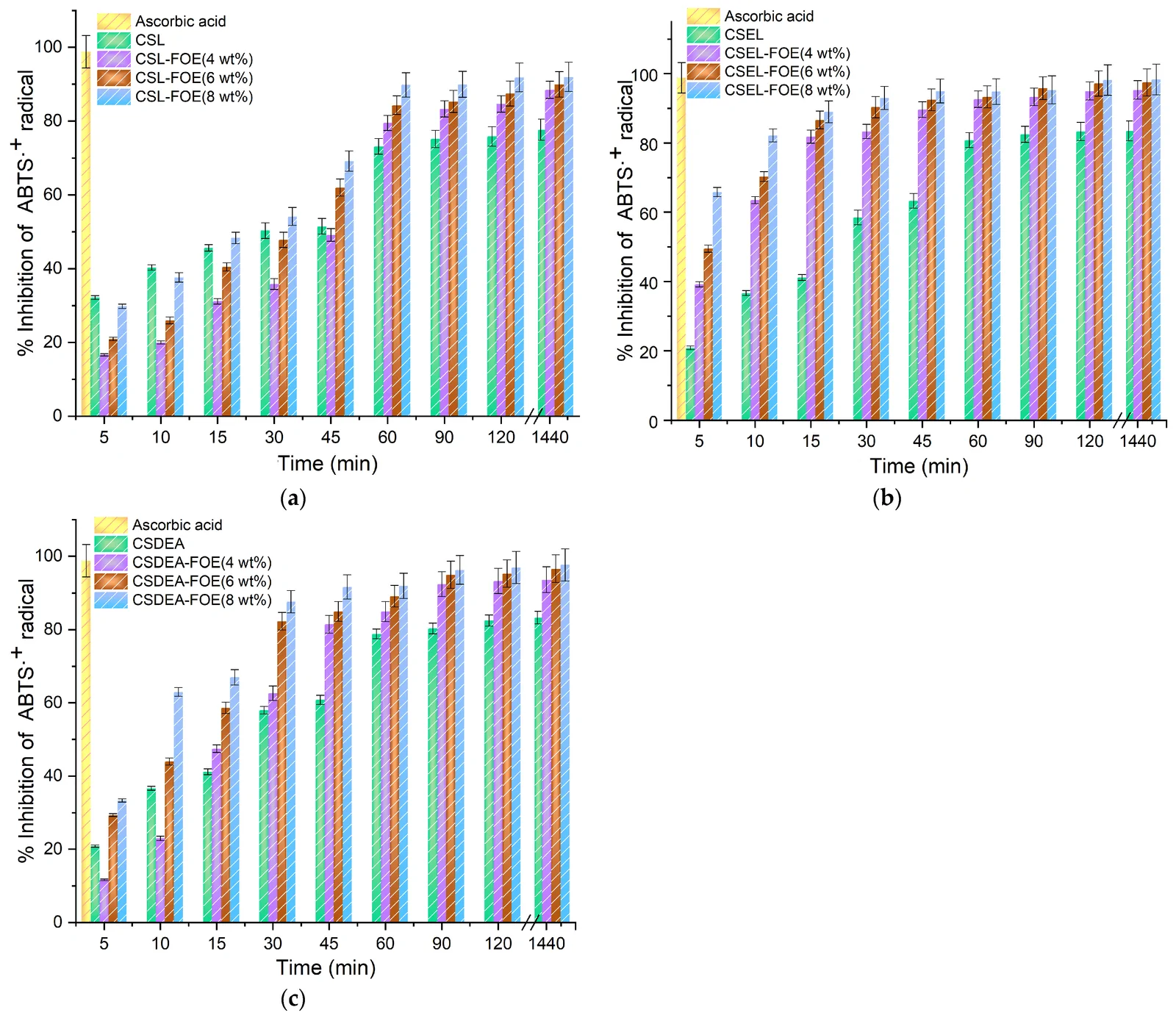
Anti-ABTS potential of developed biobased films with *Fumaria officinalis* extract (FOE) and purified propolis: (**a**) CSL and CSL-FOE (4–8 wt%), (**b**) CSEL and CSEL-FOE (4–8 wt%), and (**c**) CSDEA and CSDEA-FOE (4–8 wt%); CS—chitosan, L—limonene, EL—ethyl lactate and DEA—diethyl adipate; inhibition percentage *versus* time (phosphate-buffered saline, pH 7, ambient temperature); the break in the time axis between 120 and 1440 min is used for visualization purposes to improve the readability.

**Figure 10:**
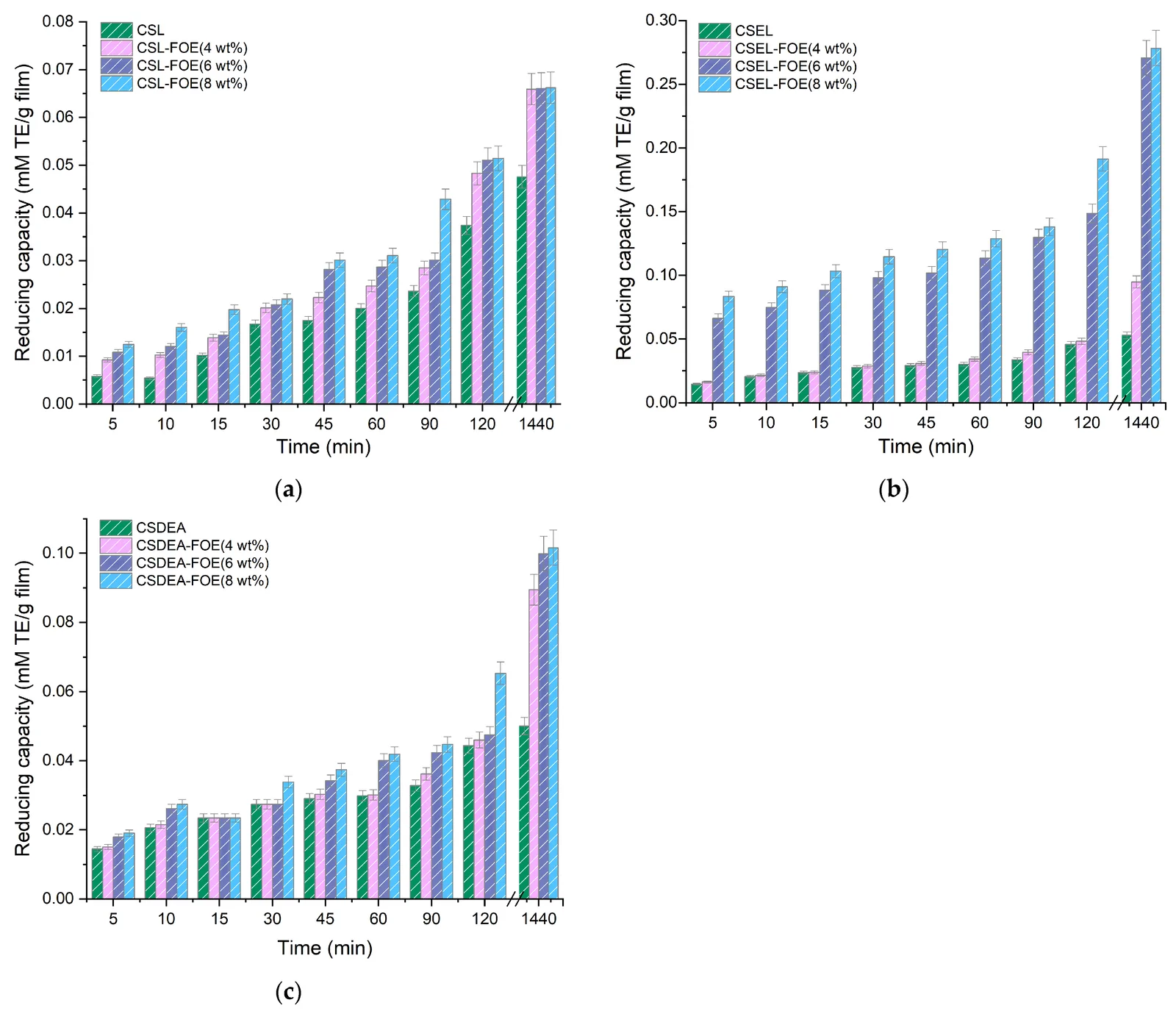
Cupric reducing antioxidant capacity of developed biobased films with *Fumaria officinalis* extract (FOE) and purified propolis: (**a**) CSL and CSL-FOE (4–8 wt%), (**b**) CSEL and CSEL-FOE (4–8 wt%), and (**c**) CSDEA and CSDEA-FOE (4–8 wt%); CS—chitosan, L—limonene, EL—ethyl lactate and DEA—diethyl adipate; reducing capacity (mmol TE/g film) *versus* time (phosphate-buffered saline, pH 7, ambient temperature); TE, Trolox equivalent; the break in the time axis between 120 and 1440 min is used for visualization purposes to improve the readability.

The chemical antioxidant activity of CS-based films incorporating FOE and purified propolis was evaluated by the DPPH, ABTS, and CUPRAC assays over time (5–1440 min). The data are presented in Figure 8, Figure 9 and Figure 10. The films containing crude propolis were subjected to the same chemical antioxidant capacity assays as the films containing purified propolis. However, the crude propolis-based films exhibited lower chemical antioxidant capacity than their purified propolis counterparts. The corresponding results are provided in the Supplementary Materials (Figures S10–S12).

The results demonstrate a clear time-dependent increase in DPPH radical inhibition across all CSL formulations with bioactives, indicating a time-dependent increase in the measured antioxidant activity of the film (Figure 8a). The CSL control film showed relatively low activity, indicating a limited intrinsic antioxidant contribution of the base formulation, consistent with reports for pure CS systems [95,96]. In this formulation, limonene was incorporated primarily for its hydrophobic matrix-modifying effect/role, which may contribute to the modulation of polymer-chain mobility, mechanical flexibility, and barrier properties of the CS matrix. Propolis and FOE, on the other hand, were included as complementary sources of bioactive compounds with antioxidant activity. Their different phytochemical profiles may provide a broader range of antioxidant properties, thereby complementing the functional role of limonene within the CS-based film. The addition of FOE/propolis was associated with higher measured radical-scavenging activity, with higher extract concentrations showing higher inhibition values. Films containing FOE showed an increase in measured radical-scavenging activity with increasing FOE content. Among the tested formulations, CSL-FOE 8 wt% showed the highest measured inhibition percentages at the evaluated time points. The observed increase may be associated with the combined contribution of the bioactive compounds present in FOE and propolis and their possible interactions within the film matrix [97,98,99,100,101,102]. Over longer incubation times (60–1440 min), all active films showed increases in measured DPPH inhibition, with the 8 wt% FOE formulation approaching or exceeding 80% inhibition. This sustained increase is consistent with gradual release of antioxidant compounds from the CS matrix. These findings highlight their potential application as dermal films with prolonged antioxidant functionality [78,103,104].

The radical-scavenging activity of the films plasticized with EL was also assessed using the DPPH assay, and the data are presented in Figure 8b. The intermediate formulations (4 or 6 wt%) also showed increases in measured inhibition values, consistent with the contribution of FOE-derived bioactive compounds, including alkaloids and phenolics [29,93], as well as additional contributions from propolis [94,97]. During extended incubation times (30–1440 min), the radical-scavenging activity increased substantially for all active films. At 1440 min, the 8 wt% FOE formulation reached approximately 89.0% inhibition, whereas the neat CSEL film showed lower measured inhibition values. This pattern is consistent with greater availability of antioxidant compounds over the incubation period and may be associated with the characteristics of the EL-modified matrix. In summary, CS films, plasticized with EL and enriched with FOE (4–8 wt%)/propolis, showed increasing measured chemical antioxidant activity over the evaluated incubation period, with higher numerical inhibition values observed at higher FOE concentrations.

All CS-based films plasticized with DEA exhibited a clear time-dependent increase in anti-DPPH activity, consistent with increasing availability of bioactive compounds during incubation (Figure 8c). The control film (without FOE and propolis) displayed only a slight improvement, confirming that the primary antioxidant contribution originates from FOE and propolis. Higher FOE content was associated with higher measured inhibition values. The 8 wt% formulation showed the highest measured radical-scavenging activity at the evaluated time points. DEA-plasticized films showed a gradual increase in measured antioxidant activity over time, with final inhibition values approaching ~87.7% at 1440 min. This behavior suggests that DEA, as a moderately hydrophobic plasticizer, enables effective dispersion of bioactive compounds while still allowing sustained antioxidant ability. Thus, DEA-plasticized films enriched with FOE (4–8 wt%)/propolis showed increasing measured antioxidant activity over the evaluated period, with the observed time-dependent response being consistent with the gradual availability of antioxidant compounds.

The time-course profiles of antioxidant activity differed among the matrix modifiers. EL-containing films showed an earlier increase in the measured DPPH inhibition values, whereas limonene-containing films showed a more gradual increase; DEA-containing films displayed a pattern between these two profiles. These differences may be related to the polarity of the additives and their potential influence on the accessibility and release of antioxidant compounds. Hence, DPPH is primarily sensitive to radical-scavenging reactions in organic media and may not fully represent antioxidant behavior under aqueous conditions. To further assess the individual contribution of the two bioactive components, additional CSEL films containing FOE or purified propolis separately were evaluated by the DPPH assay. The plain CSEL film without FOE and propolis exhibited only a very low intrinsic DPPH activity (<3%). In comparison, the CSEL film containing FOE at the level corresponding to the 6 wt% formulation, but without propolis, exhibited approximately 27% DPPH radical inhibition, whereas the film containing purified propolis at the corresponding formulation level, but without FOE, exhibited approximately 60% inhibition. These results indicate that both bioactive components contributed to the measured DPPH antioxidant response of the CSEL films under the investigated conditions, while purified propolis showed a higher individual contribution than FOE in this specific control experiment. This comparison was limited to the CSEL system at the formulation level corresponding to 6 wt% FOE and to the DPPH assay and therefore does not imply the same relative contributions for other matrix modifiers, FOE concentrations, antioxidant assays, or release behavior.

The radical neutralization capacity of the developed biobased films was also evaluated using the ABTS assay in PBS (pH 7, ambient temperature). All formulations exhibited a clear time-dependent increase in inhibition (Figure 9), consistent with progressive availability of antioxidant compounds from the film matrix. Vitamin C, used as a reference antioxidant, showed approximately complete radical inhibition. Compared with the reference, the films exhibited a slower time-dependent antioxidant response, consistent with gradual release and transport of bioactive compounds from the polymer matrix.

CSL films containing FOE and propolis showed higher measured chemical antioxidant activity than the plain film system, reaching values above 90% at extended time periods (Figure 9a). Unloaded limonene-based film showed moderate activity; however, the incorporation of FOE (4–8 wt%) was associated with higher measured chemical antioxidant activity at increasing FOE concentrations. Films with higher FOE content, particularly the 8 wt% formulation, showed higher measured scavenging activity and an earlier increase in antioxidant response over the evaluated time period. The radical-scavenging activity of films plasticized with EL showed a similar time-dependent increase in ABTS radical inhibition as observed for limonene-based systems, consistent with the time-dependent release profiles observed for these formulations (Figure 9b). However, the time-course profiles and measured inhibition values differed among the formulations containing different matrix modifiers. EL-based films showed higher measured initial radical-scavenging activity than the limonene-based systems. EL-containing samples achieved moderate inhibition values early on, likely due to higher polarity and better compatibility of EL with the CS matrix. Upon incorporation of FOE and propolis, the measured chemical antioxidant activity increased with FOE concentration. The CSEL-FOE (8 wt%) formulation showed inhibition values above 95% at 30–60 min. The limonene-based films showed lower measured initial inhibition values and higher values at later sampling times.

Films plasticized with DEA exhibited a clear time-dependent increase in anti-ABTS potential, consistent with sustained availability of active compounds over the incubation period (Figure 9c). Compared with the EL systems, DEA-based films showed a later increase in the measured inhibition values, while high inhibition values were observed at the later sampling times. The incorporation of FOE was associated with higher measured antioxidant activity at increasing FOE concentrations. The films containing 6 and 8 wt% FOE showed inhibition values above 95% after 90–120 min, with values approaching those measured for the reference antioxidant. The ABTS results showed time-dependent and formulation-related differences that were broadly consistent with the trends observed in the DPPH assay. Higher measured inhibition values at earlier sampling times were observed particularly for the EL-containing films. This may be related to the aqueous conditions of the ABTS assay, which allow interaction with both hydrophilic and relatively lipophilic antioxidant compounds. The later increase in measured inhibition values observed for the limonene-containing films may be related to the lower polarity of this matrix modifier.

The CUPRAC results for the developed films containing 4–8 wt% FOE and propolis show a pronounced time dependence of the reducing capacity measured in PBS (pH 7.0), with a continuous increase during 24 h of incubation (Figure 10).

In the initial time intervals (5–30 min), in the CSL-FOE systems, a gradual but relatively slow increase in CUPRAC reducing capacity is observed, consistent with limited availability of phenolic compounds through the polymer matrix in the early phase (Figure 10a). With increasing time, a more pronounced increase in CUPRAC values occurs, particularly after 45–90 min. The 8 wt% FOE sample showed higher measured CUPRAC values during the evaluated period, which may reflect the greater amount of antioxidant compounds initially incorporated into the film. In the formulation comparison, the measured Cu^2+^-reducing capacity was numerically highest for the 8 wt% FOE formulation, followed by 6 wt%, while the 4 wt% FOE formulation showed the lowest values. After 120 min, the values converge between the samples, while after 24 h, all systems reach a similar level, which may indicate an approach toward similar levels of available antioxidant components in the PBS medium. This pattern suggests that, despite the differences in the early antioxidant response, the formulations reached relatively similar CUPRAC values at longer incubation times. The measured initial antioxidant response was higher at increasing FOE concentrations, while the differences between formulations become less pronounced at longer incubation times.

In the initial time intervals (5–30 min), a relatively high antioxidant potential was recorded in the CSEL-FOE samples compared to the limonene system, indicating a higher hydrophilicity and permeability of the EL-modified matrix (Figure 10b). Even in the early phase, samples with higher FOE content (6 and 8 wt%) showed higher measured CUPRAC values, consistent with a higher initial reducing potential at greater extract concentrations. Over time (30–120 min), a stable, almost linear increase in CUPRAC values occurs in all samples. The 8 wt% FOE formulation showed the highest measured values, followed by 6 wt%, whereas the 4 wt% formulation showed the lowest values. After 24 h, the 6 and 8 wt% FOE formulations reached similarly high values, whereas the 4 wt% FOE formulation remained lower, indicating that the numerical differences among the formulations persisted at the final sampling point. However, compared to the limonene system, EL films showed higher measured final CUPRAC values than the limonene-based films, suggesting more efficient mobilization and higher availability of phenolic compounds in this matrix type. The EL-containing films showed an earlier increase in measured chemical antioxidant activity than the limonene-containing films under the tested conditions.

In the initial time intervals (5–30 min), a moderate activity of bioactive compounds was observed from CSDEA-FOE films; the formulations containing higher FOE content (6 and 8 wt%) showed higher measured CUPRAC values than the 4 wt% formulation (Figure 10c). This indicates that the concentration of incorporated extract directly affects the initial reducing potential and availability of phenolic compounds in the CS matrix. During the intermediate period (30–120 min), there is a gradual and stable increase in CUPRAC values for all formulations. The 8 wt% FOE formulation showed the highest measured values, followed by 6 wt%, whereas the 4 wt% formulation showed the lowest measured values. After 24 h, all samples reach high and relatively close values, indicating extensive availability of antioxidant components from the DEA matrix. Compared to the limonene system, DEA-containing films showed higher measured CUPRAC values than the limonene-containing films over the evaluated period. Compared to EL, the DEA system achieves somewhat lower final values, while still exhibiting a high and relatively stable antioxidant response over the tested period. Namely, DEA-based films showed a response between those observed for the EL- and limonene-containing systems [78,105,106,107]. The results also show that higher FOE content was associated with higher measured CUPRAC values, particularly at the earlier sampling times, while the DEA matrix allows for efficient and long-term antioxidant response.

The 8 wt% FOE formulations showed higher measured chemical antioxidant activity but also showed the greatest reduction in tensile strength, indicating that the highest bioactive loading does not necessarily represent the most suitable formulation. Thus, higher FOE loading should not be considered universally advantageous, and the antioxidant benefit must be balanced against the accompanying loss of mechanical resistance when selecting the formulation for the intended application.

The phenolic constituents tentatively identified in propolis and the reported phenolic composition of *F. officinalis* provide a plausible chemical basis for the radical-scavenging and reducing capacity observed in the present assays. Phenolic hydroxyl groups and conjugated structures can contribute to antioxidant responses through hydrogen-atom and electron-transfer mechanisms. A detailed literature-based discussion of the potential mechanisms is provided in the Supplementary Material (Section S1). However, the present experimental design does not allow the contribution of individual compounds or specific molecular mechanisms to be distinguished.

The three antioxidant assays provide complementary information on the antioxidant performance of the films. DPPH primarily evaluates radical-scavenging capacity and is more responsive to compounds that can participate in reactions under the assay conditions, whereas ABTS can interact with a broader range of hydrophilic and lipophilic antioxidants and is therefore particularly useful for complex, multiphase film systems. CUPRAC, in contrast, measures reducing capacity rather than direct radical scavenging and provides complementary information on the electron-donating ability of the released compounds. The differences among the assay responses indicate that the measured antioxidant activity may depend not only on the total amount of bioactive compounds but also on their polarity, accessibility, and availability from the film matrix. Therefore, no single assay fully describes the antioxidant behavior of the films, and the combined use of DPPH, ABTS, and CUPRAC provides a more comprehensive assessment of their antioxidant functionality. The time-dependent increase in measured chemical antioxidant capacity (Figure 8, Figure 9 and Figure 10) occurred alongside the progressive release of phenolic compounds from the film matrix (Figure 7). This temporal correspondence suggests that the availability of released bioactive constituents may contribute to the observed antioxidant response under the experimental conditions. The earlier increase in measured antioxidant activity observed for EL-containing films and the more gradual increase observed for limonene-containing films were broadly consistent with the corresponding release profiles. Additionally, it should be noted that DPPH, ABTS, and CUPRAC assays evaluate the chemical antioxidant capacity of the films under defined in vitro conditions. Therefore, the observed radical-scavenging and reducing activities should not be interpreted as direct evidence of biological antioxidant efficacy at the cellular or in vivo level. Further cell-based and in vivo studies would be required to confirm the biological antioxidant effects of the developed films. The combined use of DPPH, ABTS, and CUPRAC provides complementary information on radical-scavenging and reducing capacity and strengthens the functional characterization of the developed films.

This study has several limitations. The developed films were evaluated under controlled laboratory conditions, and their performance may differ under real application conditions. The release, antioxidant, mechanical, and morphological properties were assessed in model systems; therefore, further evaluation of long-term stability, biodegradation, biocompatibility, antimicrobial activity, and performance under application-relevant conditions is required. Future research should focus on optimizing CS film composition by varying propolis and additive ratios to improve the balance among mechanical stability, controlled release, and antioxidant activity. Studies should also investigate antimicrobial efficacy, cytotoxicity, skin compatibility, and storage stability, as well as validate the performance of the developed films in relevant topical/dermal experimental models.

## 4. Conclusions

This study demonstrated that the incorporation of FOE and propolis, combined with different matrix modifiers, provides an effective strategy for tailoring the structural, mechanical, surface, and functional properties of CS-based films. 1D and 2D NMR analyses confirmed the flavonoid-rich profile of propolis, with chrysin, galangin, pinocembrin, and pinobanksin 3-*O*-acetate confirmed by comparison with in-house reference standards, while additional flavonoid and phenolic constituents were tentatively assigned based on combined NMR data and literature comparison. FTIR supported the incorporation of the bioactive components and revealed spectral changes consistent with modifications in the local chemical environment and intermolecular associations within the CS matrix. Purification of propolis resulted in enrichment of phenolic functional groups and removal of less polar components, particularly waxes and lipids. The combined ^1^H and ^13^C NMR, supported by FTIR analysis, confirmed the successful synthesis and structural integrity of TGC and DEA, with spectral features consistent with the proposed ester structures and indicating high degrees of esterification and product purity. Morphological analyses showed that film structure was strongly dependent on additive type and FOE concentration. EL-containing films exhibited comparatively uniform surfaces, DEA-containing films showed the most compact cross-sectional morphology, whereas limonene-containing formulations displayed greater surface heterogeneity and porosity, particularly at higher FOE loading. Mechanical testing demonstrated a clear trade-off associated with increasing FOE concentration: stress at break decreased substantially in all three active-film series, while the CSEL formulations retained comparatively favorable tensile resistance together with moderate strain at break. Surface wettability and swelling behavior also depended on both matrix modifier and FOE concentration, with higher FOE loading associated with reduced water uptake, although aqueous stability remained strongly formulation-dependent. Polyphenol release occurred progressively over the 24 h evaluation period, but the magnitude and concentration dependence of release varied among the CSL, CSEL, and CSDEA systems. Kinetic modeling indicated formulation-dependent transport behavior consistent with contributions from both molecular diffusion and matrix relaxation/hydration rather than purely Fickian diffusion. FOE/propolis-containing films exhibited measurable time-dependent chemical antioxidant capacity in the DPPH, ABTS, and CUPRAC assays, with higher FOE loadings generally associated with higher antioxidant responses but also with greater loss of tensile strength. The incorporation of FOE and propolis resulted in measurable antioxidant activity of the films, while higher FOE loadings were also associated with reductions in mechanical strength, highlighting the need to consider the relationship between bioactive loading and mechanical integrity. Namely, the developed CS-based films represent promising tunable biobased systems with potential for future evaluation in controlled-release applications, particularly as functional films for topical delivery. Nevertheless, their suitability for such an application requires further validation, including biocompatibility, biodegradability, cytotoxicity, skin compatibility, skin-related effects, antimicrobial activity, and long-term stability studies.

## Figures and Tables

**Table 1:** Nuclear magnetic resonance (NMR) data of the metabolites identified in the propolis samples.

Identified Compound	Chemical Shifts (ppm)
Chrysin *	*δ* 12.82 (s), *δ* 8.02 (m), *δ* 7.55 (m), *δ* 6.96 (s), *δ* 6.53 (d, *J* = 2.1 Hz), *δ* 6.23 (d, *J* = 2.1 Hz)
Galangin *	*δ* 12.36 (s), *δ* 8.12 (m), *δ* 7.51 (m), *δ* 6.47 (d, *J* = 2.1 Hz), *δ* 6.22 (d, *J* = 2.1 Hz)
Pinobanksin	*δ* 11.89 (s), *δ* 7.52 (m), *δ* 7.41 (m), *δ* 6.18 (d, *J* = 2.1), *δ* 5.92 (d, *J* = 2.1), *δ* 5.18 (d, *J* = 11.4 Hz), *δ* 5.07 (d, *J* = 11.4 Hz)
Pinocembrin *	*δ* 12.12 (s), *δ* 7.52 (m), *δ* 7.41 (m), *δ* 5.97 (d, *J* = 2.1 Hz), *δ* 5.95 (d, *J* = 2.1 Hz), *δ* 5.58 (dd, *J* = 12.7, 3.0 Hz), *δ* 3.24 (dd, *J* = 17.3, 12.7 Hz), *δ* 2.78 (dd, *J* = 17.3, 3.0 Hz)
Pinobanksin 3-*O*-acetate *	*δ* 11.44 (s), *δ* 7.53 (m), *δ* 7.43 (m), *δ* 5.98 (d, *J* = 2.2 Hz), *δ* 5.95 (d, *J* = 2.2 Hz), *δ* 5.93 (d, *J* = 11.8 Hz), *δ* 5.59 (d, *J* = 11.8 Hz), *δ* 1.94 (s)
Pinobanksin 3-*O*-methylether	*δ* 7.49 (m), *δ* 7.40 (m), *δ* 6.11 (d, *J* = 2.1 Hz), *δ* 5.95 (d, *J* = 2.1 Hz), *δ* 5.07 (d, *J* = 11.8 Hz), *δ* 4.33 (d, *J* = 11.8 Hz), *δ* 3.77 (s)
Kaempferide	*δ* 12.69 (s), *δ* 7.89 (d, *J* = 9.0 Hz), *δ* 6.93 (d, *J* = 9.0 Hz), *δ* 6.35 (d, *J* = 2.2 Hz), *δ* 6.09 (d, *J* = 2.2 Hz), *δ* 3.77 (s)
Apigenin	*δ* 12.96 (s), *δ* 7.93 (d, *J* = 9.1 Hz), *δ* 6.95 (d, *J* = 9.1 Hz), *δ* 6.76 (s), *δ* 6.48 (d, *J* = 2.0 Hz), *δ* 6.20 (d, *J* = 2.0 Hz)
Naringenin	*δ* 7.31 (AA′BB′), *δ* 6.79 (AA′BB′), *δ* 5.89 (S), *δ* 5.43 (dd, *J* = 12.6, 2.8 Hz), *δ* 3.26 (dd, *J* = 17.1, 12.6 Hz), *δ* 2.68 (dd, *J* = 17.1, 2.8 Hz)
Caffeic acid	*δ* 7.42 (d, *J* = 16.0 Hz), *δ* 7.05 (d, *J* = 1.8 Hz), 6.98 (dd, *J* = 8.1, 1.8 Hz), *δ* 6.77 (d, *J* = 8.1 Hz), *δ* 6.18 (d, *J* = 16.0 Hz)
Benzyl caffeate	Caffeate moiety: *δ* 7.52 (d, *J* = 15.9 Hz), *δ* 7.05 (d, *J* = 1.8 Hz), 7.00 (dd, *J* = 8.1, 1.8 Hz), *δ* 6.76 (d, *J* = 8.1 Hz), *δ* 6.32 (d, *J* = 15.9 Hz)
	Benzyl moiety: *δ* 7.38 (m), *δ* 5.19 (s)
Caffeic acid phenethyl ester	Caffeic acid moiety: *δ* 7.52 (d, *J* = 15.9 Hz), *δ* 7.04 (d, *J* = 1.8 Hz), *δ* 7.01 (dd, *J* = 8.1, 1.8 Hz), *δ* 6.79 (d, *J* = 8.1 Hz), *δ* 6.32 (d, *J* = 15.9 Hz)
	Phenethyl moiety: *δ* 7.26 (m), *δ* 4.31 (t, *J* = 6.9 Hz), *δ* 2.94 (t, *J* = 6.9 Hz)
Cinnamyl caffeate	Caffeic moiety: *δ* 7.46 (d, *J* = 15.9 Hz), *δ* 7.05 (d, *J* = 1.8 Hz), *δ* 7.01 (dd, *J* = 8.1, 1.8 Hz), *δ* 6.76 (d, *J* = 8.1 Hz), *δ* 6.25 (d, *J* = 15.9 Hz)
	Cinnamyl moiety: *δ* 7.46 (m), *δ* 6.71 (dt, *J* = 16.0, 1.8 Hz), *δ* 6.42 (dt, *J* = 16.0, 6.2 Hz), *δ* 4.80 (dd, *J* = 6.2, 1.8 Hz)
*p*-Coumaric acid	*δ* 7.51 (d, *J* = 8.2 Hz), *δ* 7.49 (d, *J* = 16.0 Hz), *δ* 6.78 (d, *J* = 8.2 Hz), *δ* 6.29 (d, *J* = 16.0 Hz)
Ferulic acid	*δ* 7.49 (d, *J* = 16.0 Hz), *δ* 7.27 (d, *J* = 2.0 Hz), *δ* 7.08 (dd, *J* = 8.1, 2.0 Hz), *δ* 6.79 (d, *J* = 8.1 Hz), *δ* 6.36 (d, *J* = 16.0 Hz), *δ* 3.81 (s).
Benzoic acid	*δ* 7.49 (dd, *J* = 8.6, 1.2 Hz), *δ* 7.61 (tt, *J* = 7.5, 1.2 Hz), *δ* 7.95 (dd, *J* = 8.6, 7.5 Hz)

* Assigned based on 2D NMR spectra and reference in-house data from purified compounds [44]; other components were tentatively identified based on 1D/2D NMR chemical shifts and literature data.

**Table 2:** Break stress and break strain of the developed biobased films in the absence and presence of *Fumaria officinalis* extract (FOE) and propolis.

Film	Thickness (mm)	Stress at Break (N/mm^2^)	Strain at Break (%)
CSDEA	0.323 ± 0.01 *	46.12 ± 1.16	8.10 ± 1.32
CSDEA-FOE (4 wt%)	0.335 ± 0.03 ^d^	41.93 ± 1.84 ^a^	10.34 ± 0.96 ^cd^
CSDEA-FOE (6 wt%)	0.348 ± 0.06 ^bc^	33.41 ± 2.01 ^b^	6.73 ± 1.13 ^e^
CSDEA-FOE (8 wt%)	0.352 ± 0.03 ^b^	14.25 ± 1.59 ^e^	11.44 ± 1.01 ^bc^
CSEL	0.270 ± 0.03	53.58 ± 0.13	14.05 ± 1.63
CSEL-FOE (4 wt%)	0.278 ± 0.05 ^e^	44.42 ± 1.09 ^a^	11.77 ± 0.83 ^bc^
CSEL-FOE (6 wt%)	0.282 ± 0.09 ^e^	32.83 ± 0.94 ^b^	9.48 ± 1.07 ^cd^
CSEL-FOE (8 wt%)	0.284 ± 0.02 ^e^	22.80 ± 1.14 ^d^	10.37 ± 1.26 ^bcd^
CSL	0.330 ± 0.02	34.19 ± 1.02	10.64 ± 1.00
CSL-FOE (4 wt%)	0.342 ± 0.01 ^c^	30.35 ± 0.58 ^c^	14.81 ± 1.41 ^a^
CSL-FOE (6 wt%)	0.369 ± 0.01 ^a^	14.78 ± 0.97 ^e^	12.06 ± 0.53 ^b^
CSL-FOE (8 wt%)	0.376 ± 0.08 ^a^	10.98 ± 0.54 ^f^	14.52 ± 1.45 ^a^

* CS—chitosan, L—limonene, EL—ethyl lactate and DEA—diethyl adipate; data are presented as mean ± standard deviation (*n* = 3). The effects of additive type, FOE concentration, and their interaction were evaluated by two-way analysis of variance (ANOVA), followed by Tukey’s multiple-comparison test. Within each parameter (for films with FOE/propolis), means that do not share a common superscript letter are significantly different (*p* < 0.05).

**Table 3:** Water contact angle measurements of the developed biobased films with *Fumaria officinalis* extract (FOE) and propolis.

Film	Water Contact Angle (°)
CSL	57.4 ± 0.9
CSL-FOE (4 wt%)	56.4 ± 2.0 ^c^*
CSL-FOE (6 wt%)	57.1 ± 1.4 ^bc^
CSL-FOE (8 wt%)	60.3 ± 2.5 ^bc^
CSEL	50.5 ± 1.8
CSEL-FOE (4 wt%)	60.7 ± 2.0 ^bc^
CSEL-FOE (6 wt%)	59.5 ± 1.2 ^bc^
CSEL-FOE (8 wt%)	62.5 ± 2.1 ^b^
CSDEA	53.5 ± 0.6
CSDEA-FOE (4 wt%)	58.7 ± 1.1 ^bc^
CSDEA-FOE (6 wt%)	60.2 ± 3.6 ^bc^
CSDEA-FOE (8 wt%)	69.1 ± 1.0 ^a^

* CS—chitosan, L—limonene, EL—ethyl lactate and DEA—diethyl adipate; data are presented as mean ± standard deviation (*n* = 3). The effects of additive type, FOE concentration, and their interaction were evaluated by two-way analysis of variance (ANOVA), followed by Tukey’s test. Within each parameter (for films with FOE/propolis), means that do not share a common superscript letter are significantly different (*p* < 0.05).

**Table 4:** Swelling ratio of the developed biobased films with *Fumaria officinalis* extract (FOE) and propolis after immersion in phosphate-buffered saline (pH 7.0).

Film	Swelling Ratio Over Time				
	30 min	60 min	90 min	120 min	150 min
CSL *	5.19 ± 0.68	5.27 ± 0.39	5.22 ± 0.95	5.38 ± 1.01	5.69 ± 0.91
CSL-FOE (4 wt%)	6.69 ± 0.75 ^bcd^	7.48 ± 1.04 ^bc^	7.66 ± 1.05 ^abc^	7.04 ± 0.85 ^ab^	6.42 ± 1.02 ^ab^
CSL-FOE (6 wt%)	7.00 ± 1.03 ^abcd^	7.25 ± 1.32 ^bc^	6.34 ± 1.09 ^abcd^	-	-
CSL-FOE (8 wt%)	4.07 ± 0.95 ^e^	4.20 ± 0.68 ^d^	4.11 ± 0.54 ^e^	4.00 ± 0.24 ^c^	3.59 ± 0.84 ^c^
CSEL	5.51 ± 0.45	5.18 ± 0.74	5.63 ± 0.48	5.37 ± 0.27	5.80 ± 0.55
CSEL-FOE (4 wt%)	6.97 ± 0.63 ^abcd^	7.26 ± 0.74 ^c^	6.56 ± 0.33 ^abcd^	6.51 ± 0.83 ^ab^	6.16 ± 0.89 ^abc^
CSEL-FOE (6 wt%)	7.02 ± 0.83 ^abc^	7.33 ± 1.06 ^bc^	7.25 ± 0.97 ^abc^	7.41 ± 1.21 ^ab^	6.98 ± 1.15 ^a^
CSEL-FOE (8 wt%)	4.64 ± 1.01 ^de^	4.88 ± 0.87 ^d^	4.24 ± 0.74 ^de^	3.91 ± 0.82 ^c^	3.88 ± 0.95 ^bc^
CSDEA	8.64 ± 0.70	8.92 ± 0.75	8.45 ± 0.81	8.12 ± 0.85	7.82 ± 0.94
CSDEA-FOE (4 wt%)	9.29 ± 0.65 ^a^	9.61 ± 1.11 ^a^	8.70 ± 1.21 ^a^	8.48 ± 0.99 ^a^	8.23 ± 1.19 ^a^
CSDEA-FOE (6 wt%)	8.96 ± 0.79 ^ab^	7.80 ± 0.84 ^bc^	-	-	-
CSDEA-FOE (8 wt%)	6.82 ± 0.94 ^bcd^	7.09 ± 0.61 ^c^	6.98 ± 0.27 ^abc^	6.53 ± 0.70 ^ab^	6.32 ± 1.17 ^abc^

* CS—chitosan, L—limonene, EL—ethyl lactate and DEA—diethyl adipate; data are presented as mean ± standard deviation (*n* = 3); different superscript letters within the same time point (for the films with FOE/propolis) indicate statistically significant differences according to Tukey’s test (*p* < 0.05); the dash denotes film disintegration after a certain period of immersion, preventing determination of swelling ratio.

**Table 5:** Kinetic parameters obtained by nonlinear fitting of the polyphenol-release data using zero-order, first-order, Higuchi, and Korsmeyer–Peppas models for developed films.

Model	Eq. Parameter	CSL		
		CSL-FOE (4 wt%)	CSL-FOE (6 wt%)	CSL-FOE (8 wt%)
Zero-order	*K*_0_ (min^−1^)	0.00313 ± 0.00030	0.00277 ± 0.00034	0.00306 ± 0.00021
	*R* ^2^	0.858	0.673	0.897
First-order	*K*_1_ (min^−1^)	0.01774 ± 0.0022	0.02791 ± 0.00539	0.01401 ± 0.0030
	*F∞*	0.3542 ± 0.0220	0.26863 ± 0.02058	0.3872 ± 0.0460
	*R* ^2^	0.991	0.967	0.978
Higuchi	*K*_H_ (min^−0.5^)	0.02815 ± 0.0013	0.02549 ±0.0017	0.02744 ± 0.00100
	*R* ^2^	0.951	0.895	0.964
Korsmeyer–Peppas	*K_KP_* (min^−*n*^)	0.0169 ± 0.0049	0.02273 ± 0.01114	0.0150 ± 0.0027
	*n*	0.6240 ± 0.0670	0.5274 ± 0.1100	0.6435 ± 0.0410
	*R* ^2^	0.962	0.863	0.986
Model	**Eq. parameter**	**CSEL**		
		**CSEL-FOE (4 wt%)**	**CSEL-FOE (6 wt%)**	**CSEL-FOE (8 wt%)**
Zero-order	*K*_0_ (min^−1^)	0.00330 ± 0.00020	0.00342 ± 0.00028	0.00352 ± 0.00013
	*R* ^2^	0.935	0.855	0.979
First-order	*K*_1_ (min^−1^)	0.00878 ± 0.00360	0.01764 ± 0.00260	0.00422 ± 0.00340
	*F∞*	0.54964 ± 0.15350	0.38822 ± 0.02800	0.8921 ± 0.6600
	*R* ^2^	0.969	0.987	0.970
Higuchi	*K*_H_ (min^−0.5^)	0.02875 ± 0.00253	0.03082 ± 0.0013	0.02990 ± 0.0033
	*R* ^2^	0.870	0.956	0.824
Korsmeyer–Peppas	*K_KP_* (min^−*n*^)	0.00766 ± 0.00393	0.01891 ± 0.0051	0.00321 ± 0.0014
	*n*	0.8115 ± 0.1100	0.6159 ± 0.0620	1.0201 ± 0.0980
	*R* ^2^	0.947	0.967	0.976
Model	**Eq. parameter**	**CSDEA**		
		**CSDEA-FOE (4 wt%)**	**CSDEA-FOE (6 wt%)**	**CSDEA-FOE (8 wt%)**
Zero-order	*K*_0_ (min^−1^)	0.00335 ± 0.00019	0.00339 ± 0.00022	0.00352 ± 0.00010
	*R* ^2^	0.939	0.927	0.985
First-order	*K*_1_ (min^−1^)	0.01036 ± 0.00240	0.00856 ± 0.00420	0.00426 ± 0.00210
	*F∞*	0.50185 ± 0.07400	0.57422 ± 0.19000	0.9670 ± 0.4100
	*R* ^2^	0.987	0.957	0.987
Higuchi	*k*_H_ (min^−0.5^)	0.02938 ± 0.00213	0.02955 ± 0.00270	0.03028 ± 0.00270
	*R* ^2^	0.904	0.862	0.864
Korsmeyer–Peppas	*K_KP_* (min^−*n*^)	0.00954 ± 0.00330	0.00783 ± 0.00440	0.00490 ± 0.0015
	*n*	0.7655 ± 0.0770	0.8128 ± 0.1300	0.9261 ± 0.0670
	*R* ^2^	0.972	0.937	0.985

## Data Availability

The original contributions presented in this study are included in the article/Supplementary Materials. Further inquiries can be directed to the corresponding author. All figures, tables, and graphical abstract images in the manuscript are original and were created by the authors. No material from other publications has been reproduced, and therefore no copyright permissions are required.

## Supplementary Materials

The following supporting information can be downloaded at: https://www.mdpi.com/article/10.3390/antiox15101265/s1, References [108,109,110,111,112,113,114,115,116,117,118,119,120,121] are cited in the Supplementary Materials.

## Abbreviations

The following abbreviations are used in this manuscript:

ABTS	2,2′-Azino-bis(3-ethylbenzothiazoline-6-sulfonic acid)
AFM	Atomic force microscopy
CUPRAC	Cupric ion reducing antioxidant capacity
CS	Chitosan
DEA	Diethyl adipate
DMA	Dynamic mechanical analysis
DPPH	2,2-Diphenyl-1-picrylhydrazyl
EL	Ethyl lactate
FESEM	Field emission scanning electron microscopy
FOE	*Fumaria officinalis* extract
FTIR	Fourier transform infrared
NMR	Nuclear magnetic resonance
PBS	Phosphate-buffered saline
SR	Swelling ratio
TGC	Triglyceryl citrate
TPC	Total polyphenol content
