# Supplementary material for: Influence of Limonene, Ethyl Lactate, and Diethyl Adipate on the Functional Properties of Chitosan-Based Films Loaded with Fumaria officinalis L. Extract and Propolis
Source: Antioxidants (Basel). 2026 Oct 1;15(10):1265. doi: 10.3390/antiox15101265 (PMC13700680; doi:10.3390/antiox15101265)
Supplement: Supplementary file 1 [file antioxidants-15-01265-s001.zip › antioxidants-4529386-supplementary.pdf]

Supplementary Materials

Influence of Limonene, Ethyl Lactate, and Diethyl Adipate on the Functional Properties of Chitosan-Based Films Loaded with *Fumaria officinalis* L. Extract and Propolis

Rabiea Ashowen Ahmoda[1], Milena Milošević[2], Tihomir Kovačević[3], Sanja Savić[1], Ivona Janković-Častvan[1], Boban Anđelković[4], Tamara Erceg[5], Aleksandra A. Jovanović[6*], Aleksandar Marinković[1]

[1] Faculty of Technology and Metallurgy, University of Belgrade, Karnegijeva 4, 11000 Belgrade, Serbia; 20214039@estudent.tmf.bg.ac.rs (R.A.A.), sseslija@tmf.bg.ac.rs (S.S.), icastvan@tmf.bg.ac.rs (I.J.-Č.), marinko@tmf.bg.ac.rs (A.M.)
[2] Institute of Chemistry, Technology and Metallurgy - National Institute of the Republic of Serbia, University of Belgrade, Njegoševa 12, 11000 Belgrade, Serbia; milena.milosevic@ihtm.bg.ac.rs
[3] Military Technical Institute, Ratka Resanovića 1, 11030 Belgrade, Serbia; tkovacevic@tmf.bg.ac.rs
[4] Faculty of Chemistry, University of Belgrade, Studentski trg 12-16, 11000 Belgrade, Serbia; aboban@chem.bg.ac.rs
[5] Faculty of Technology Novi Sad, University of Novi Sad, Bulevar Cara Lazara 1, 21102 Novi Sad, Serbia; tamara.erceg@uns.ac.rs
[6] Institute for the Application of Nuclear Energy INEP, University of Belgrade, Banatska 31b, 11080 Belgrade, Serbia; ajovanovic@inep.co.rs

* Correspondence: ajovanovic@inep.co.rs

Table S1. Nominal composition of the chitosan (CS)-based film formulations based on the initial amounts of non-volatile components before drying (g and wt%); FOE, *Fumaria officinalis* extract; L, limonene; EL, ethyl lactate; DEA, diethyl adipate.

Film	CS	Lactic acid	Triglyceryl citrate	Propolis	Glucopon®	Secondary additive	FOE	Total dry mass
**CSL**	5.000 g (48.08%)	2.000 g (19.23%)	1.500 g (14.42%)	-	0.400 g (3.85%)	Limonene 1.500 g (14.42%)	-	10.400 g
**CSL-FOE (4 wt%)**	5.000 g (41.02%)	2.000 g (16.41%)	1.500 g (12.31%)	1.300 g (10.66%)	0.400 g (3.28%)	Limonene 1.500 g (12.31%)	0.490 g (4.02%)	12.190 g
**CSL-FOE (6 wt%)**	5.000 g (40.16%)	2.000 g (16.06%)	1.500 g (12.05%)	1.300 g (10.44%)	0.400 g (3.21%)	Limonene 1.500 g (12.05%)	0.750 g (6.02%)	12.450 g
**CSL-FOE (8 wt%)**	5.000 g (39.31%)	2.000 g (15.72%)	1.500 g (11.79%)	1.300 g (10.22%)	0.400 g (3.14%)	Limonene 1.500 g (11.79%)	1.020 g (8.02%)	12.720 g
**CSEL**	5.000 g (48.08%)	2.000 g (19.23%)	1.500 g (14.42%)	-	0.400 g (3.85%)	EL 1.500 g (14.42%)	-	10.400 g
**CSEL-FOE (4 wt%)**	5.000 g (41.02%)	2.000 g (16.41%)	1.500 g (12.31%)	1.300 g (10.66%)	0.400 g (3.28%)	EL 1.500 g (12.31%)	0.490 g (4.02%)	12.190 g
**CSEL-FOE (6 wt%)**	5.000 g (40.16%)	2.000 g (16.06%)	1.500 g (12.05%)	1.300 g (10.44%)	0.400 g (3.21%)	EL 1.500 g (12.05%)	0.750 g (6.02%)	12.450 g
**CSEL-FOE (8 wt%)**	5.000 g (39.31%)	2.000 g (15.72%)	1.500 g (11.79%)	1.300 g (10.22%)	0.400 g (3.14%)	EL 1.500 g (11.79%)	1.020 g (8.02%)	12.720 g
**CSEL-FOE (6 wt%, no propolis)[1]**	5.000 g (44.84%)	2.000 g (17.94%)	1.500 g (13.45%)	-	0.400 g (3.59%)	EL 1.500g (13.45%)	0.750 g (6.73%)	11.150 g
**CSEL-Propolis (no FOE)**	5.000 g (42.74%)	2.000 g (17.09%)	1.500 g (12.82%)	1.300 g (11.11%)	0.400 g (3.42%)	EL 1.500g (12.82%)	-	11.700 g
**CSDEA**	5.000 g (48.08%)	2.000 g (19.23%)	1.500 g (14.42%)	-	0.400 g (3.85%)	DEA 1.500 g (14.42%)	-	10.400 g
**CSDEA-FOE (4 wt%)**	5.000 g (41.02%)	2.000 g (16.41%)	1.500 g (12.31%)	1.300 g (10.66%)	0.400 g (3.28%)	DEA 1.500 g (12.31%)	0.490 g (4.02%)	12.190 g
**CSDEA-FOE (6 wt%)**	5.000 g (40.16%)	2.000 g (16.06%)	1.500 g (12.05%)	1.300 g (10.44%)	0.400 g (3.21%)	DEA 1.500 g (12.05%)	0.750 g (6.02%)	12.450 g
**CSDEA-FOE (8 wt%)**	5.000 g (39.31%)	2.000 g (15.72%)	1.500 g (11.79%)	1.300 g (10.22%)	0.400 g (3.14%)	DEA 1.500 g (11.79%)	1.020 g (8.02%)	12.720 g

[1]For the additional FOE-only CSEL control, 0.75 g FOE was used, corresponding to the same absolute FOE amount as in the CSEL-FOE (6 wt%) formulation; because propolis was omitted, its calculated wt% in this control differs from 6 wt%.

Table S2. Estimated composition of the final dried chitosan (CS)-based films after correction for the apparent retention of limonene, ethyl lactate (EL), and diethyl adipate (DEA) (g and wt%); FOE, *Fumaria officinalis* extract; L, limonene.

Film	CS	Lactic acid	Triglyceryl citrate	Propolis	Glucopon®	Secondary additive	FOE	Total dry mass
**CSL**	5.000 g (51.10%)	2.000 g (20.44%)	1.500 g (15.33%)	-	0.400 g (4.09%)	Limonene 0.885 g (9.04%)	-	9.785 g
**CSL-FOE (4 wt%)**	5.000 g (43.20%)	2.000 g (17.28%)	1.500 g (12.96%)	1.300 g (11.23%)	0.400 g (3.46%)	Limonene 0.885 g (7.65%)	0.490 g (4.23%)	11.575 g
**CSL-FOE (6 wt%)**	5.000 g (42.25%)	2.000 g (16.90%)	1.500 g (12.67%)	1.300 g (10.98%)	0.400 g (3.38%)	Limonene 0.885 g (7.48%)	0.750 g (6.34%)	11.835 g
**CSL-FOE (8 wt%)**	5.000 g (41.31%)	2.000 g (16.52%)	1.500 g (12.39%)	1.300 g (10.74%)	0.400 g (3.30%)	Limonene 0.885 g (7.31%)	1.020 g (8.43%)	12.105 g
**CSEL**	5.000 g (49.58%)	2.000 g (19.83%)	1.500 g (14.87%)	-	0.400 g (3.97%)	EL 1.185 g (11.75%)	-	10.085 g
**CSEL-FOE (4 wt%)**	5.000 g (42.11%)	2.000 g (16.84%)	1.500 g (12.63%)	1.300 g (10.95%)	0.400 g (3.37%)	EL 1.185 g (9.98%)	0.490 g (4.13%)	11.875 g
**CSEL-FOE (6 wt%)**	5.000 g (41.20%)	2.000 g (16.48%)	1.500 g (12.36%)	1.300 g (10.71%)	0.400 g (3.30%)	EL 1.185 g (9.77%)	0.750 g (6.18%)	12.135 g
**CSEL-FOE (8 wt%)**	5.000 g (40.31%)	2.000 g (16.12%)	1.500 g (12.09%)	1.300 g (10.48%)	0.400 g (3.22%)	EL 1.185 g (9.55%)	1.020 g (8.22%)	12.405 g
**CSEL-FOE (6 wt%, no propolis)**	5.000 g (46.15%)	2.000 g (18.46%)	1.500 g (13.84%)	-	0.400 g (3.69%)	EL 1.185 g (10.94%)	0.750 g (6.92%)	10.835 g
**CSEL-Propolis (no FOE)**	5.000 g (43.92%)	2.000 g (17.57%)	1.500 g (13.18%)	1.300 g (11.42%)	0.400 g (3.51%)	EL 1.185 g (10.41%)	-	11.385 g
**CSDEA**	5.000 g (48.71%)	2.000 g (19.48%)	1.500 g (14.61%)	-	0.400 g (3.90%)	DEA 1.365 g (13.30%)	-	10.265 g
**CSDEA-FOE (4 wt%)**	5.000 g (41.48%)	2.000 g (16.59%)	1.500 g (12.44%)	1.300 g (10.78%)	0.400 g (3.32%)	DEA 1.365 g (11.32%)	0.490 g (4.06%)	12.055 g
**CSDEA-FOE (6 wt%)**	5.000 g (40.60%)	2.000 g (16.24%)	1.500 g (12.18%)	1.300 g (10.56%)	0.400 g (3.25%)	DEA 1.365 g (11.08%)	0.750 g (6.09%)	12.315 g
**CSDEA-FOE (8 wt%)**	5.000 g (39.73%)	2.000 g (15.89%)	1.500 g (11.92%)	1.300 g (10.33%)	0.400 g (3.18%)	DEA 1.365 g (10.85%)	1.020 g (8.10%)	12.585 g

Note: Estimated final compositions were calculated using apparent retentions of 59% for limonene, 79% for EL, and 91% for DEA. All other non-volatile components were assumed to be fully retained. The values should be regarded as semi-quantitative because additive retention was determined gravimetrically rather than by direct chemical analysis.

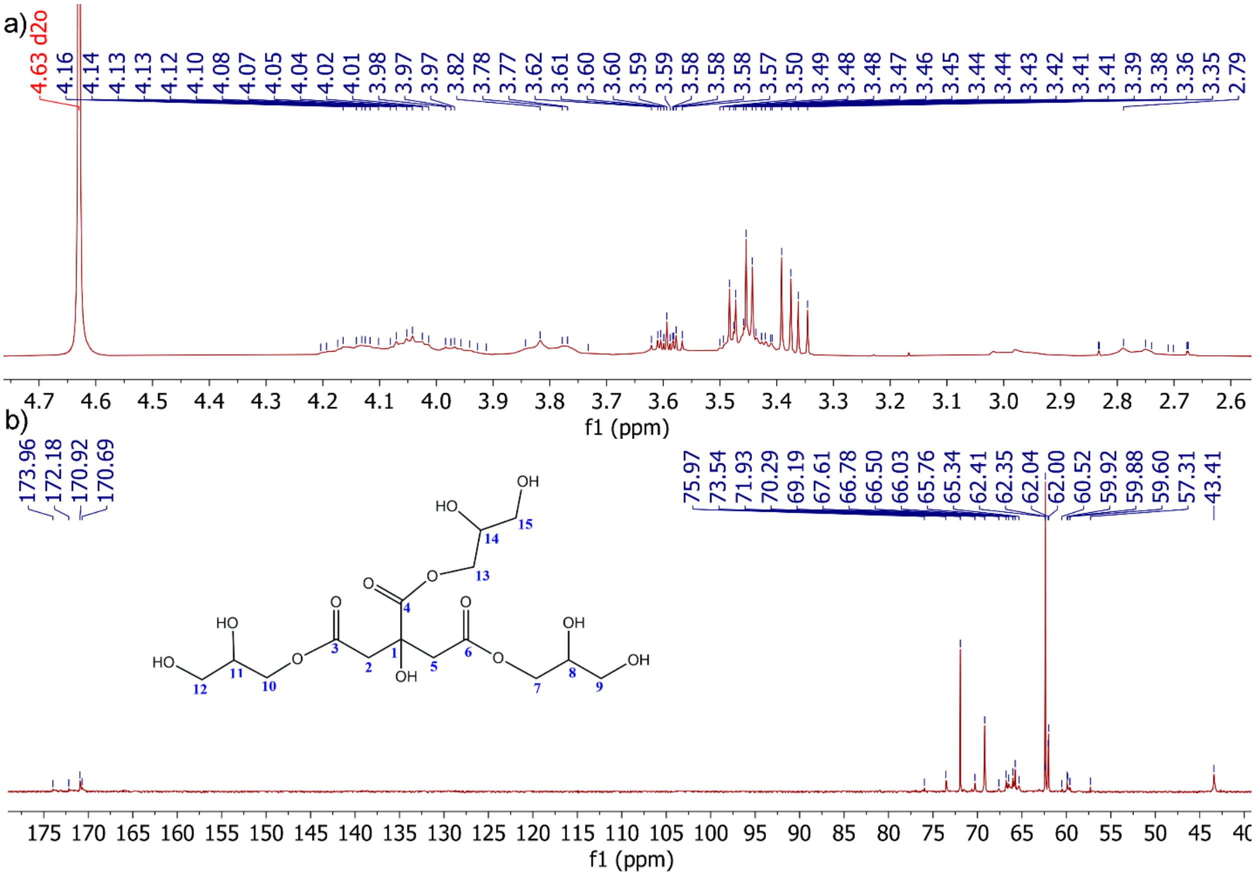

Figure S1. a) ^{1}H nuclear magnetic resonance (NMR) and b) ^{13}C NMR spectra of triglyceryl citrate. ^{1}H NMR ($CDCl_3$, 400 MHz, δ/ppm): 2.79-2.836 (4H, *m*, H-2 and H-5), 3.35-3.50 (6H, *m*, H-9, H-12, and H-15), 3.60-3.82 (3H, *m*, H-8, H-11, and H-14), and 3.97-4.16 (4H, *m*, H-7, H-10, and H-13). ^{13}C NMR ($CDCl_3$, 100 MHz, δ/ppm): 43.41 (C(2) and C(5)), 57.3-62.4 (C(9), C(12), and C(15)), 65.3-67.6 (C(7), C(10), and C(13)), 69.2-71.9 (C(8), C(11), and C(14)), 170.7-174.0 (C(3), (C4), and C(6)).

The successful synthesis of triglycerol citrate (TGC) was confirmed by detailed ^{1}H and ^{13}C nuclear magnetic resonance (NMR) spectroscopy (Figure S1). The spectra are consistent with the proposed esterified structure and indicate high conversion of citric acid and triglycerol into the desired triester product. In the ^{1}H NMR spectrum, the most characteristic signals are observed in the region between 2.79 and 2.84 ppm, integrating for four protons and assigned to the methylene protons adjacent to the carbonyl groups (H-2 and H-5). These downfield-shifted signals confirm successful esterification, as the proximity to electron-withdrawing ester functionalities deshields these protons. The broad multiplets between 3.35-3.50 ppm (6H, H-9, H-12, and H-15) and 3.60-3.82 ppm (3H, H-8, H-11, and H-14) are attributed to the glycerol-derived backbone methylene and methine protons. The most deshielded set of signals, appearing at 3.97-4.16 ppm (4H, H-7, H-10, and H-13), corresponds to protons adjacent to esterified oxygen atoms, further supporting successful formation of ester linkages [1,2]. The distribution and integration of these signals are consistent with the expected asymmetry of the TGC structure. The ^{13}C NMR spectrum further supports the proposed structure. The signal at 170.7-174.0 ppm corresponds to ester carbonyl carbons (C-3, C-4, and C-6), confirming successful formation of citrate ester bonds [1,3]. The resonance at 43.41 ppm is assigned to methylene carbons adjacent to carbonyl groups (C-2 and C-5). Carbons in the glycerol-derived segments appear at 57.3-62.4 ppm (C-9, C-12, and C-15), 65.3-67.6 ppm (C-7, C-10, and C-13), and 69.2-71.9

ppm (C-8, C-11, and C-14), reflecting different chemical environments due to esterification [1]. The signal at 73.5-76.0 ppm is assigned to the central substituted carbon (C-3), while the downfield carbonyl region confirms multiple ester functionalities within the molecule [4]. The NMR data agree with the proposed structure of TGC, confirming successful synthesis and high functionalization of the polyol backbone [5]. The absence of unexpected signals further indicates the product purity and the minimal presence of unreacted starting materials or side products.

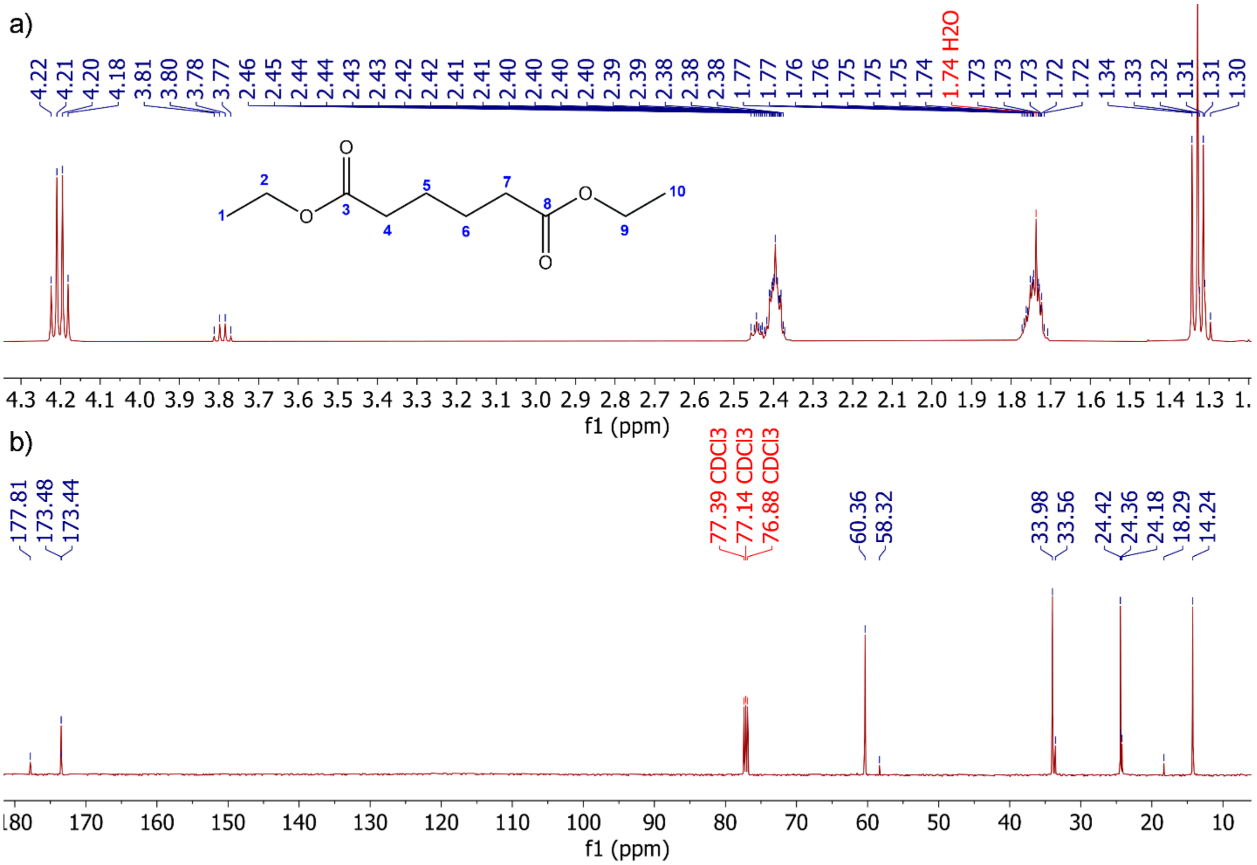

Figure S2. a) ^{1}H nuclear magnetic resonance (NMR) and b) ^{13}C NMR spectra of diethyl adipate. ^{1}H NMR ($CDCl_3$, 400 MHz, δ/ppm): 1.30-1.34 (6H, *m*, H-1 and H-10), 1.71-1.80 (4H, *m*, H-5 and H-6), 2.38-2.46 (4H, *m*, H-4 and H-7) and 3.79-4.20 (4H, *qq*, J_1=7.0 Hz, and J_2=7.1 Hz, H-2 and H-9). ^{13}C NMR ($CDCl_3$, 100 MHz, δ/ppm): 14.2-18.3 (C(1) and C(10)), 24.2-24.4 (C(1) and C(10)), 33.6-34.0 (C(4) and C(7)), 58.3-60.4 (C(2) and C(9)), 173.4-177.8 (C(3) and C(8)).

The structure of the synthesized diethyl adipate (DEA) was confirmed by both ^{1}H NMR and ^{13}C NMR spectroscopy, which showed signals consistent with the expected ester functionality and aliphatic chain symmetry (Figure S2). The ^{1}H NMR spectrum (Figure S2a) exhibited the characteristic resonances of DEA. A triplet observed at δ ~1.25-1.30 ppm (6H) was assigned to the terminal methyl protons ($–CH_3$) of the ethyl ester groups, consistent with typical ethyl ester splitting patterns due to coupling with adjacent methylene protons [6,7]. A quartet at δ ~4.10-4.20 ppm (4H) corresponded to the methylene protons directly bonded to oxygen ($–OCH_2–$), confirming the presence of ethyl ester functionalities. In the aliphatic region, overlapping multiplets observed between δ ~1.60-2.40 ppm (8H) were attributed to the internal methylene protons of the adipate backbone ($–(CH_2)_4–$), reflecting the similar chemical environment of the flexible aliphatic chain [4,6,8]. The integration ratios agree with the expected proton count for DEA, supporting

successful ester formation. Additionally, the ^{13}C NMR spectrum further confirms the structure (Figure S2b). A characteristic signal observed at δ ~172-174 ppm corresponds to the ester carbonyl carbons (C=O) of the adipate moiety. Resonances at δ ~60-61 ppm were assigned to the O–CH_2 carbons of the ethyl ester groups. Signals in the range of δ ~28-35 ppm were attributed to the internal methylene carbons of the adipate backbone (–$(CH_2)_4$–), reflecting their similar chemical environment within the flexible aliphatic chain. The terminal methyl carbons appeared at δ ~14-15 ppm, consistent with typical ethyl ester functionalities [1,3,6,7]. The number and chemical shifts of the observed carbon signals are consistent with the symmetric structure of DEA. Both ^{1}H and ^{13}C NMR spectra confirm the successful synthesis of DEA. The observed chemical shifts, integration values, and multiplicity patterns agree with database-reported data for this compound, indicating high product purity and complete esterification of adipic acid [6,7].

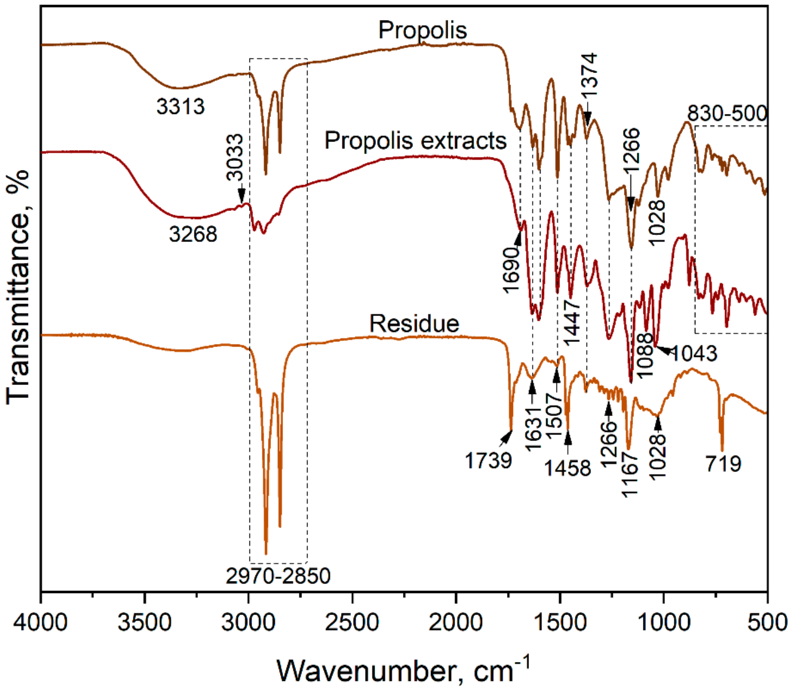

Figure S3. Fourier transform infrared spectra of crude propolis, purified propolis, and residue.

The spectra in Figure S3 show characteristic absorption bands, confirming the presence of phenolic compounds, aromatic structures, aliphatic hydrocarbons, and other components typical of the chemical composition of propolis. In the spectrum of crude propolis, a broad band around 3313 cm^{-1} is observed, which corresponds to the stretching of the O–H bond of hydroxyl groups. This band is associated with the presence of phenolic compounds, which represent one of the main bioactive fractions of propolis. The weaker band around 3033 cm^{-1} can be attributed to the stretching of the =C–H bond of aromatic rings, while the intense bands in the region of 2970-2850 cm^{-1} originate from the asymmetric and symmetric vibrations of the C–H bonds of aliphatic groups (–CH_2 and –CH_3), indicating the presence of waxes, terpenes, and lipid components. The absorption band around 1690 cm^{-1} represents the vibrations of the C=O carbonyl group and may be due to the presence of esters, ketones, and other oxygenated compounds. The bands in the region of 1600-1500 cm^{-1} (*i.e.*, around 1507 cm^{-1}) are related to the C=C vibrations of aromatic rings and are characteristic of flavonoids and phenolic acids. The bands around 1447-1374 cm^{-1} can be attributed to the deformation vibrations of the C–H groups, while the region of 1266-1028 cm^{-1} corresponds to the

vibrations of the C–O bonds of phenols, alcohols, and ether groups. In the purified propolis extract, the retention of the main characteristic bands is observed, but with changes in their intensity. The broad O–H band is shifted to around 3268 cm^{-1}, which indicates a change in the composition of hydroxyl compounds after the extraction process. Relatively stronger bands around 1690 cm^{-1}, 1447 cm^{-1}, 1266 cm^{-1}, and 1028 cm^{-1} indicate an increased concentration of polar bioactive components, particularly phenolic compounds, such as flavonoids, due to the removal of some waxes and ballast substances. Compared to the initial propolis, the extract shows a decrease in the relative contribution of aliphatic C–H vibrations, which may indicate the removal of some of the waxy fraction during purification. This is expected because extraction processes favor the transfer of phenolic and other polar compounds into the extract, while most of the hydrophobic components remain removed. The spectrum of the residue after extraction shows a different profile. The pronounced band around 1739 cm^{-1} indicates the presence of carbonyl compounds, most likely esters from waxes and lipid components. The bands around 1631 cm^{-1} and 1507 cm^{-1} may be related to aromatic structures that remain in the matrix. However, more pronounced bands in the region of 1167-1028 cm^{-1} indicate the presence of C–O functional groups, while the peak around 719 cm^{-1} may correspond to deformation vibrations of long aliphatic chains, typical of waxes. The presence of a band in the region of 830-500 cm^{-1} in the spectra of propolis and extract may be associated with deformation vibrations of aromatic C–H bonds out of the ring plane, characteristic of substituted aromatic structures.

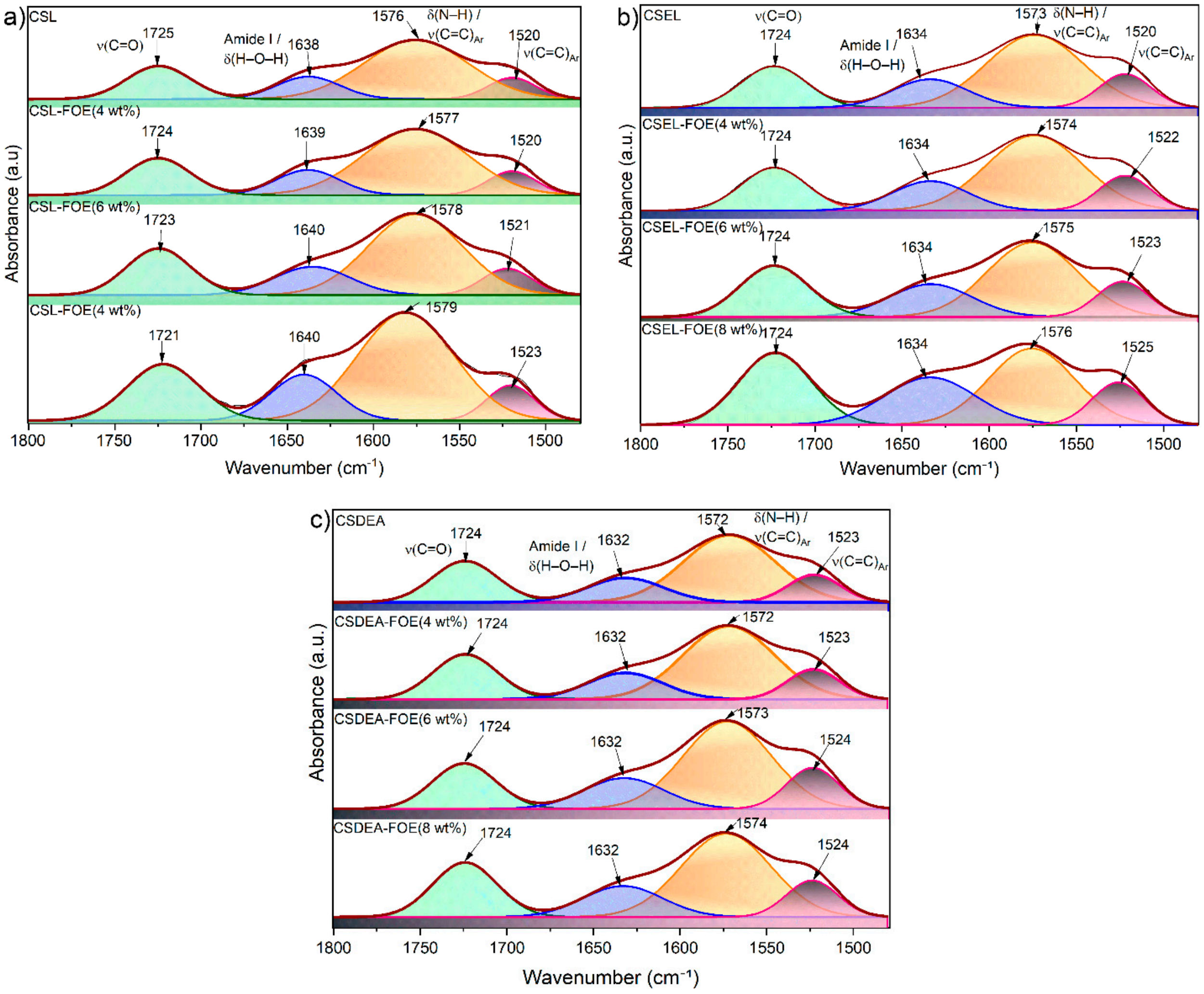

Figure S4. Deconvolution of the 1800-1480 cm^{-1} Fourier transform infrared (FTIR) region of (a) CSL, (b) CSEL, and (c) CSDEA film series; FOE - *Fumaria officinalis* extract, CS - chitosan, L - limonene, EL - ethyl lactate, and DEA - diethyl adipate.

Table S3. Results of the Fourier transform infrared (FTIR) peak deconvolution of the CSL, CSEL, and CSDEA film series.

Sampes	Deconvoluted peaks	X (cm^{-1})	Area (cm^2)	Area%
CSL	Peak 1	1725	3.715	19.75
	Peak 2	1638	2.500	13.30
	Peak 3	1576	10.798	57.42
	Peak 4	1520	1.794	9.54
CSL-FOE (4 wt%)	Peak 1	1724	3.770	19.89
	Peak 2	1639	2.542	13.41
	Peak 3	1577	10.825	57.11
	Peak 4	1520	1.815	9.58
CSL-FOE (6 wt%)	Peak 1	1723	4.309	21.12
	Peak 2	1640	3.130	15.34
	Peak 3	1578	11.047	54.15
	Peak 4	1521	1.913	9.38
CSL-FOE (8 wt%)	Peak 1	1721	4.736	21.19
	Peak 2	1640	3.512	15.71
	Peak 3	1579	12.151	54.36
	Peak 4	1523	1.952	8.73
CSEL	Peak 1	1724	4.577	19.91
	Peak 2	1634	3.620	15.75
	Peak 3	1573	11.869	51.63
	Peak 4	1520	2.924	12.72
CSEL-FOE (4 wt%)	Peak 1	1724	4.700	20.57
	Peak 2	1634	3.640	15.93
	Peak 3	1574	11.573	50.66
	Peak 4	1522	2.931	12.83
CSEL-FOE (6 wt%)	Peak 1	1724	5.901	23.65
	Peak 2	1634	4.575	18.34
	Peak 3	1575	11.400	45.70
	Peak 4	1523	3.071	12.31
CSEL-FOE (8 wt%)	Peak 1	1724	6.921	28.43
	Peak 2	1634	5.757	23.49
	Peak 3	1576	8.687	35.44
	Peak 4	1525	3.14	12.81
CSDEA	Peak 1	1724	5.510	21.95
	Peak 2	1632	3.827	15.24
	Peak 3	1572	12.787	50.94
	Peak 4	1523	2.978	11.86
CSDEA-FOE (4 wt%)	Peak 1	1724	5.577	21.98
	Peak 2	1632	3.865	15.23
	Peak 3	1572	12.882	50.76
	Peak 4	1523	3.052	12.03
CSDEA-FOE (6 wt%)	Peak 1	1724	5.737	22.27
	Peak 2	1632	4.046	15.70
	Peak 3	1573	12.912	50.12

	Peak 4	1524	3.068	11.91
CSDEA-FOE (8 wt%)	Peak 1	1724	5.875	21.96
	Peak 2	1632	4.164	15.56
	Peak 3	1574	13.107	48.98
	Peak 4	1524	3.613	13.50

FOE - *Fumaria officinalis* extract, CS - chitosan, L - limonene, EL - ethyl lactate, and DEA - diethyl adipate.

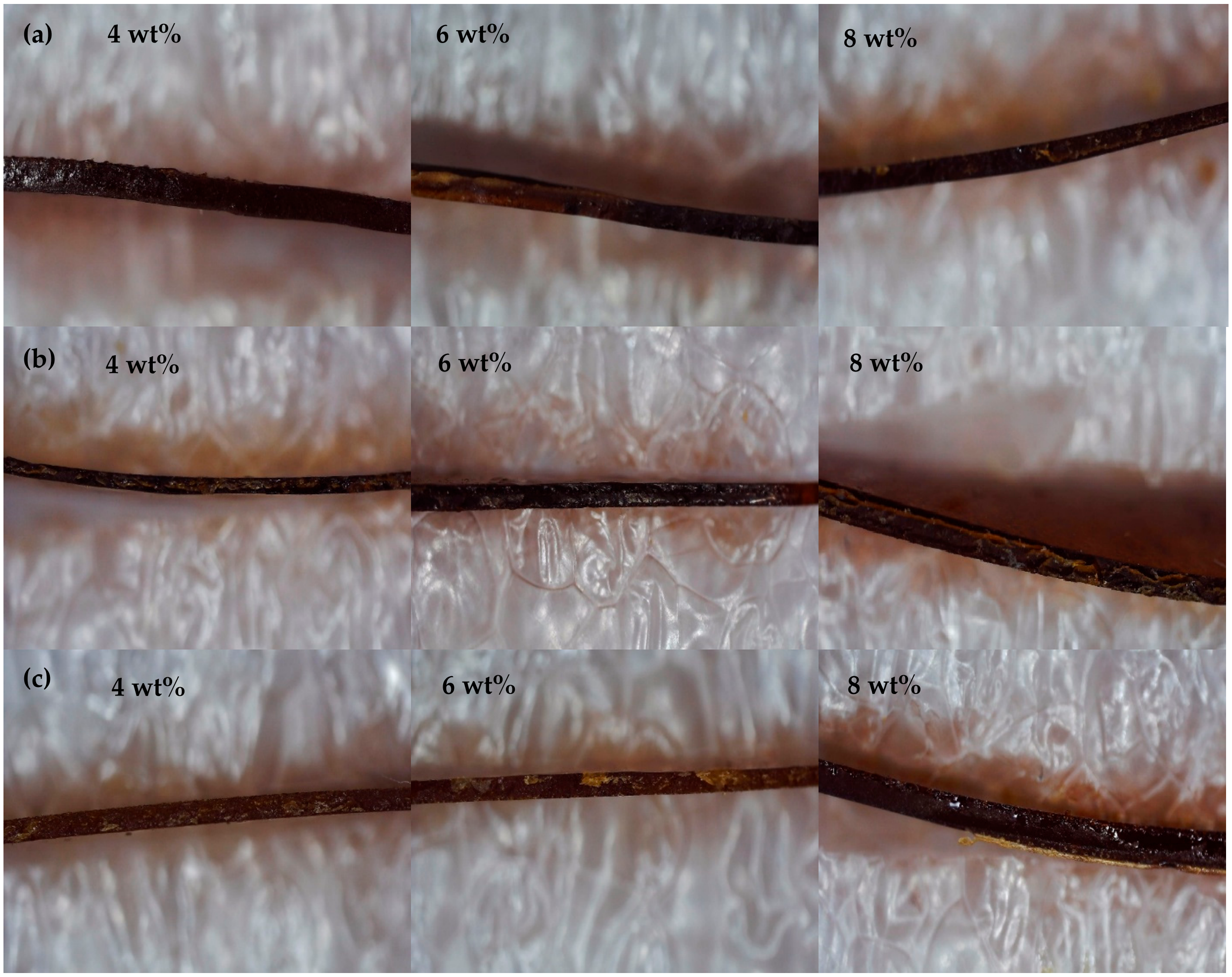

Figure S5. Optical micrographs of developed biobased films with *Fumaria officinalis* extract (FOE) and propolis cross-section: (a) CSL-FOE (4-8 wt%), (b) CSEL-FOE (4-8 wt%), and (c) CSDEA-FOE (4-8 wt%); CS - chitosan, L - limonene, EL - ethyl lactate, and DEA - diethyl adipate.

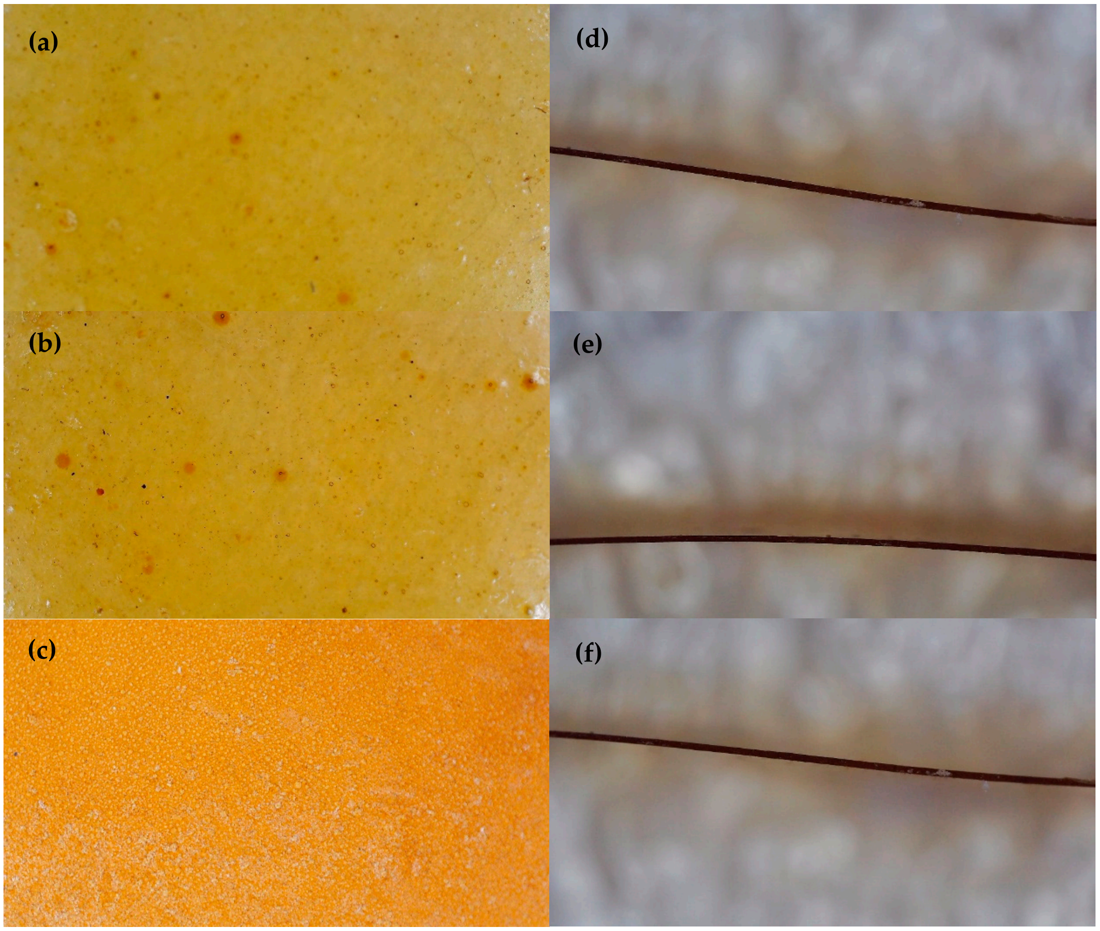

Figure S6. Images of optical microscopy analysis of plain biobased films: surface (a) CSL, (b) CSEL, and (c) CSDEA, and cross-section (d) CSL, (e) CSEL, and (f) CSDEA; CS - chitosan, L - limonene, EL - ethyl lactate, and DEA - diethyl adipate.

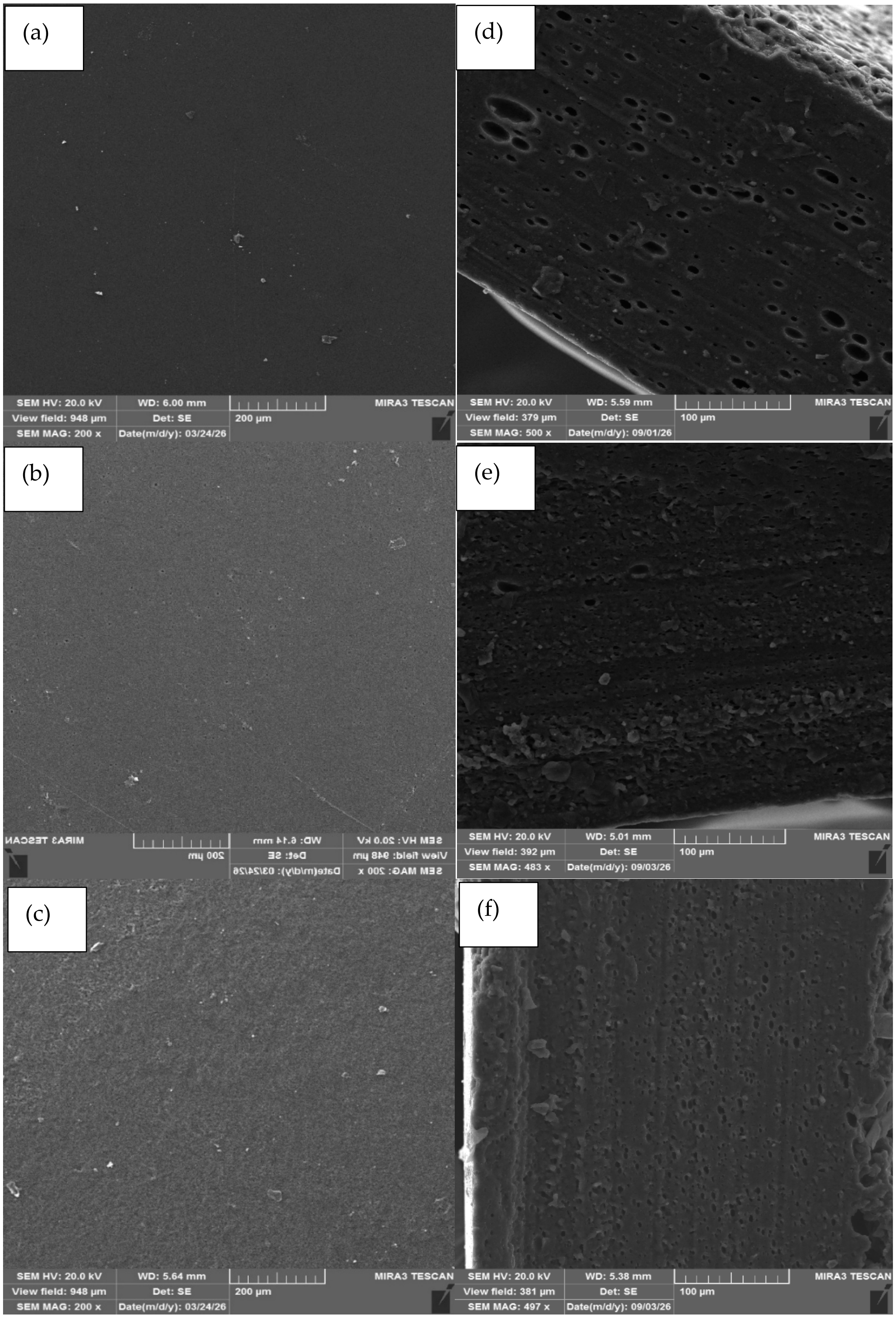

Figure S7. Images of field emission scanning electron microscopy analysis of plain biobased films: surface (a) CSL, (b) CSEL, and (c) CSDEA, 200× magnification; cross-section (d) CSL, (e) CSEL, and (f) CSDEA, 500× magnification; CS - chitosan, L - limonene, EL - ethyl lactate, and DEA - diethyl adipate.

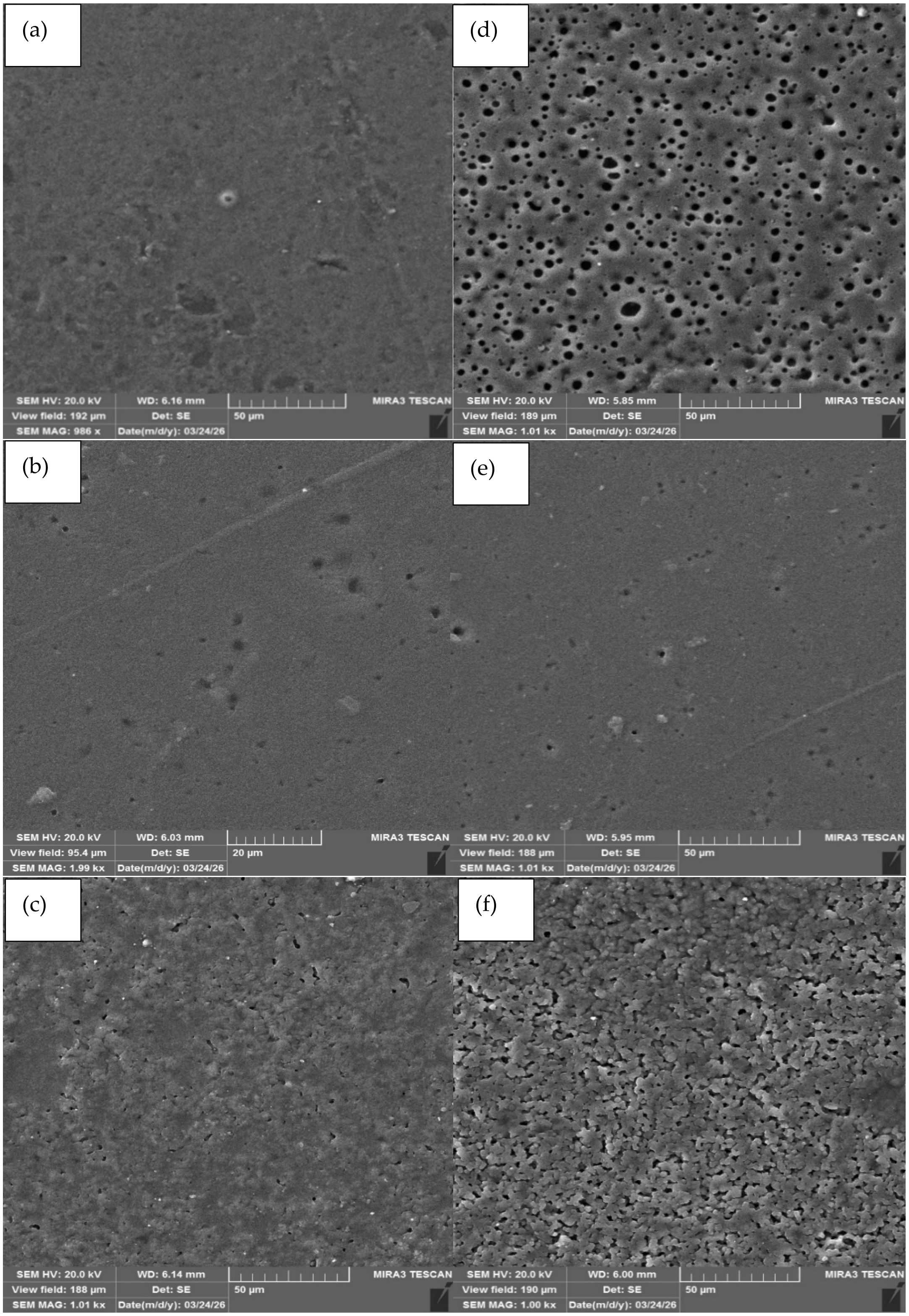

Figure S8. Images of field emission scanning electron microscopy analysis of the surface of developed biobased films with *Fumaria officinalis* extract (FOE) and propolis: (a) CSL-FOE (4 wt%), (b) CSEL-FOE (4 wt%), (c) CSDEA-FOE (4 wt%), (d) CSL-FOE (6 wt%), (e) CSEL-FOE (6 wt%), and (f) CSDEA-FOE (6 wt%);

CS - chitosan, L - limonene, EL - ethyl lactate, and DEA - diethyl adipate.

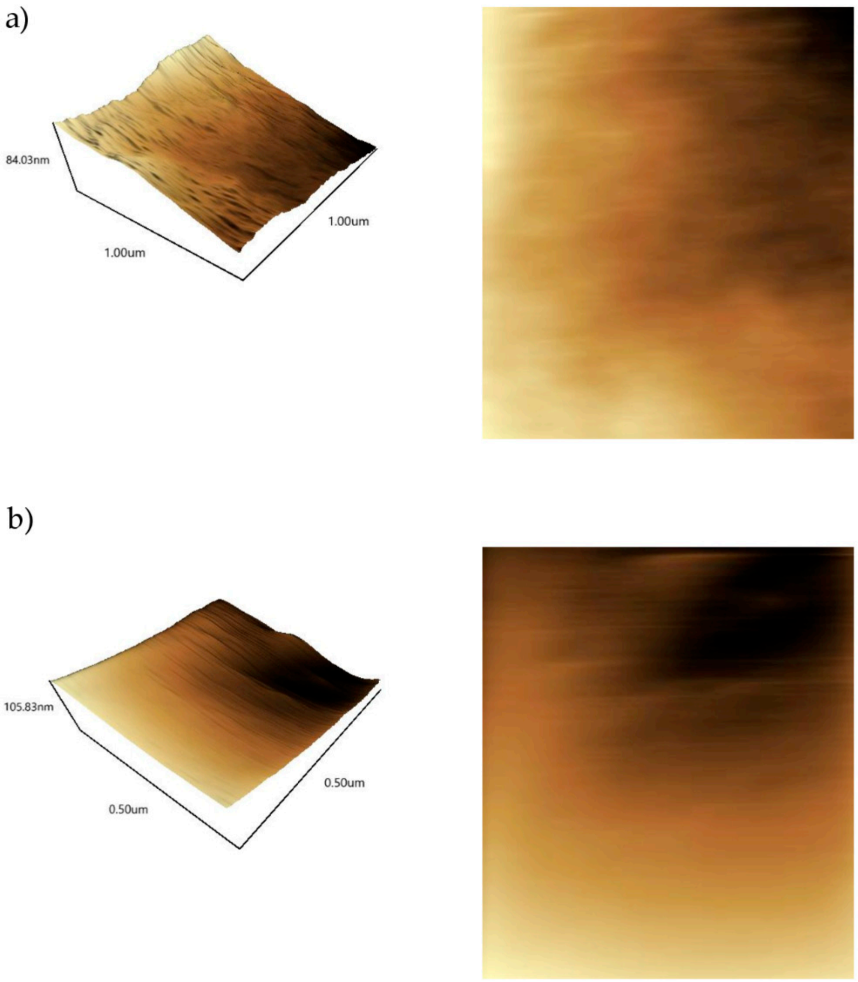

Figure S9. Atomic force microscopy topography images of the CSEL film with *Fumaria officinalis* extracts containing (a) crude propolis or (b) purified propolis. The left panels show three-dimensional surface topography, while the right panels show the corresponding two-dimensional height maps.

Table S4. Two-way analysis of variance (ANOVA) for the effects of additive type, *Fumaria officinalis* extract (FOE) concentration (4-8 wt%), and their interaction on thickness.

Source	SS	df	MS	F	p-value
Additive type	0.0327447	2	0.0163723	6.4066	0.0079
FOE concentration	0.0017847	2	0.0008923	0.3492	0.7099
Additive × FOE	0.0006793	4	0.0001698	0.0665	0.9912
Error	0.0460000	18	0.0025556	–	–
Total	0.0812087	26	–	–	–

Table S5. Two-way analysis of variance (ANOVA) for the effects of additive type, *Fumaria officinalis* extract (FOE) concentration (4-8 wt%), and their interaction on stress at break.

Source	SS	df	MS	F	p-value
Additive type	1053.6819	2	526.8409	318.3543	<0.0001
FOE concentration	2358.9905.911	2	1179.4952	712.7338	<0.0001
Additive × FOE	181.3031	4	45.3258	27.3890	<0.0001
Error	29.7880	18	1.6549	-	-
Total	3623.7635	26	-	-	-

Table S6. Two-way analysis of variance (ANOVA) for the effects of additive type, *Fumaria officinalis* extract (FOE) concentration (4-8 wt%), and their interaction on strain at break.

Source	SS	df	MS	F	p-value
Additive type	90.3398	2	45.1699	36.9186	<0.0001
FOE concentration	46.7114	2	23.3557	19.0893	<0.0001
Additive × FOE	11.4092	4	2.8523	2.3313	0.0951
Error	22.0230	18	1.2235	-	-
Total	170.4834	26	-	-	-

Table S7. Two-way analysis of variance (ANOVA) for the effects of additive type, *Fumaria officinalis* extract (FOE) concentration (4-8 wt%), and their interaction on water contact angle.

Source	SS	df	MS	F	p-value
Additive type	102.9800	2	51.4900	12.4472	0.0004
FOE concentration	162.7400	2	81.3700	19.6704	<0.0001
Additive × FOE	66.5000	4	16.6250	4.0189	0.0168
Error	74.4600	18	4.1367	-	-
Total	406.6800	26	-	-	-

Table S8. Two-way analysis of variance (ANOVA) for the effects of additive type, *Fumaria officinalis* extract (FOE) concentration (4-8 wt%), and their interaction on swelling ratio at different incubation times.

Time	Source	SS	df	MS	F	p-value
30 min	Additive	31.889	2	15.944	21.868	<0.0001
	FOE concentration	36.853	2	18.427	25.273	<0.0001
	Additive × FOE	0.577	4	0.144	0.198	0.9362
	Error	13.124	18	0.729	-	-
60 min	Additive	18.872	2	9.436	10.584	0.0009
	FOE concentration	36.452	2	18.226	20.442	<0.0001
	Additive × FOE	5.464	4	1.366	1.532	0.2352
	Error	16.049	18	0.892	-	-
90 min	Additive	21.847	2	10.923	15.287	0.0002
	FOE concentration	68.151	2	34.076	47.687	<0.0001
	Additive × FOE	4.598	4	1.150	1.609	0.2205
	Error	11.433	16	0.715	-	-
120 min	Additive	28.646	2	14.323	19.754	<0.0001
	FOE concentration	0.000	2	0.000	0.000	1.0000
	Additive × FOE	3.301	4	0.825	1.138	0.3787
	Error	10.151	14	0.725	-	-
150 min	Additive	28.746	2	14.373	13.329	0.0006
	FOE concentration	0.000	2	0.000	0.000	1.0000
	Additive × FOE	2.355	4	0.589	0.546	0.7049
	Error	15.096	14	1.078	-	-

Table S9. Release kinetic models and corresponding mathematical equations

Model	Equation (Eq.)	No. Eq.
Zero-order	$F_t = k_0 t$	(S1)
First-order	$F_t = F_\infty[1 - \exp(-k_1 t)]$	(S2)
Higuchi	$F_t = k_H t^{1/2}$	(S3)
Korsmeyer-Peppas	$F_t = k_{KP} t^n$	(S4)

k_0 (min^{-1}), k_1 (min^{-1}), k_H ($min^{-0.5}$), and k_{KP} (min^{-n}) are the zero-order, first-order, Higuchi, and Korsmeyer-Peppas release rate constants, respectively; t (min) is the release time; n is the diffusional exponent or release exponent that characterizes the specific transport mechanism.

S1. Potential Antioxidant Mechanisms of Bioactive Constituents

The antioxidant properties of the bioactive constituents incorporated into the chitosan films can be related to the chemical characteristics of their phenolic structures. Propolis contains a complex mixture of phenolic compounds, including flavonoids and hydroxycinnamic acid derivatives [8], while *Fumaria officinalis* extracts have been reported to contain flavonoids, flavonol glycosides, hydroxycinnamic acid derivatives, and isoquinoline alkaloids [9]. The coexistence of these compounds provides a chemically diverse pool of constituents potentially contributing to the antioxidant capacity measured in the present study.

S1.1. Antioxidant mechanisms of phenolic constituents

Phenolic compounds can exhibit antioxidant activity through several complementary chemical mechanisms, most notably hydrogen atom transfer (HAT) and single-electron transfer (SET). In HAT reactions, a phenolic hydroxyl group can donate a hydrogen atom to a reactive radical, thereby terminating radical chain reactions. In SET processes, electron transfer from the phenolic compound to an oxidizing species can contribute to its reduction. The resulting phenoxyl radical can be stabilized through resonance delocalization within the aromatic and conjugated structures of the molecule, thereby reducing the probability of further propagation reactions. The structural features of individual phenolic compounds strongly influence these properties. Flavonoids containing catechol-type structures, particularly those with adjacent hydroxyl groups on the B-ring, have a greater capacity for radical stabilization and electron donation than compounds lacking this structural feature [10]. Quercetin, for example, contains a 3′,4′-dihydroxy arrangement that facilitates resonance stabilization of the resulting phenoxyl radical. Other flavonoids, such as apigenin, galangin, and chrysin, possess different hydroxylation patterns and consequently may exhibit different radical-scavenging and reducing capacities [11]. Phenolic compounds may also interact with transition-metal ions. Structural arrangements involving hydroxyl and carbonyl groups, as well as catechol-type moieties, can contribute to metal binding and may influence metal-catalyzed oxidative reactions. Hydroxycinnamic acids, including caffeic, ferulic, and *p*-coumaric acid derivatives, can similarly participate in HAT- and SET-related antioxidant reactions. In particular, the catechol structure of caffeic acid and related derivatives can enhance radical stabilization and provide potential metal-binding sites [12-14]. These chemical characteristics provide a plausible molecular basis for the antioxidant responses observed in conventional chemical assays such as DPPH, ABTS, and CUPRAC. Nevertheless, the present study did not experimentally determine the contribution of individual compounds or distinguish between the specific molecular mechanisms underlying the measured antioxidant responses. Therefore, the mechanisms described here should be regarded as literature-based biochemical background rather than as mechanisms directly demonstrated by the present experiments.

S1.2. Phenolic constituents of *Fumaria officinalis*

The *F. officinalis* extract used in the present study contains a complex mixture of phenolic constituents, including flavonoids and flavonol glycosides such as rutin, isoquercitrin, quercetin glycosides, and methylquercetin derivatives, together with hydroxycinnamic acid derivatives, including caffeoylmalic acid and chlorogenic acid-related compounds [9]. The antioxidant properties of these constituents can be influenced by the number and position of hydroxyl groups, degree of glycosylation, conjugation, and molecular structure. Quercetin-derived compounds contain structural features associated with radical

stabilization and electron donation, whereas hydroxycinnamic acid derivatives may contribute through hydrogen donation, electron transfer, and, depending on their structure, interactions with transition-metal ions. Because the extract is a multicomponent system, the measured antioxidant activity represents the combined chemical response of its constituents rather than the activity of a single purified compound. Consequently, the present data cannot be used to assign the measured DPPH, ABTS, or CUPRAC responses to individual compounds within the *F. officinalis* extract.

S1.3. Phenolic constituents of propolis

Propolis is a chemically complex natural product whose composition depends on botanical origin and other factors. The propolis investigated in the present work contained flavonoid and hydroxycinnamic acid derivatives identified by phytochemical characterization. Compounds such as chrysin, galangin, quercetin, and apigenin have structural characteristics that may contribute to radical-scavenging and reducing activity. Among these compounds, differences in hydroxylation patterns are particularly relevant to antioxidant behavior. Flavonoids containing catechol-type arrangements may provide enhanced radical stabilization, whereas compounds with fewer hydroxyl groups may exhibit different relative contributions to chemical antioxidant assays. Hydroxycinnamic acid derivatives can additionally contribute through HAT and SET processes, with caffeic acid derivatives being particularly relevant because of their catechol functionality [8,10,12-15]. The presence of these compounds therefore provides a plausible chemical explanation for the antioxidant capacity associated with propolis-containing formulations. However, as the present study evaluated the formulated extracts rather than isolated compounds, no direct attribution of antioxidant activity to a particular propolis constituent can be made.

S1.4. Potential biological antioxidant pathways: literature context

Phenolic compounds have also been reported in the literature to influence cellular antioxidant and inflammatory pathways, including the nuclear factor erythroid 2-related factor 2 (Nrf2) pathway and nuclear factor kappa B (NF-κB)-associated signaling. Activation of Nrf2 can promote the expression of endogenous antioxidant and cytoprotective enzymes, including superoxide dismutase, catalase, glutathione peroxidase, and heme oxygenase-1, whereas modulation of NF-κB signaling has been associated with changes in the expression of several inflammatory and oxidative-stress-related mediators [15]. These biological effects, however, should be distinguished from the chemical antioxidant activity measured in the present study. No cellular, molecular, enzymatic, or oxidative-stress model was employed in the present work, and Nrf2, NF-κB, SOD, CAT, GPx, HO-1, iNOS, COX-2, or related pathways were not experimentally evaluated. Accordingly, these pathways are included here solely to provide literature-based context for the potential biological relevance of phenolic compounds and should not be interpreted as evidence that the developed films exert these effects.

S1.5. Co-incorporation of *Fumaria officinalis* extract and propolis

The simultaneous incorporation of *F. officinalis* extract and propolis introduces chemically diverse bioactive constituents into the chitosan matrix and may contribute to the overall antioxidant capacity of the combined formulations. To further distinguish their individual contributions, additional CSEL-based single-component control films were prepared for the DPPH assay: one containing FOE at the same absolute

loading as the CSEL-FOE (6 wt%) formulation but without propolis, and one containing the corresponding amount of purified propolis but without FOE. These additional controls enabled comparison of the individual antioxidant responses of FOE and propolis with that of the combined formulation. However, these experiments were limited to one matrix system and one antioxidant assay, and no formal synergy analysis was performed. Therefore, the present results do not establish synergistic interactions between FOE and propolis, and the observed antioxidant effects are interpreted in terms of their individual and combined contributions.

S1.6. Relationship between matrix structure, release, and antioxidant functionality

Within the developed chitosan films, the measured antioxidant capacity is closely associated with the availability and release of incorporated bioactive compounds. Matrix-modifying agents can alter polymer–bioactive interactions, chain mobility, free volume, hydration, swelling, and diffusion pathways, thereby affecting the rate and extent at which phenolic constituents become available to the surrounding aqueous medium.

Dynamic mechanical analysis demonstrated that the plasticizing agents investigated in this study produced distinct changes in the viscoelastic behavior of the chitosan matrix. Ethyl lactate, diethyl adipate, and limonene differed in their chemical polarity and interactions with the polymer network, resulting in different matrix responses. These differences were accompanied by formulation-dependent swelling and release behavior. The relatively higher release observed for ethyl-lactate-containing formulations can be considered in the context of increased matrix mobility and hydration, which may facilitate the diffusion of water-soluble bioactive constituents. In contrast, the more hydrophobic character of limonene-containing systems may restrict aqueous penetration and consequently modify the release profile. Diethyl adipate produced an intermediate behavior, consistent with a balance between matrix plasticization, structural integrity, and bioactive mobility. The temporal similarity between phenolic release and the measured antioxidant responses further supports the interpretation that antioxidant functionality is influenced by the availability of released phenolic constituents. Nevertheless, this association does not establish a specific molecular mechanism of antioxidant action. Rather, it demonstrates a formulation-structure-release-function relationship in which matrix properties regulate the accessibility of chemically active bioactive constituents. Thus, the antioxidant activity of the developed films should therefore be interpreted primarily as a functional property resulting from the incorporation and release of natural bioactive compounds within the chitosan matrix. The mechanistic pathways discussed in this section provide biochemical context based on the known chemistry of phenolic compounds, while direct biological antioxidant mechanisms remain beyond the scope of the present experimental design.

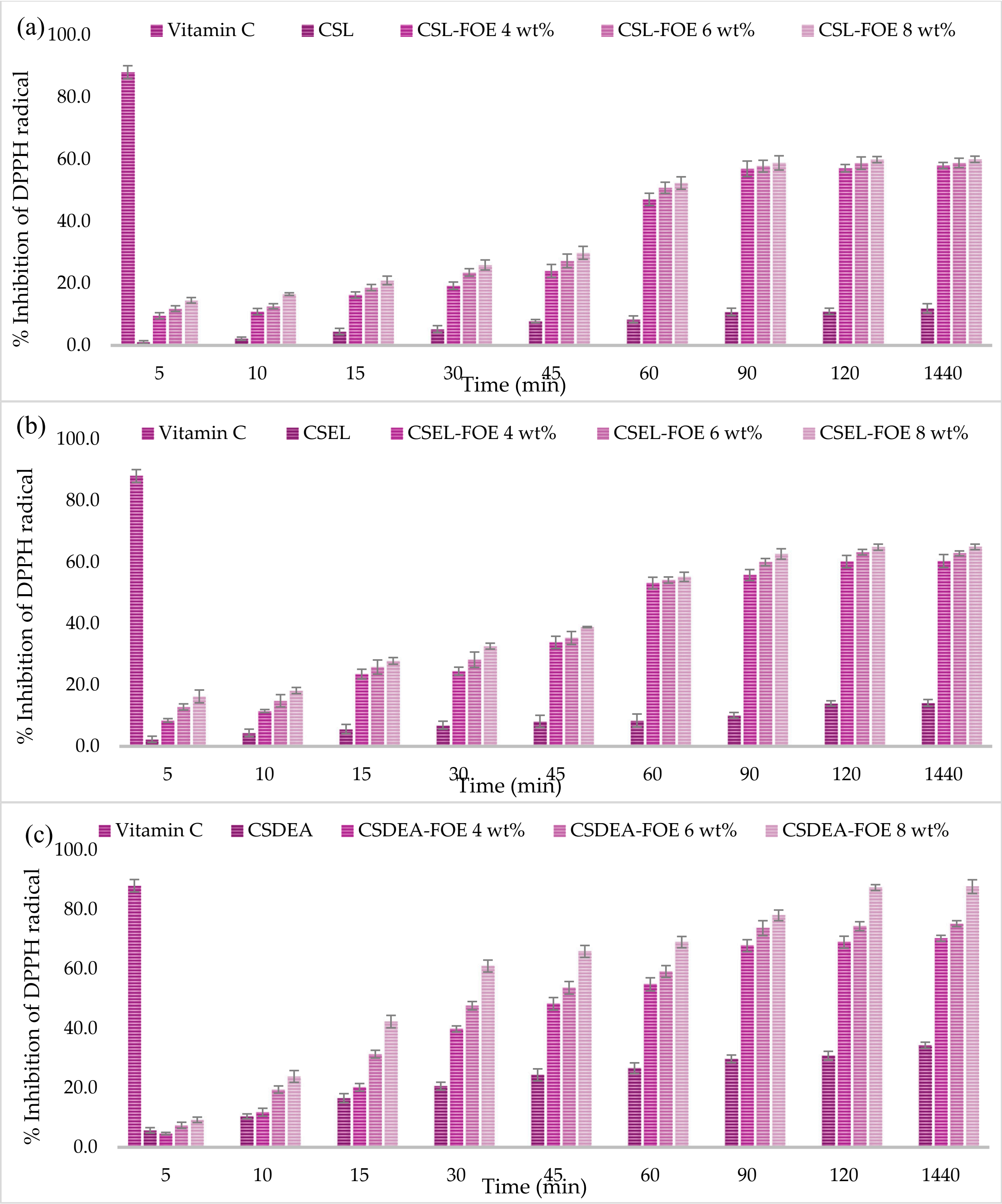

Figure S10. Anti-DPPH potential of developed biobased films with *Fumaria officinalis* extract (FOE) and crude (non-purified) propolis: (a) CSL and CSL-FOE (4-8 wt%), (b) CSEL and CSEL-FOE (4-8 wt%), and (c) CSDEA and CSDEA-FOE (4-8 wt%); CS - chitosan, L - limonene, EL - ethyl lactate, DEA - diethyl adipate; inhibition percentage *versus* time (phosphate-buffered saline, pH 7, ambient temperature).

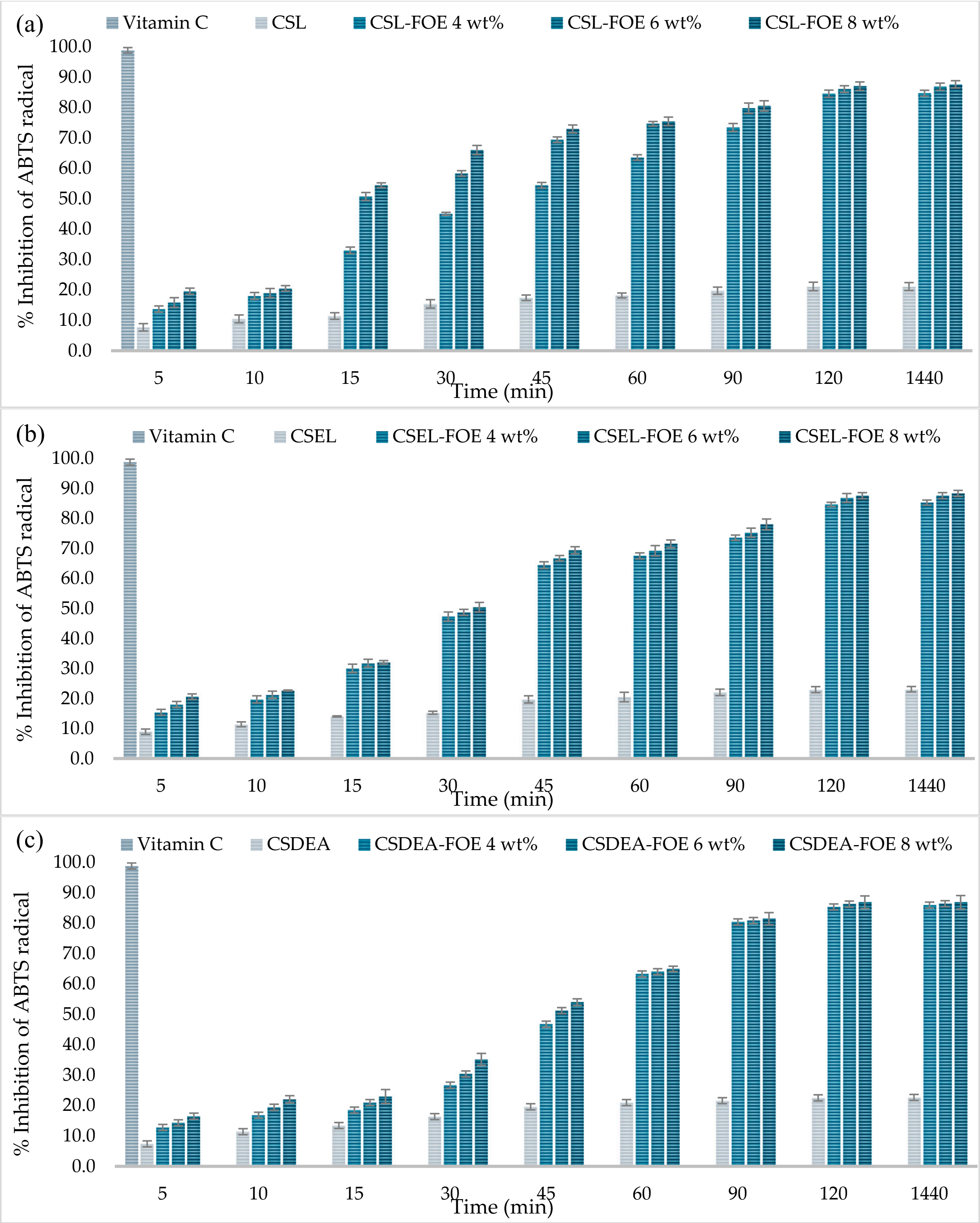

Figure S11. Anti-ABTS potential of developed biobased films with *Fumaria officinalis* extract (FOE) and crude (non-purified) propolis: (a) CSL and CSL-FOE (4-8 wt%), (b) CSEL and CSEL-FOE (4-8 wt%), and (c) CSDEA and CSDEA-FOE (4-8 wt%); CS - chitosan, L - limonene, EL - ethyl lactate, DEA - diethyl adipate; inhibition percentage *versus* time (phosphate-buffered saline, pH 7, ambient temperature).

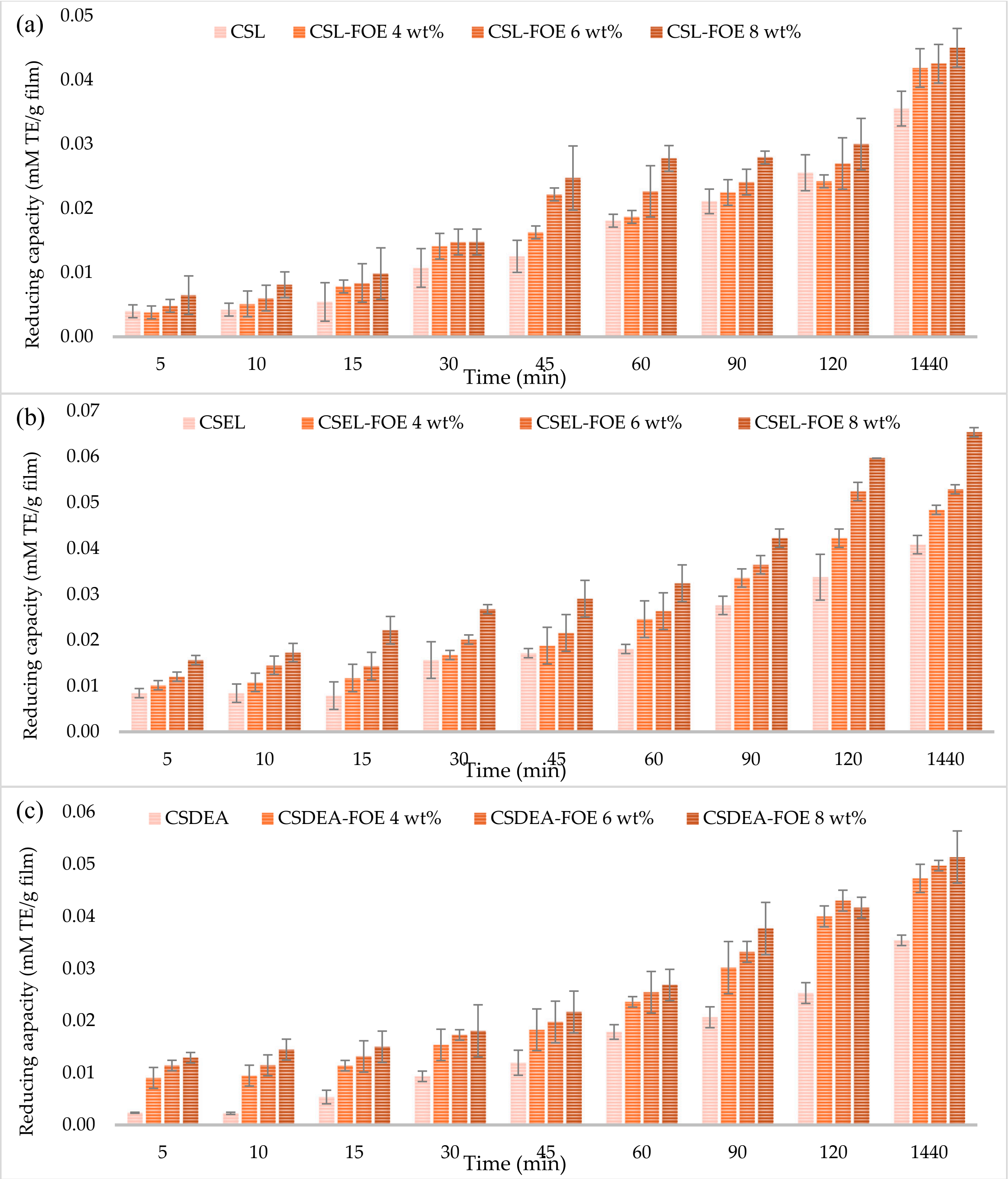

Figure S12. Cupric reducing antioxidant capacity of developed biobased films with *Fumaria officinalis* extract (FOE) and crude (non-purified) propolis: (a) CSL and CSL-FOE (4-8 wt%), (b) CSEL and CSEL-FOE (4-8 wt%), and (c) CSDEA and CSDEA-FOE (4-8 wt%); CS - chitosan, L - limonene, EL - ethyl lactate, DEA - diethyl adipate; reducing capacity *versus* time (phosphate-buffered saline, pH 7, ambient temperature); TE, Trolox equivalent.

References

1. Li, Q.; Shu, X.; Jia, P.; Zhou, Y. Preparation of biomass-based ester end-capped hyperbranched poly(ether)s *via* facile one-pot reaction and their performance as non-toxic plasticizers. *Polymers* **2020**, *12*, 913. https://doi.org/10.3390/polym12040913
2. Mahmud, H.A.; Salih, N.; Salimon, J. Oleic acid based polyesters of trimethylolpropane and pentaerythritol for biolubricant application. *Malays. J. Anal. Sci.* **2015**, *19*, 97–105.
3. Giroto, A.S.; Valle, S.F.; Borges, R.; Colnago, L.A.; Ribeiro, T.S.; Jablonowski, N.D.; Ribeiro, C.; Mattoso, L.H.C. Revealing the structure formation on polyglycerol citrate polymers – an environmentally friendly polyester as a seed-coating material. *Polymers* **2023**, *15*, 4303. https://doi.org/10.3390/polym15214303
4. Lau, C.C.; Bayazit, M.K.; Knowles, J.C.; Tang, J. Tailoring degree of esterification and branching of poly(glycerol sebacate) by energy efficient microwave irradiation. *Polym. Chem.* **2017**, *8*, 3937–3947. https://doi.org/10.1039/C7PY00862G
5. Olarte-Paredes, A.; Salgado-Delgado, A.M.; García-Fuentes, J.J.; Salgado-Delgado, R.; Cedillo-Valverde, G.; López-Lara, T.; Hernández-Zaragoza, J.B.; Castaño, V.M. Synthesis and characterization of a polyester based on citric acid/ethylene glycol/glycerol. *J. Macromol. Sci. A* **2021**, *58*, 890–898. https://doi.org/10.1080/10601325.2021.1965890
6. National Center for Biotechnology Information. PubChem Compound Summary for CID 6509, Diethyl adipate. Available online: https://pubchem.ncbi.nlm.nih.gov/compound/Diethyl-adipate (accessed on 26 May 2026).
7. Cooper, T.R.; Storey, R.F. Characterization of poly(ethyleneoxyethylene terephthalate-co-adipate) using NMR spectroscopy. *J. Macromol. Sci. A* **2007**, *44*, 367–372. https://doi.org/10.1080/10601320601187994
8. Xu, W.; Lu, H.; Yuan, Y.; Deng, Z.; Zheng, L.; Li, H. The Antioxidant and Anti-Inflammatory Effects of Flavonoids from Propolis *via* Nrf2 and NF-κB Pathways. *Foods* **2022**, 11, 2439. https://doi.org/10.3390/foods11162439
9. Ahmoda, R.A.; Pirković, A.; Milutinović, V.; Milošević, M.; Marinković, A.; Jovanović, A.A. *Fumaria officinalis* dust as a source of bioactives for potential dermal application: optimization of extraction procedures, phytochemical profiling, and effects related to skin health benefits. *Plants* **2025**, *14*, 352. https://doi.org/10.3390/plants14030352
10. Kurkiewicz, M.; Moździerz, A.; Rzepecka-Stojko, A.; Stojko, J. Chrysin: A Comprehensive Review of Its Pharmacological Properties and Therapeutic Potential. *Pharmaceuticals* **2025**, *18*, 1162. https://doi.org/10.3390/ph18081162
11. Manach, C.; Scalbert, A.; Morand, C.; Rémésy, C.; Jiménez, L. Polyphenols: Food sources and bioavailability. *Am. J. Clin. Nutr*. **2004**, 79, 727–747. https://doi.org/10.1093/ajcn/79.5.727
12. Portillo-Lemus, L.; Vernus, B.; Chabi, B.; Lebrun, A.; Cazals, G.; Rapior, S.; Fons, F.; Carnac, G.; Morel, S. French Propolis Caffeic Acid Derivatives Protect Skeletal Muscle from Oxidative Damages. *Biomolecules* **2026**, 16, 550. https://doi.org/10.3390/biom16040550.
13. Yang, H.; Dong, Y.; Du, H.; Shi, H.; Peng, Y.; Li, X. Antioxidant Compounds from Propolis Collected in Anhui, China. *Molecules* **2011**, 16, 3444–3455. https://doi.org/10.3390/molecules16043444
14. Shang, Y.-J.; Liu, B.-Y.; Zhao, M.-M. Details of the antioxidant mechanism of hydroxycinnamic acids. *Czech J. Food Sci*. **2015**, 33, 210–216. https://doi.org/10.17221/611/2014-CJFS
15. Jomova, K.; Alomar, S.Y.; Valko, R.; Liska, J.; Nepovimova, E.; Kuca, K.; Valko, M. Flavonoids and their role in oxidative stress, inflammation, and human diseases. *Chem. Biol. Interact*. **2025**, 413, 111489. https://doi.org/10.1016/j.cbi.2025.111489
